# High-resolution paleobiome reconstructions highlight ecological and historical dynamics underpinning global terrestrial vertebrate diversity

**DOI:** 10.1371/journal.pbio.3004077

**Published:** 2026-10-23

**Authors:** Alexander Skeels, Julian Rogger, Marcel Cardillo, Alexander Farnsworth, Daniel J. Lunt, Dirk Nikolaus Karger

**Affiliations:** 1 Research School of Biology, Australian National University, Canberra, Australia; 2 School of Geographical Sciences, University of Bristol, Bristol, United Kingdom; 3 Cabot Institute for the Environment, University of Bristol, Bristol, United Kingdom; 4 Dynamic Macroecology, Swiss Federal Institute for Forest, Snow and Landscape Research WSL, Birmensdorf, Switzerland; Centre National de la Recherche Scientifique, FRANCE

## Abstract

Past climate change has repeatedly reshaped Earth’s biomes, yet how their expansion and contraction influence biodiversity patterns through time remains unclear. Competing hypotheses propose that either historical biome area has a strong influence on diversity, or that biodiversity responds rapidly to environmental change, such that present-day biome area and energy are sufficient to explain diversity patterns. Progress in testing these ideas has been limited by sparse and inconsistent proxy data and the coarse resolution of existing paleoclimate models. Here, we reconstructed global biome histories for the Cenozoic (66–0 Ma) by downscaling five different paleoclimate simulations. Estimates of change in biome area through time were generally consistent among alternative climate models and with the empirical proxy record. Accounting for variation among models, we found that present-day area and energy were consistently better predictors of vertebrate diversity than historical measures. These findings suggest biodiversity tracks the ecological limits set by shifting biomes, leaving sparse legacy of past dynamics. Our results highlight the importance of integrating multiple paleoclimate reconstructions when assessing the historical drivers of biodiversity.

## Abstract 2

How past biome dynamics shape present-day biodiversity is unclear. This study uses reconstructed paleobiomes over the past 66 million years to show that current biome properties predict diversity better than historical biomes, suggesting that biodiversity rapidly tracks changing ecological limits.

## Introduction

Species diversity varies enormously among the world’s biomes, and particularly between warm, humid and high-energy tropical biomes compared to their temperate and polar counterparts. All explanations for spatial diversity gradients must invoke variation in speciation, extinction, and dispersal rates [1,2]. Most hypotheses account for this variation in two main ways: ecological or historical [3–7], based on whether diversity is best explained by environmental carrying capacities or by the legacy of historically contingent patterns of colonisation and diversification. Ecological theories propose that biodiversity patterns emerge from diversity-dependent diversification, in which diversification rates are negatively correlated with standing diversity, modulated by resource availability, which is determined by the geographic size and available energy of suitable and reachable habitat (hereafter, area and energy [8,9]). Historical theories, on the other hand, propose that if ecological limits to diversity exist at all, they are rarely reached, and therefore factors that shape variation in the rate of diversity accumulation are most important—including how long an area has been occupied and how fast speciation is relative to extinction (net diversification rates [10,11]). Ecological and historical explanations for diversity are not mutually exclusive, but endpoints along a continuum, along which areas or clades are positioned by the speed at which their diversity responds to changes in area or energy relative to how fast these changes occur [12]. Understanding whether historical climate change leaves a detectable signature on present-day diversity patterns [13–15] is critical for understanding the distribution of biodiversity among the world’s major biome types and the response of biodiversity to major climatic changes.

The global distribution of climates today is strikingly different to the conditions under which much of the diversity of life evolved. The current cool-house conditions likely arose during the Cenozoic, beginning at the Eocene-Oligocene boundary [16,17] when a CO_2_ decrease enabled Antarctic glaciation [18–20]. Before this, during the early Cenozoic, Earth’s climate was much warmer [17,21] and there is fossil evidence for tropical or near-tropical conditions at high latitudes (>50° [22,23]). This suggests that tropical conditions were much more widespread in the past than in the present, where today they are dwarfed by arid, boreal and polar biomes at a global scale [1]. On the other hand, global reconstructions of past biomes from proxy data [21] and climate models [24,25] both suggest that the extent of tropical biomes has only decreased to a relatively small degree throughout the Cenozoic, and instead the warmer conditions of the early Cenozoic are manifested in larger areas of subtropical and warm temperate biomes at high latitudes compared to cool temperate, cold-continental, and polar biomes at those latitudes in the present day. These data and models also suggest that even in hothouse climates, mesic tropical biomes are spatially constricted by arid conditions which have been persistent and widespread features of the subtropical latitudes during the Phanerozoic [21,26,27].

Understanding how biomes have changed, and whether the area of tropical biomes has been considerably larger than other biomes over the Cenozoic period (66−0 Ma), is critical to untangling ecological from historical explanations for global diversity gradients. A regular feature of many historical hypotheses for biodiversity is that biomes with high diversity today, such as tropical rainforests, have been more widespread in the past (e.g., time-area-energy hypothesis [2,14,28] and tropical conservatism hypothesis [29,30]). These hypotheses propose that tropical biomes have more species than expected given their present-day area because the larger historical area led to more clades arising and diversifying there. Therefore, past area should be a strong predictor of present-day diversity. On the other hand, ecological hypotheses propose that environmental carrying capacities of biomes are sufficient to explain present-day diversity, irrespective of past area. Biomes that are larger and more productive can sustain more individuals and a greater variety of species than smaller or less productive biomes [31]. As biome area and energy changes over time, for example if a biome contracts or expands, biodiversity should change rapidly due to changes to the regional carrying capacity via shifts in resource availability [32,33]. Whether environmental carrying capacities determine species richness, or even if they exist, has been fervently debated [34,35]. If there are no defined ecological limits on diversity then we would instead expect a strong signature of historical dynamics in present-day diversity patterns. Where biodiversity sits along the ecological-historical continuum depends not only on the presence of ecological limits, but also on the speed of response of biodiversity to climate change, and different clades in different regions may sit at different points. If biodiversity generally responds rapidly, then diversity should be more strongly dominated by contemporary conditions, and vice versa.

To date, there is ample evidence for a strong relationship between present-day environmental variables and species diversity (e.g.[36,37],). Yet studies that have directly compared explanatory power of present-day biome area to estimates of the total area integrated across time intervals during the Cenozoic, and to the minimum area achieved in the Cenozoic, show that history explains more variation in diversity of trees [15,38]. This suggests that biomes that have been formerly and persistently widespread harbour higher diversity than biomes that have been historically small or have contracted to a large degree during periods of climate change. This pattern becomes stronger when considering environmental energy, measured as the productivity of biomes accounting for area change over the Cenozoic, in terrestrial vertebrates [13]. The interpretation that the history of biome area is the primary explanation for diversity patterns rests heavily on accurate estimations of past climates which has been hitherto limited by sparsely available proxy data from the fossil and lithological record. Recent advances in models of paleoclimates for the Cenozoic [39,40] have made spatially continuous and global estimates of past climates available and have increased our capacity to accurately estimate historical biome area over the whole globe at regular time intervals. Recent studies taking this approach paint a nuanced picture of the history of global biomes, by distinguishing not only between major climate-biomes (e.g., tropical and temperate) but also between the minor variations in seasonality of precipitation and temperature that define key ecoregions under the Köppen-climate classification scheme (e.g.[24,25]). Whether including a more detailed assessment of historical biomes from climate models changes our understanding of the historical role of biome area on diversity remains unclear.

In this study, we revisit the longstanding hypothesis that historical biome area is a key driver of present-day diversity gradients using state-of-the-art climate models, downscaled here to reconstruct Köppen climate-biomes at very high spatial resolution (0.1°) over the Cenozoic period. We advance this topic by: (1) quantifying model-derived changes in biome area through time to classify their growth trajectories (expansion versus contraction); and (2) revisiting the approach taken in [13], leveraging well-sampled and globally available species distribution information for terrestrial vertebrates classes (birds, mammals, amphibians, and reptiles), to test the relative importance of present-day versus historical biome area and energy as predictors of species diversity. We emphasise that our while our biome delimitation differs from that of [13], the qualitative agreement (or disagreement) between the two is itself informative. A historical explanation for biodiversity would be supported by demonstrating (i) formerly widespread and now contracting biomes have greater species diversity than formerly restricted and now expanding biomes, and (ii) time-integrated biome area explains more variation in species richness than present-day area. On the other hand, an ecological explanation would be more strongly supported if present-day conditions better explain diversity than historical conditions, indicating little or no historical legacy.

## Results

### Reconstructed change in paleobiome area

The geographic extent of Köppen climate-biomes in the present day is at different proportions to their historical prevalence (Fig 1; Figs A–E in S1 Appendix). On average the area of tropical biomes (Af, Am, and Aw Köppen categories) has decreased through the Cenozoic. Four out of five climate models supported a mid-Eocene peak (~40 Ma; Fig 1), when the combined area of tropical biomes was estimated at 41.8 million km^2^ (range = [39.4, 45.0]), significantly larger than their present-day area of 32.7 million km^2^. HadCM3L-C, on the other hand, supported an Oligocene peak during the Rupelian stage (~33.9–27.8 Ma) of 33.3 million km^2^, similar to their present-day area (Fig C in S1 Appendix). The time-integrated summed area of each biome across climate models for the whole Cenozoic showed that tropical biomes have occupied on average 23.0% of Earth’s terrestrial surface (range = [18.8, 26.3]) and are therefore relatively underrepresented today at 20.7% (Fig 1). Similarly to tropical biomes, we also found that the time-integrated area of temperate biomes (Cf, Cs, Cw) has decreased from 18.0% [17.4, 18.6] to 11.3%, as have cold biomes (Df, Ds, Dw, Dc) from 29.4% [24.2, 34.4] to 21.9%. In contrast, we estimated an increase in arid biomes (BW, BS), from 22.6% [18.2, 26.8] to 30.4%, and polar biomes (ET, EF) from 6.9% [3.6, 13.2] to 15.7%.

**Fig 1:**
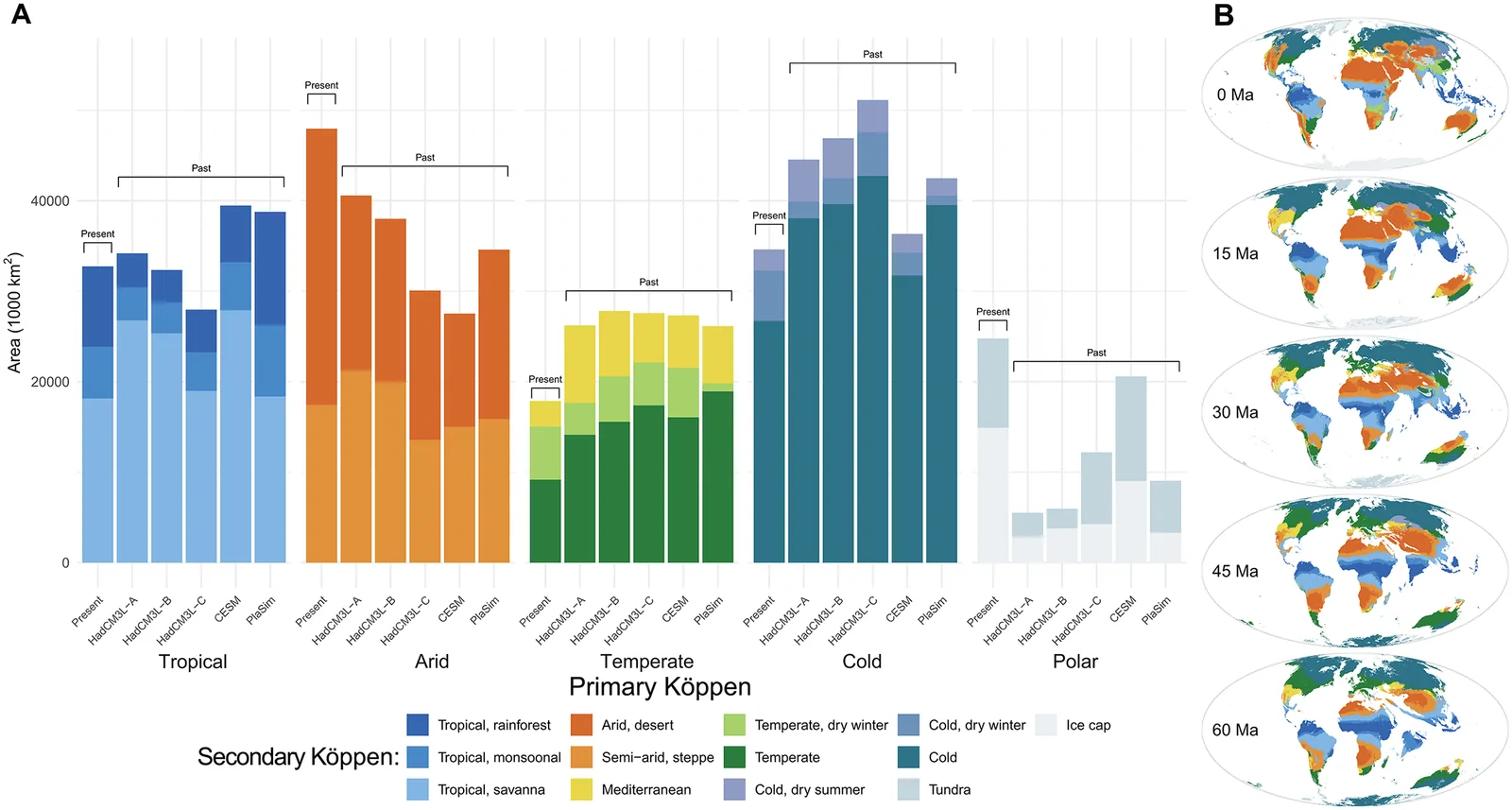
Reconstructed changes in biome area. **(A)** The proportion of each biome across Earth’s surface in the present compared to the average integrated area of biomes across the Cenozoic derived from five downscaled climate models, excluding the present-day, organised by primary Köppen classification and coloured by secondary Köppen classifications. **(B)** Downscaled paleobiome reconstructions for an example climate model (PlaSim). The data and code needed to generate this figure can be found at https://doi.org/10.5281/zenodo.17595795.

### Paleobiome model validation and congruence

We validated the climate-model-derived reconstructions of paleobiomes by comparing the primary Köppen category (A = tropical, B = arid, C = temperate, D = cold/continental, E = polar) across the climate models with primary Köppen categories from expert-drawn, proxy-based reconstructions [21] (Fig F in S1 Appendix). Following conventions for interpreting the κ metric of reclassification accuracy [41], climate-models showed moderate-to-substantial agreement based with the proxy-based reconstructions for the past 25 Ma (mean Cohen’s kappa = 0.58–0.70 across time steps; Fig G in S1 Appendix), declining to moderate agreement around the Eocene–Oligocene transition (~30 Ma; kappa = 0.43) and fair agreement in the Eocene and Paleocene (35–65 Ma; kappa = 0.25–0.38; Fig G in S1 Appendix). The PlaSim model showed the highest overall agreement (mean kappa = 0.52; Fig 1B), followed by HadCM3L models (kappa = 0.45–0.46), with CESM showing the lowest agreement (kappa = 0.35). Next, as a second validation, we compared climate-model predictions of primary Köppen category at 1,381 individual lithological proxy localities (Table A in S1 Appendix) and found that 71.6% of proxy localities were consistent with climate model predictions (Fig G in S1 Appendix), ranging from 75.0% (HadCM3L-A) to 64.2% (CESM) across models. Agreement was highest for the Oligocene-early Eocene (47.8-23.03 Ma; 75%), followed by the Neogene (23–5 Ma; 73.0%), early-mid Eocene (56–47.8 Ma; 70.5%), and 68.5% in the Paleocene (66–56 Ma). We found that even for the expert-drawn, proxy-based reconstructions, only 81.4% of proxy localities were consistent, such that our climate models achieved between 78.9% and 92.1% agreement of the proxy maps themselves (Table A and Fig G in S1 Appendix). In some cases, the climate models showed stronger agreement with the proxy localities than the proxy-based reconstruction, with, for example, all five climate models outperforming the expert-drawn proxy-based reconstructions for tropical indicators (laterites, bauxites, and mangroves; Table A and Fig G in S1 Appendix).

Looking across six biogeographic realms (Australasia, Neotropics, Afrotropics, Indomalaya, Nearctic, Palearctic [42,43]), climate models showed broadly congruent biome histories, with 39 out of 57 (68.4%) of biome–realm combinations showing consistent support for a single growth trajectory (expansion or contraction) across all models which showed a significant directional trend (Fig 2; Figs H–L in S1 Appendix). Agreement was highest for temperate and cold biomes (e.g., Cf, Dw, and Df showed 100% agreement across all realms), and lowest for tropical biomes. Agreement was also highest in the Palearctic (80%), Australasian (75%) and Afrotropical (75%) realms and lowest in the Neotropics (50%). To further validate the similarity among climate models, we estimated pairwise correlations of biome area trajectories across all five climate models. Biome area trajectories showed moderate agreement (mean pairwise Pearson *r* = 0.39 ± 0.46 SD; Table B in S1 Appendix), with the highest agreement between the two HadCM3L-A and HadCM3L-B which only differed in CO_2_ forcing (*r* = 0.86 ± 0.23) and moderate-to-high agreement among the remaining non-HadCM3L-C models (*r* = 0.54 ± 0.4). HadCM3L-C showed the lowest agreement with other models (mean *r* = 0.16 ± 0.44), consistent with its different paleogeographic boundary conditions. However, we also found that all time-integrated area estimates were highly correlated across all five models (*r* = 0.95 ± 0.03; Table C in S1 Appendix), showing that variation in trajectories among climate models did not strongly affect estimates of historical biome area over broad time scales.

**Fig 2:**
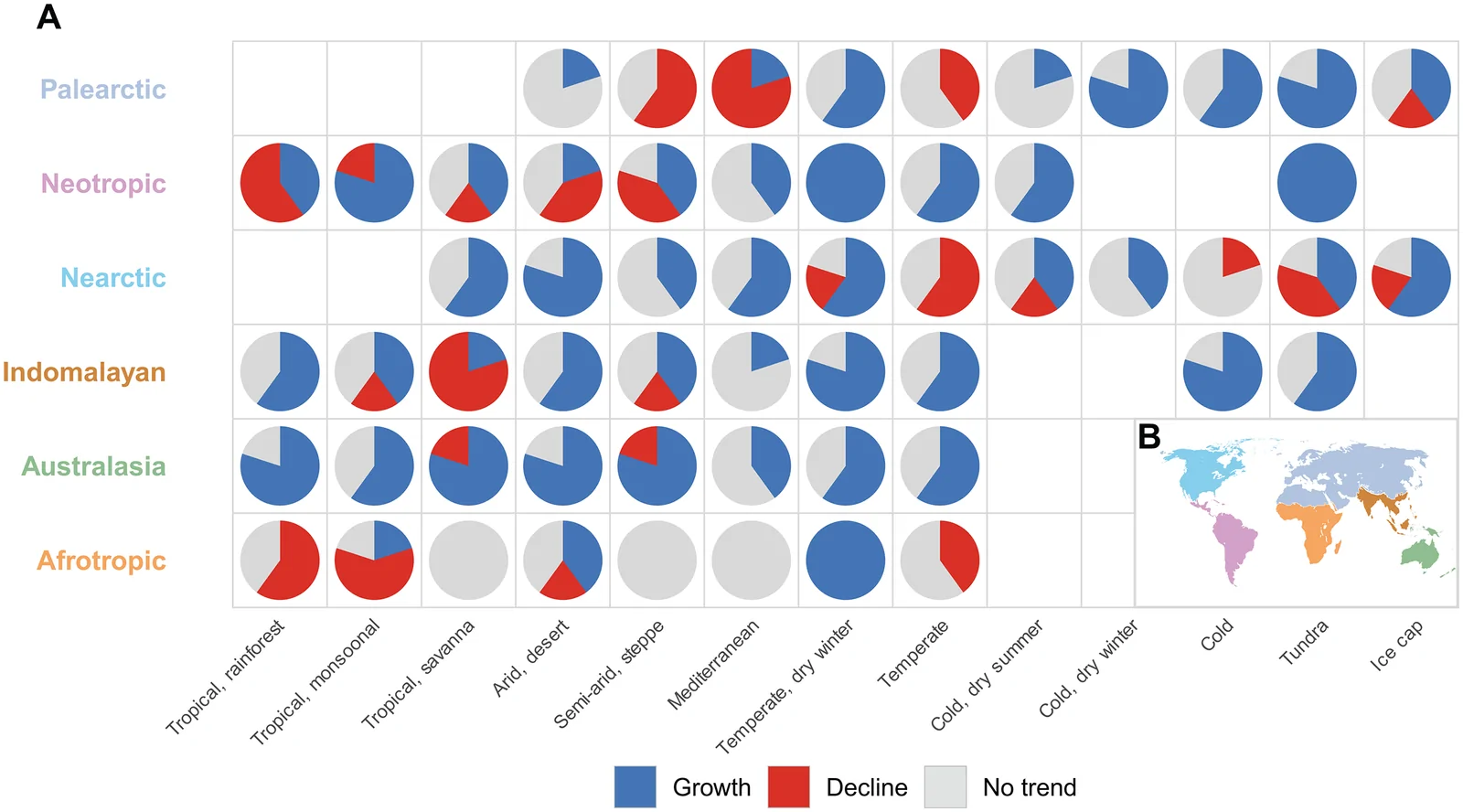
Patterns of growth and decline of Köppen biomes (A) across biogeographical realms (B). Six terrestrial biogeographic realms (Afrotropical, Australasian, Indomalayan, Nearctic, Neotropical, Palearctic [42]; https://ecoregions.appspot.com/, CC BY 4.0). Realm colours in Panel B match the realm name colours in panel **A.** The data and code needed to generate this figure can be found at https://doi.org/10.5281/zenodo.17595795.

### Present-day and historical predictors of species diversity

Compositional overlap among realm–biome units was low, with median Jaccard similarities of 0.07% in amphibians, 0.11% in reptiles, 2.10% in birds and 3.48% in mammals (Table H in S1 Appendix), such that they can be considered unique evolutionary arenas (sensu [13]). To test whether present-day or historical measures of biome area and energy were more important in explaining variation in endemic, resident, or total species richness in terrestrial vertebrate classes, we fitted a series of generalised linear mixed-effects models (GLMM). Models included either present-day measures of area or energy (area-integrated productivity and temperature) or historical measures of area (time-integrated area; timeArea), energy (time-and-area-integrated productivity; timeAreaProd), and the trajectory of biome growth (expansion, contraction, or no pattern). For historical predictors, we selected the best-fitting climate model (HadCM3L-A-C, CESM, PlaSim) and time window (65, 35, or 15 Ma) for each vertebrate class and species richness metric separately using AIC (Table D in S1 Appendix). HadCM3L-A model was consistently the best-fitting climate model for historical predictors (20 of 24 comparisons), and the 35 Ma window was consistently preferred for total richness, and the 15 Ma for resident and endemic richness (Table D in S1 Appendix).

Overall, we found that models which included present-day predictors outperformed historical measures of area and energy (Fig 3B and 3C; Table E in S1 Appendix). Specifically, we found that present-day-only models were the best-supported model in 9 of 12 comparisons across four vertebrate classes and three richness metrics. Present-day energy and area (prodArea + temp) were the preferred models for 6 of 12 comparisons (Fig 3A) and present-day area and temperature were the best fit for resident and endemic squamate reptile richness. The three exceptions where historical information improved model fit, were for total richness of birds and squamates and for endemic mammal richness in which the best model included present-day prodArea, temperature, and the trajectory of historical biome area change. Similarly, including trajectory as a covariate was a comparable fit (ΔAIC< 2; Fig 3B) for amphibian total and resident richness, and squamate resident and endemic richness. Across the best models, conditional *R*^2^ values ranged from 0.76 to 0.92 (marginal *R*^2^ 0.56–0.90), indicating that present-day area and energy explain a high proportion of variance across vertebrate richness metrics.

**Fig 3:**
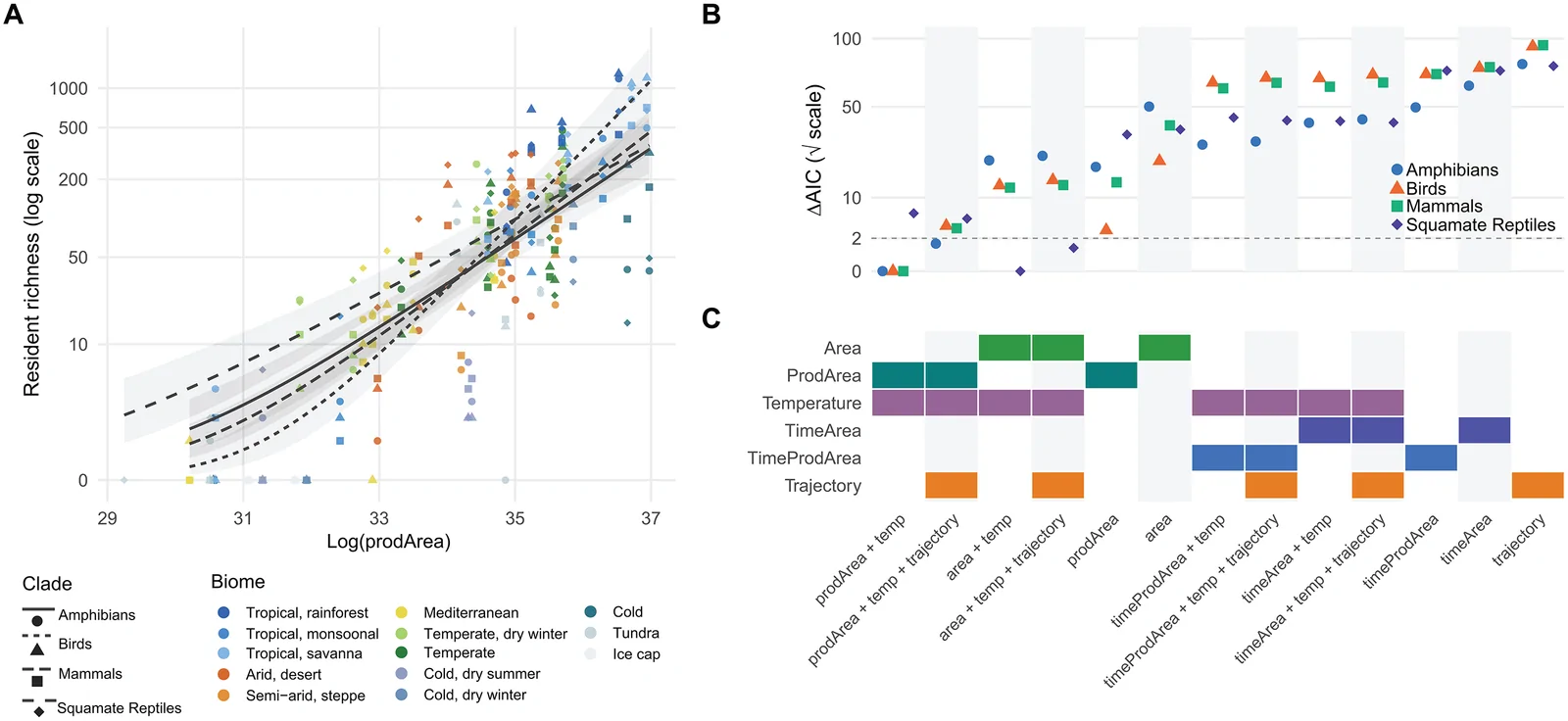
Terrestrial vertebrate resident species richness model selection and best model fit. **(A)** The relationship between resident richness and area-integrated productivity (prodArea) for each of four vertebrate clades from generalised linear mixed effects models which included area-integrated productivity (prodArea) and temperature (temp) as predictors but not time-integrated area (timeArea), time-and-area-integrated productivity (timeAreaProd), or the direction of biome area change through time (trajectory). **(B)** Model fit with ΔAIC for each of four clades of terrestrial vertebrates. Values below two (dashed line) highlight the candidate set of best models for each clade. Values are displayed on square-root scale. **(C)** Explanatory variables used in set of 13 models. The data and code needed to generate this figure can be found at https://doi.org/10.5281/zenodo.17595795.

### Validation of climate-model derived predictions of biome area

To assess whether the model results changed when measuring time-integrated area predictors from climate models, rather than from proxy information, we compared the predictive power of (i) present-day area, (ii) timeArea derived from climate models, and (iii) timeArea derived from the expert-drawn, proxy-based Köppen-biome reconstructions [21]. We performed this validation on realm-biome units of the primary Köppen category which was available both for proxy-based and climate model-based reconstructions (Table F in S1 Appendix). We found that present-day area was a consistently better fit than proxy-based timeArea for all vertebrate classes and richness metrics, and climate model-based estimates of timeArea were a better fit than proxy-based estimates. This result also highlighted that the relationship was robust to the resolution of Köppen category. Finally, to test the robustness of our results to choice of historical biome area measures, and following [38], we also assessed whether the historical minimum biome area provided a better fit than present-day area. Again, present-day area provided a better fit than minimum area in 11 out of 12 comparisons but was a comparable fit (ΔAIC < 2) to minimum area for one comparison (endemic richness of amphibians with the HadCM3L-B model; Table G in S1 Appendix).

## Discussion

### Reconstructing Cenozoic paleobiome change

Our results paint a complex picture of the history of Earth’s biomes through time and across different biogeographic realms. We show that tropical biomes are currently, and have indeed historically been, larger than temperate and polar biomes. However, their relationship to arid biomes is model-dependent: CESM and PlaSim models recover a larger area of tropical biomes throughout the Cenozoic, but HadCM3L models recover a greater area of arid biomes (Fig 1; Fig A–E in S1 Appendix). Despite this disagreement, all five models support a mid-Eocene peak in tropical biome area (~40 Ma) of ~41.8 million km^2^, substantially larger than today’s 32.7 million km^2^ [44]. Continental, humid biomes (Df) analogous to the climatic conditions supporting today’s boreal and mixed broadleaf and coniferous forests, were historically larger than tropical biomes in all but one climate model (CESM) and estimated to be formerly widespread across the Antarctic continent. This is supported by fossil evidence of forests floristically and structurally similar to present-day subpolar forests of Patagonia [45,46].

Realm-specific trajectories often followed global trends. Mirroring the global trend of reduction of rainforest biome area, Afrotropical tropical biomes (Af, Aw, Am) contracted through the Cenozoic, with all five models estimating today’s tropical area below its Eocene–Oligocene peak. This is consistent with progressive late-Cenozoic aridification of the African continent [47]. Yet, in some cases realm-specific trajectories diverge from global trends as a result of more local tectonic, geomorphological, and climatological processes. For example, the Australasian realm has a greater area of tropical biomes in the present day than any earlier point in the Cenozoic, driven by Sahul’s northward drift to tropical latitudes [48], the increase in terrestrial area of Wallacean islands, including Sulawesi [49], the uplift of New Guinea [50], and the expansion of the tropical savannas in northern Australia [51,52].

In some cases, we found conflicting evidence among climate models for a particular trajectory (Fig 2). For example, in the Neotropics, the realm where our climate models disagree most strongly, we found the HadCM3L-A and HadCM3L-B predicted expansion of tropical rainforest, while CESM, PlaSim, and HadCM3L-C predict contraction. The uncertainty across our models is driven by uncertainty in the distribution of arid and semi-arid climates in the region: evaporite proxies support extensive subtropical aridification through the Cenozoic [26], yet 12 published reconstructions of Eocene South American biomes [53] span the full spectrum from continent-wide rainforest to extensive aridity. While there is increasing consensus for contracting tropical biomes through the Neogene with global cooling [54–56], our reconstructions mirror the uncertainty in reconstructions derived from different data and models, as was found for the reconstruction of montane biomes from Pleistocene climate models in a recent study [57]. However, despite some regional uncertainties, overall biome estimates from climate-model reconstructions are largely consistent with each other, and with expert-drawn proxy-based reconstructions (Fig G in S1 Appendix). Given the recent expansion of studies using paleoclimate and paleobiome models in ecological and evolutionary studies [58–61], our results suggest future studies should recognize variation among reconstructions and where possible compare results from multiple sources.

### Equilibrium dynamics and species richness in biomes

Models of species richness showed that variables representing present-day area and energy were better predictors than historical measures across vertebrate classes, climate models, species pools, and time intervals considered. This was true when using climate-model derived estimates of paleobiomes and when using proxy-based estimates of paleobiomes from the lithological and fossil record, so this result is unlikely to be a peculiarity of the climate models themselves. This finding supports a previously reported role for contemporary climate, rather than historical climate, in explaining the richness of resident endotherms [7], but suggests a stronger role for present-day climate than was estimated for resident ectotherms, and for endemic endotherms and ectotherms, albeit based on a different paleobiome definition. We found that including the trajectory of biome area change (expanding/contracting/no change) often either improved model fit, or was a comparably good fit, compared with present-day measures alone. Together, these results suggest that even when historical climate is reconstructed at high resolution, present-day conditions remain the dominant explanation for richness, with biome history contributing a detectable secondary signal that accounts for some residual variation.

A stronger relationship between species richness and present-day rather than historical variables, and only a weak relationship with biome trajectory, suggests that biodiversity tracks changes in the area and energy of biomes as they rise and fall through time. This in turn would suggest that diversity often sits close to the “carrying capacity” of a biome, as would be expected if equilibrium dynamics prevail. Under the equilibrium theory of biodiversity dynamics (ETBD), biodiversity patterns emerge from diversity-dependent diversification, where diversification rates decline as standing diversity approaches a resource-determined carrying capacity set by area and available energy [8,33,62,63]. When the resources of a region are under-utilised, due to recent mass extinction or increases in incoming environmental energy, population sizes may increase, which should reduce extinction rates and may also increase speciation rates (though this pathway is less well understood). On the other hand, as diversity of species in a system increases, population sizes of any individual species may decrease due to partitioning of finite resources, decreasing rates of speciation and increasing rates of extinction. The full pathway predicted under the ETBD is difficult to test because population sizes and resource partitioning are difficult to estimate in large regions. Nonetheless, both our study and another recent study on vertebrate diversity addressed with different data and methods [64] show several lines of evidence that support the ETBD: First, our results in this study, in addition to many other global surveys, show a consistent positive relationship between environmental energy and species diversity across taxa at large spatial scales, often explaining 80%–90% of the variation in broad-scale richness patterns [37,65,66]. Second, species richness shows highly variable taxon-specific responses to alternative non-equilibrium predictors such as the length of time a region has been occupied (the time-for-speciation effect; [67,68]) or rates of diversification [69–72]. This inconsistency would not be expected if these processes, rather than equilibrium dynamics, were the dominant drivers of richness. Finally, at broad spatial and taxonomic scales, diversification rates are negatively correlated with measures of environmental energy and positively correlated with latitude [28,64,73,74]. This is consistent with high-energy tropical regions sitting near carrying capacity while higher-latitude assemblages remain further from equilibrium, as proposed by A. R. Wallace over a century ago [75].

The latter two points are not contradictions of the equilibrium model but predictions of it. If biodiversity is governed by equilibrium principles, any change in resources should lead to a response in biodiversity, and while that change is occurring diversity would be in disequilibrium [62]. For example, the response of biodiversity to environmental change can be fast enough that biomes track equilibrium and show a strong environmental signature, or slow enough that we see evidence of diversity moving toward equilibrium and signs of historical inertia [62]. For example, high latitude regions tend to show higher diversification rates, despite having lower diversity than tropical latitudes [28,73,74] and one explanation for this is that late Cenozoic expansion of cold and polar biomes, combined with the slower life history of cold-adapted organisms, has produced a longer lag between the change in resource availability and diversity, with diversification still catching up to equilibrium. Such lags can be detected in the fossil record, for example in the slow recovery of diversity following a mass extinction [34], and characterising them will help calibrate how quickly diversity can respond to major environmental change [62].

A secondary role for the trajectory of biome area change may be explained by lagged responses to environmental change and reflect historical legacies that shape current species richness in contracting biomes. In squamates, the trajectory coefficient is consistently negative, highlighting that contracting biomes are associated with higher richness than expanding biomes. This trend matches the expectation of the tropical conservatism hypothesis, in which contracting tropical rainforest regions have greater diversity than expected due to their former size [30]. However, rather than showing greater than expected diversity in mesic tropical biomes, the biome-realm-level residuals show that diversity is higher than expected in contracting dry or seasonally dry regions such as arid and semi-arid biomes in the Neotropics (La Costa Xeric Shrublands and Atacama Desert) and Palearctic (Eurasian Steppe), the summer-dry temperate biome in the Palearctic (Mediterranean Basin), and the tropical monsoonal biome in Indomalaya (Deccan Plateau, Lesser Sundas, Indochina). This association could be driven by the ability of squamates to persist in harsh environments [76,77] even as these biomes have contracted through time. This same tolerance for harsh, low-productivity environments helps explain why squamates are the only group for which area outperforms area-integrated productivity as a predictor of richness, reflecting their disproportionate diversity in large but unproductive biomes such as the Australian arid zone and the Sahara [78,79]. It may also reflect that productivity may not be the most appropriate surrogate for resource availability for reptiles in arid environments which, are often abundant in food resources of ants and termites, despite low productivity [80].

In contrast, the trajectory coefficient tended to be positive in endotherms and amphibians, particularly for endemic richness, suggesting that expanding biomes hold more diversity from in situ radiations than contracting biomes after accounting for biome area and energy. Hotspots of residual diversity were concentrated in expanding mesic temperate biomes, particularly the subtropical biomes of the Neotropics (Atlantic Forests), Indomalaya (Gangetic Plains and Yunnan Plateau), and Australasia (Eastern Australia Temperate Forests). These expanding biomes may have higher diversity due to rapid radiations in newly formed environments, particularly those adjacent to existing tropical diversity centres. For example, the Atlantic Forests of Brazil have extremely high endemism and the strong historical geographic and phylogenetic connection to Amazonia [81,82] may have disproportionately facilitated high biodiversity through in situ radiations seeded from adjacent species pools. More broadly, the trajectory effects we recover need not imply ecological carrying capacities, and it is also possible that a short lag-time response of diversity to changes in biome area could produce the same signal (Fine 2015). In such instances we might additionally expect biome area and trajectory to be related to diversification rates, and this may be tested further using area-based diversification models.

### Conclusions

We find that present-day area and energy of global biomes better predict species diversity than historical biome area and energy. This finding is consistent with a model of biodiversity in which diversity relatively rapidly tracks changes in resource availability through time. There was only weak evidence that formerly large biomes retain higher biodiversity than expected given their present-day area and energy, suggesting historical signals of biome dynamics in current diversity are less evident than previously believed. Where a secondary historical signal does appear, it varies by taxon and likely reflects multiple idiosyncratic processes. Our results suggest that present-day species diversity alone is not sufficient to detect the imprint of historical processes on biodiversity. Instead, exploring how diversification rates rapidly respond to biome area dynamics might enable us to understand the temporal effect of past climate change on the evolution of biodiversity. Dynamic eco-evolutionary models of diversification, together with the fossil record, could help reveal historical legacies in present-day diversity patterns, for example in changes in diversification rates [59] or spatial phylogenetic diversity patterns [83].

## Materials and methods

### Climatologies

We obtained five alternative climatologies to derive Köppen biomes [84]. The climatologies were derived from climate models which differed in the fundamental physical processes, parameterisation, and boundary conditions used, and cover a range of commonly used approaches in past, present, and future climatology (Table 1).

**Table 1:** Summary of five alternative climate models for the Cenozoic, including the model name, temporal resolution of time steps, pre- and post-downscaling spatial resolution, model type, and source of the paleo digital elevation model (paleoDEM).

Model Name	Temporal resolution	Spatial resolution	Downscaled resolution	Climate Model	PaleoDEM
HadCM3L-A	5 Ma	2.5° × 3.75°	0.1° × 0.1°	HadCM3BL-M2.1aD	[85]
HadCM3L-B	5 Ma	2.5° × 3.75°	0.1° × 0.1°	HadCM3BL-M2.1aD	[85]
HadCM3L-C	Geological Stages	2.5° × 3.75°	0.5° × 0.5°	HadCM3BL-M2.1aD	[86,87]
CESM	10 Ma	0.9° × 1.25°	0.1° × 0.1°	CESM1.2.2	[85]
PlaSim	5 Ma	5.6° × 5.6°	0.1° × 0.1°	PlaSim-v16.0	[85]

The HadCM3L models are similar to those described in detail in [40] and use the “HadCM3BL-M2.1aD” version of the UK Met Office Hadley Centre model [39], which is a coupled atmosphere–ocean–vegetation model. Compared to the simulations presented in [40], the HadCM3L simulations used in this paper have undergone a number of developments, associated with improvements to the model itself, and to the experimental design. The most significant of these are summarised in the supplementary materials. In HadCM3L-A, the atmospheric CO_2_ was based on CO_2_ observations, derived from [88]. In HadCM3L-B, the atmospheric CO_2_ was modified in such a way that the model-predicted global mean surface temperatures were consistent with those of Scotese and colleagues (2021). HadCM3L-C had the same experimental set-up as HadCM3L-A. However, unlike HadCM3L-A which used the paleotopography of [85], HadCM3L-C used the paleotopographies of [86,87], produced by Getech plc. The fourth climate model was the Community Earth System Model version 1.2.2 (CESM1.2.2) simulation described in [89]. The CESM model is a coupled climate model that consists of atmosphere, ocean, land, sea-ice and river components, at a higher spatial resolution than the HadCM3 models, but coarser temporal resolution (Table 1). Finally, the fifth climate model is an implementation of the Planet Simulator, PlaSim [90,91], that couples the Portable University Model of the Atmosphere (PUMA) to a constant-depth slab ocean and a dynamic sea-ice module, while considering no vegetation dynamics and a constant surface albedo on land.

### Climate downscaling

Downscaling bridges the gap between coarse-resolution climatologies from climate models and high-resolution orographies, based on physical relationships between elevation, wind, temperature, and precipitation. Here, we applied a variation of the CHELSA V.2.1 model adapted to paleoclimatic conditions [92–95] to downscale mean monthly temperature and precipitation values for each of the five climate models using a high resolution paleotopography from [85] at 0.1 x 0.1 degrees for HadCM3L-A, HadCM3L-B, CESM, and PlaSim models, and a high resolution paleotopography (39, 40) for HadCM3L-C at 0.5 x 0.5 degrees.

We downscaled terrestrial surface temperature from the coarse-resolution grid to the high-resolution grid using linear lapse rates of temperature with elevation, accounting for changes in both through time. We estimated spatially variable, free tropospheric lapse rates for each month in each sample year of the HadCM3L and PlaSim climate models from model atmospheric layers. CESM, on the other hand, did not contain atmospheric variables and therefore, we applied a constant lapse rate of 6.5°C per 1,000m globally. To downscale precipitation, we corrected for wind effect, valley exposition, and boundary height layer, which all interact with elevation to shape different patterns of precipitation on leeward versus windward topographies and at different atmospheric layers [93]. Further details on downscaling is found in the S1 Appendix and [93].

### Paleobiome reconstructions

The Köppen climate classification separates climate into five climate categories; tropical (A), arid (B), subtropical/temperate (C), cold/continental (D) and polar (E). These are further subdivided based on the amount *or* seasonal distribution of precipitation, for example, aseasonal tropical (Af) differs from tropical savanna with a dry winter (Aw) or dry summer (As). Arid, temperate, and cold climates also have a third letter indicating the degree or seasonal variation in temperature. For example, a temperate biome with aseasonal precipitation (Cf) might have hot summer (Cfa), warm summer (Cfb) or cold summer (Cfc). We estimated Köppen biomes from downscaled monthly temperature and precipitation values following [84]. To look at areal changes in biomes through time, we calculated the area of each Köppen biome for each climate model at each timestep globally, as well as separately in each biogeographic realm. Biogeographic realms contain largely endemic biotas and are useful partitions for biodiversity data. To reconstruct the location of biogeographic realms through time, we reconstructed the paleo-latitude and paleo-longitude of grid cells within each biogeographic realm with the *reconstruct* function in the “rgplates” R package [96]. For the present day, we used high-resolution Köppen biomes [44], reprojecting them to the downscaled climate model resolution.

We validated climate model-derived reconstructions using three approaches. First, we compared our model-derived Köppen paleobiome reconstructions with expert-drawn, model-free Köppen climate maps based on the lithological and paleobotanical proxy compilations (refs). These maps included the five broad climate categories (A–E). We aggregated our reconstructions to this broad classification level and compared the Köppen category assigned to each cell, at each shared 5 Myr time step from 5–65 Ma. Agreement was quantified using Cohen’s κ and interpreted following Landis and Koch [41]. Second, we compared climate model predictions at 1,382 individual lithological proxy localities. Lithologies were assigned to expected climate zones following Scotese and colleagues [21]: laterites, bauxites, lateritic manganese, and mangroves as tropical; kaolinites, crocodilians, and palms as tropical to warm temperate; evaporites and calcretes as arid; tillites, dropstones, and glendonites as polar; and coals as consistent with tropical, warm temperate, or boreal conditions. For each locality, we rotated the given latitude and longitude to its relevant paleocoordinates at 5 Ma time intervals within each localities specified range, for example, the proxies assigned to the Eocene were rotated to 55, 50, 45, 40, and 35 Ma. We then extracted the model-predicted Köppen type and scored it as consistent or inconsistent with the expected zone across any included time slices. Third, we compared the statistical relationships between species diversity and model-derived biome area histories or model-free proxy-based biome area histories (see Statistical analysis below).

For each Köppen biome in each biogeographic realm, we used a model selection approach to estimate whether changes in biome area showed any strong directional trends: expanding, contracting, or no trend through time. Biomes may have changed at different rates, and to account for this, we modeled a range of relationships between time and area: (i) a linear relationship modeled using standard linear regression; (ii) a quadratic relationship modelled using standard linear regression that includes both linear and squared time terms (waning-waxing or waxing-waning); (iii) an exponential relationship modelled using non-linear least squares approach. We compared these to a null model of no directional trend in area change by fitting (iv) an intercept-only linear model. Each model was fitted to every realm–Köppen combination. Biome-realm trajectories were classified as directional (growth or decline) only when the best-fit model showed a ΔAIC > 2 relative to the null model and the relevant coefficient was statistically significant (*p* < 0.05). Combinations failing these criteria were classified as ‘no significant trend’. We quantified agreement among climate models with pairwise Pearson correlations of biome area trajectories (interpolated to a common 5 Ma grid) and of the derived time-integrated predictor variables (see Statistical analysis below) across all five climate models.

### Present-day biodiversity data

We obtained matching geographic distribution and phylogenetic position data for extant species of terrestrial vertebrates through the VertLife project (https://vertlife.org/) in association with Map of Life (https://mol.org/). Distribution data for birds came from [97], and for squamates, they came from the Global Assessment of Reptile Distributions [98]. For mammals and amphibians, we used modified distributions from the International Union for Conservation of Nature (IUCN) (https://www.iucnredlist.org/) following [47]. In total, we analysed the diversity of 6,898 amphibian species, 8,389 squamate reptiles, 9,330 birds, and 5,515 mammals. We overlaid the distribution of species with the present-day distribution of Köppen biomes to estimate the number of species found in each realm-biome combination. Following Jetz and Fine (2012), we quantified compositional overlap among realm–biome units as the Jaccard similarity of their species, calculated for each unit as the median similarity against all other units and summarised as the median of those values across units (Table H in S1 Appendix).

### Statistical analysis

To test the relationship between species diversity, area, and energy, both historical and current, we fitted a series of linear mixed effects models. Following Jetz and Fine (2012), we calculated three diversity metrics: total (all species per realm-Köppen combination), resident (species assigned to the biome they occupy most), and endemic (species entirely restricted to a realm-biome). For resident richness, in rare cases where species occurred in multiple biomes at equally high prevalence, we randomly selected a single biome. We used the two-letter Köppen classification, which retains high endemicity while distinguishing key biome types and avoids counting species multiple times across realm-biome combinations.

For each realm-biome-climate model combination, in addition to the area-trajectory, we estimated a further five predictor variables: present-day area (area), area-integrated productivity (prodArea), time-integrated area (timeArea), time-and-area-integrated productivity (timeProdArea), and mean temperature (temp). Present-day area was calculated from Beck and colleagues (2018). Net primary productivity (NPP) was measured using the National Center for Ecological Analysis and Synthesis model [99] and mean annual temperature and mean annual precipitation variables from CHELSA v2.0 [93] for the present-day and from our downscaled paleoclimate reconstructions for the past. NPP per grid cell was multiplied by cell area to obtain total productivity. Time-integrated metrics were calculated by summing either area or NPP across all climate model time-steps within three alternative time windows. These three windows account for different periods that might be relevant to the origins of species diversity: whole Cenozoic (65-0 Ma), from the Eocene-Oligocene Boundary, coinciding with major global cooling (35-0 Ma), or from the mid-Miocene, coinciding with major global aridification (15-0 Ma). We include all climate model time steps within each window but exclude the present-day to reduce non-independence with present-day metrics. Temperature was calculated for the present only from CHELSA v2.0 [93], as biomes are defined by temperature ranges.

To select the best-fitting of three historical time windows and five climate models to use as predictor variables of the three diversity metrics, we first fitted negative binomial GLMM with the glmmTMB package in R [100]. We included biogeographic realm as a random effect, and either timeArea or timeProdArea as fixed effects. We fitted models for each of the four vertebrate classes separately, to avoid pseudoreplication from shared predictor values across classes, resulting in 360 models. The best-fitting combination of climate model and time window for timeArea and timeProdArea for each diversity metric and vertebrate class, determined by AIC, were carried forward to downstream analyses. Next, to test the relative contribution of historical and present-day variables on diversity metrics in each vertebrate class, we fitted 13 candidate GLMMs for each combination of vertebrate class and richness metric with area, prodArea, timeArea, timeProdArea, temp, and area trajectory as fixed effects (13 × 4 classes × 3 metrics = 156 fitted models; Fig 3B). These 13 structures were not an exhaustive set of all predictor combinations, but a set of competing hypotheses. Further, nested predictors were not included in the same model, for example, area and prodArea are nested and colinear (*r* = 0.94–0.95). Model fit was compared using AIC, treating ΔAIC < 2 as equivalent support.

To ask whether using climate model-based estimates of paleobiome distributions instead of proxy-derived reconstructions affected our results, we estimated time-integrated area from the proxy-based reconstruction of major paleobiomes [21]. As these reconstructions assign only the five primary Köppen classes (A–E), we aggregated our species distribution data and climate model data to the same broad classification level (A–E), and re-estimated our response variables (total, resident, and endemic richness) and time-integrated-area predictor variables at this resolution. We compared the fit of present-day area with proxy-based and model-based time-integrated area. Finally, to ask whether the minimum area that each biome occupied over the Cenozoic might be a better predictor than the time-integrated area, we extracted the minimum biome area across all time steps for each realm-biome-climate model combination and compared the fit of minimum area with present-day area and time-integrated area.

## Supporting information

S1 Appendix Supplementary text, tables and figures. (DOCX)

## Abbreviations

ETBD: equilibrium theory of biodiversity dynamics
GLMM: generalised linear mixed-effects models
IUCN: International Union for Conservation of Nature
NPP: net primary productivity
PUMA: Portable University Model of the Atmosphere
