# Supplementary material for: High-resolution paleobiome reconstructions highlight ecological and historical dynamics underpinning global terrestrial vertebrate diversity
Source: PLoS Biol. 2026 Oct 23;24(10):e3004077. doi: 10.1371/journal.pbio.3004077 (PMC13719045; doi:10.1371/journal.pbio.3004077)
Supplement: Supplementary file 1 [file pbio.3004077.s001.docx]

# Supporting Information for “High-resolution paleobiome reconstructions highlight ecological dynamics underpinning global terrestrial vertebrate diversity”

Alexander Skeels, Julian Rogger, Marcel Cardillo, Alexander Farnsworth, Daniel J. Lunt, Dirk N. Karger

## S1 Appendix.

#### **Text A**. HadCM3 climate model

Compared to the simulations in Valdes et al (2021), the version of the HadCM3 climate model used in this paper has an update that includes modification to Cloud Condensation Nuclei density and cloud droplet effective radius (Ren *et al.* 2025), following the work of (Sagoo *et al.* 2013) and (Kiehl & Shields 2013). This update increases higher latitude temperatures without significantly changing tropical temperatures, thereby reducing the pole-to-equator temperature gradient, and better aligning higher latitude temperatures with proxy observations. This update has been verified to be effective under a range of climate conditions, including hot, cool, and icehouse conditions, as well as under pre-industrial boundary conditions. As a result, it is suitable for application throughout the Cenozoic. In addition, other internal model parameters were tuned, following (Irvine *et al.* 2013). The ocean barotropic flow solver requires all islands in the paleogeography to be manually defined, to allow non-zero flow through ocean gateways. Although Antarctica was defined as an island in the (Valdes *et al.* 2021) post-Eocene simulations, no other islands were defined. For the simulations in this paper, all islands were defined through the Cenozoic.  A smoothing function was applied in the atmosphere and ocean to avoid gridpoint noise that develops slowly in very long simulations.  In the Valdes et al simulations, the bare-soil albedo is a uniform constant, whereas in these simulations the bare-soil albedo is calculated as a function of the soil carbon content. In (Valdes *et al.* 2021), the atmospheric CO_2_ is consistent with that of (Foster *et al.* 2017), whereas in this study, the atmospheric CO_2_ was modified to be consistent with the CO_2_ record of (Rae *et al.* 2021) for HadCM3-A. Whereas, in HadCM3-B the atmospheric CO_2_ was modified in such a way that the model-predicted global mean surface temperatures were consistent with those of Scotese et al (2021). HadCM3-C had the same experimental set-up as HadCM3-A but with a different paleogeography. Using the naming convention from the Providing Unified Model Access (PUMA) code archive, HadCM3-A is "tfke", HadCM3-B is "tfks", and HadCM3-C used 19 simulations on the GeTech paleogeography spanning the Danian to the Pleistocene (0–66 Ma), taking for each stage the experiment designated current best: "tfgS"/"tfgs" where available and an equivalent earlier-generation run otherwise (tdezc1 for the Pleistocene, tfhrp for the Zanclean, tflzR–tflzY for the Lutetian–Langhian).

*Downscaling temperature and precipitation fields*

We downscaled terrestrial surface temperature from the coarse resolution grid to the high-resolution grid using linear lapse rates of temperature with elevation, accounting for changes in both through time. We estimated spatially variable, free tropospheric lapse rates for each month in each sample year from the pressure-level temperature and geopotential-height fields of the HadCM3L and PlaSim climate models. Variable lapse rates range between ~2.5-7.5°C per 1,000m and showed a generally negative relationship with latitude. CESM, on the other hand, did not contain atmospheric temperature variables and therefore, for that model we applied a constant lapse rate of 6.5°C per 1,000m globally for each of the sampled time points. To do this, we interpolated temperature to sea level the lapse rate and then interpolated these sea level temperatures to the high-resolution grid using cubic-spline interpolation. Finally, we projected back on the elevational surface of the high resolution topography using the equation:

$t=\Gamma d*elev+t0$	**Eq. 1** (Karger *et al.* 2017)

where $t$ is the temperature at a given elevation, $\Gamma d$ equals the lapse rate, $elev$ is the elevation at high resolution and $t0$ equals the interpolated temperature at sea level.

Downscaling precipitation is less straight forward than temperature, due to a non-linear effect of elevation on precipitation. Here, we corrected for the wind effect and valley exposition, as well as the boundary height layer, which all interact with elevation to shape different patterns of precipitation on leeward versus windward topographies and at different atmospheric layers (Karger *et al.* 2017). First, we accounted for the wind effect by estimating wind direction from the meridional and zonal components of wind from the GCMs at coarse resolution, after interpolating these to the high resolution topography using cubic-spline interpolation and projecting to a Mercator coordinate reference system (" + init=EPSG:3857"). Wind effect (H) was estimated from wind direction and the high resolution topography using the morphometric terrain analysis module in SAGA GIS v8.1.4 implemented in in R with the package RSAGA v1.4.2 (Brenning 2008) with a search distance of 300km to account for long-distance leeward precipitation effects (for example, dry valleys in high elevation mountain ranges). Orographic effects on precipitation are strongest where airflow interacts with terrain.

To account for this atmospheric effect, we approximated the lifted condensation level (LCL) using air temperature (T) and dew point temperature (T_D_) from the climate models using the Espy Equation (Espy 1836).

$LCL=125(T-T_{D})$ **Eq. 2**

Where dew point wasn’t available in the climate model output we estimated it from temperature and relative humidity (RH), following (Lawrence 2005).

$T_{D}=ln\left( \frac{RH}{100} \right)+\frac{bT}{c+T}$ **Eq.3**

where b = 17.625 and c = 243.04. For CESM, neither dew point temperature nor relative humidity was available to estimate LCL. Instead, here we estimated the planetary boundary height layer (PBLH) from seven available climatic variables (temperature, precipitation, elevation, zonal and meridional winds, albedo, and surface geopotential) from those simulations. Specifically, we took present-day PBLH from (Kalmus *et al.* 2022) and fitted a multiple regression of present-day estimates of PBLH with the seven predictor variables. This model explained over 80% of the variation in PBLH. We then used the historical variables at each time step to predict the PBLH. Using LCL or PBLH, we interpolated these to the high-resolution grid, and estimated the distance to the relevant atmospheric layer, $d$. We found the maximum value of *d* for each set of grid cells that fell within a single coarse-resolution grid cell, $dmax$_,_ and applied a correction to the wind effect variable:

$Hc=H/(1-(\vee d\vee-dmax)/c))$	**Eq. 4**

Where $c$ is a constant of 9,000 m. Finally, these corrections were applied to precipitation, $P$, using the following equation:

$P=H/{Hc}$	**Eq. 5**

## Supplementary Tables

**Table A.** Agreement between lithological and fossil proxy localities and the predicted biome type across five climate models (HadCM3L-A, HadCM3L-B, HadCM3L-C, CESM, and PlaSim) and a hand-drawn, proxy-based reconstruction (Scotese).

| **Model** | **Climate zone** | **N proxies** | **% consistent** | **% of Scotese ceiling** |
| --- | --- | --- | --- | --- |
| Scotese | Overall | 1315 | 81.4 |  |
| Scotese | Tropical | 133 | 29.3 |  |
| Scotese | Tropical-Warm temperate | 155 | 94.2 |  |
| Scotese | Arid | 361 | 68.7 |  |
| Scotese | Polar/cold | 22 | 22.7 |  |
| Scotese | Wet (coals) | 644 | 98.3 |  |
| HadCM3L-A | Overall | 1315 | 75 | 92.1 |
| HadCM3L-A | Tropical | 133 | 31.6 | 107.8 |
| HadCM3L-A | Tropical-Warm temperate | 155 | 76.1 | 80.8 |
| HadCM3L-A | Arid | 361 | 59 | 85.9 |
| HadCM3L-A | Polar/cold | 22 | 22.7 | 100 |
| HadCM3L-A | Wet (coals) | 644 | 94.4 | 96 |
| HadCM3L-B | Overall | 1315 | 73.3 | 90 |
| HadCM3L-B | Tropical | 133 | 31.6 | 107.8 |
| HadCM3L-B | Tropical-Warm temperate | 155 | 69.7 | 74 |
| HadCM3L-B | Arid | 361 | 54 | 78.6 |
| HadCM3L-B | Polar/cold | 22 | 36.4 | 160.4 |
| HadCM3L-B | Wet (coals) | 644 | 94.9 | 96.5 |
| HadCM3L-C | Overall | 1197 | 72.8 | 89.4 |
| HadCM3L-C | Tropical | 120 | 40.8 | 139.2 |
| HadCM3L-C | Tropical-Warm temperate | 149 | 74.5 | 79.1 |
| HadCM3L-C | Arid | 316 | 42.4 | 61.7 |
| HadCM3L-C | Polar/cold | 20 | 35 | 154.2 |
| HadCM3L-C | Wet (coals) | 592 | 96.5 | 98.2 |
| CESM | Overall | 1301 | 64.2 | 78.9 |
| CESM | Tropical | 131 | 31.3 | 106.8 |
| CESM | Tropical-Warm temperate | 154 | 62.3 | 66.1 |
| CESM | Arid | 356 | 36.8 | 53.6 |
| CESM | Polar/cold | 22 | 59.1 | 260.4 |
| CESM | Wet (coals) | 638 | 86.8 | 88.3 |
| PlaSim | Overall | 1315 | 72.6 | 89.2 |
| PlaSim | Tropical | 133 | 41.4 | 141.3 |
| PlaSim | Tropical-Warm temperate | 155 | 76.1 | 80.8 |
| PlaSim | Arid | 361 | 44.6 | 64.9 |
| PlaSim | Polar/cold | 22 | 45.5 | 200.4 |
| PlaSim | Wet (coals) | 644 | 94.9 | 96.5 |

**Table B.** Mean pairwise correlation coefficient (Pearson’s r) of biome-realm area through time across five alternative climate models.

| **GCM** | *HadCM3L-A* | *HadCM3L-B* | *HadCM3L-C* | *CESM* | *PlaSim* |
| --- | --- | --- | --- | --- | --- |
| *HadCM3L-A* | 1 |  |  |  |  |
| *HadCM3L-B* | 0.856 | 1 |  |  |  |
| *HadCM3L-C* | 0.205 | 0.178 | 1 |  |  |
| *CESM* | 0.431 | 0.417 | 0.061 | 1 |  |
| *PlaSim* | 0.51 | 0.479 | 0.208 | 0.528 | 1 |

**Table C.** Pairwise correlation coefficients (Pearson’s r) of the predictor variables time-integrated area (timeArea) and time-and-area-integrated productivity (timeProdArea) at different time-intervals across five climate models.

| **Comparison** | **GCM** | **HadCM3L-A** | **HadCM3L-B** | **HadCM3L-C** | **CESM** | **PlaSim** |
| --- | --- | --- | --- | --- | --- | --- |
| *timeArea, 65Ma-0Ma* | HadCM3L-A | 1 | 0.997 | 0.963 | 0.934 | 0.938 |
|  | HadCM3L-B | 0.997 | 1 | 0.975 | 0.938 | 0.944 |
|  | HadCM3L-C | 0.963 | 0.975 | 1 | 0.92 | 0.955 |
|  | CESM | 0.934 | 0.938 | 0.92 | 1 | 0.898 |
|  | PlaSim | 0.938 | 0.944 | 0.955 | 0.898 | 1 |
| *timeArea, 35Ma-0Ma* | HadCM3L-A | 1 | 0.994 | 0.974 | 0.848 | 0.967 |
|  | HadCM3L-B | 0.994 | 1 | 0.985 | 0.844 | 0.967 |
|  | HadCM3L-C | 0.974 | 0.985 | 1 | 0.842 | 0.961 |
|  | CESM | 0.848 | 0.844 | 0.842 | 1 | 0.814 |
|  | PlaSim | 0.967 | 0.967 | 0.961 | 0.814 | 1 |
| *timeArea, 15Ma-0Ma* | HadCM3L-A | 1 | 0.992 | 0.983 | 0.826 | 0.963 |
|  | HadCM3L-B | 0.992 | 1 | 0.991 | 0.822 | 0.955 |
|  | HadCM3L-C | 0.983 | 0.991 | 1 | 0.838 | 0.954 |
|  | CESM | 0.826 | 0.822 | 0.838 | 1 | 0.805 |
|  | PlaSim | 0.963 | 0.955 | 0.954 | 0.805 | 1 |
| *timeProdArea, 65Ma-0Ma* | HadCM3L-A | 1 | 0.997 | 0.958 | 0.954 | 0.865 |
|  | HadCM3L-B | 0.997 | 1 | 0.971 | 0.953 | 0.874 |
|  | HadCM3L-C | 0.958 | 0.971 | 1 | 0.925 | 0.927 |
|  | CESM | 0.954 | 0.953 | 0.925 | 1 | 0.864 |
|  | PlaSim | 0.865 | 0.874 | 0.927 | 0.864 | 1 |
| *timeProdArea, 35Ma-0Ma* | HadCM3L-A | 1 | 0.992 | 0.977 | 0.888 | 0.92 |
|  | HadCM3L-B | 0.992 | 1 | 0.984 | 0.871 | 0.923 |
|  | HadCM3L-C | 0.977 | 0.984 | 1 | 0.865 | 0.923 |
|  | CESM | 0.888 | 0.871 | 0.865 | 1 | 0.775 |
|  | PlaSim | 0.92 | 0.923 | 0.923 | 0.775 | 1 |
| *timeProdArea, 15Ma-0Ma* | HadCM3L-A | 1 | 0.987 | 0.985 | 0.847 | 0.913 |
|  | HadCM3L-B | 0.987 | 1 | 0.984 | 0.826 | 0.901 |
|  | HadCM3L-C | 0.985 | 0.984 | 1 | 0.84 | 0.906 |
|  | CESM | 0.847 | 0.826 | 0.84 | 1 | 0.724 |
|  | PlaSim | 0.913 | 0.901 | 0.906 | 0.724 | 1 |

**Table D.** Model selection and explained variance (Nagelkerke pseudo-R^2^) estimates of two time-integrated measures of area and energy (timeArea and timeProdArea) as predictors of three measures of species diversity (total, resident, and endemic) for five different climate models (HadCM3L-A, HadCm3L-B, HadCM3L-C, CESM, PlaSim), three time windows (15-0Ma, 35-0Ma, 65-0Ma), and four vertebrate classes (amphibians, reptiles, birds, and mammals). AIC = Akaike Information Criteria. Cnd R^2^ is the conditional pseudo-R^2^ value including fixed and random effects. Mrg. R^2^ is the marginal pseudo-R^2^ of the fixed effects. Best fitting models (ΔAIC < 2) are highlighted in bold.

| Climate Model | Window | AIC | ΔAIC | Cond. R² | Marg. R² |
| --- | --- | --- | --- | --- | --- |
| **Amphibians — Total richness — timeArea** | | | | | |
| **HadCM3L-A** | **35 Ma** | **728.23** | **0.00** | **0.559** | **0.241** |
| **HadCM3L-A** | **15 Ma** | **729.11** | **0.88** | **0.539** | **0.231** |
| **HadCM3L-A** | **65 Ma** | **729.38** | **1.15** | **0.545** | **0.223** |
| HadCM3L-B | 15 Ma | 730.34 | 2.11 | 0.517 | 0.214 |
| HadCM3L-B | 65 Ma | 730.61 | 2.38 | 0.523 | 0.201 |
| HadCM3L-B | 35 Ma | 730.79 | 2.56 | 0.520 | 0.201 |
| HadCM3L-C | 65 Ma | 732.03 | 3.80 | 0.476 | 0.173 |
| HadCM3L-C | 35 Ma | 732.54 | 4.31 | 0.510 | 0.170 |
| HadCM3L-C | 15 Ma | 733.70 | 5.47 | 0.498 | 0.151 |
| CESM | 65 Ma | 736.87 | 8.64 | 0.385 | 0.062 |
| CESM | 35 Ma | 737.22 | 8.99 | 0.382 | 0.057 |
| CESM | 15 Ma | 737.36 | 9.13 | 0.382 | 0.054 |
| PlaSim | 65 Ma | 737.52 | 9.29 | 0.376 | 0.034 |
| PlaSim | 35 Ma | 737.84 | 9.61 | 0.374 | 0.030 |
| PlaSim | 15 Ma | 737.86 | 9.63 | 0.373 | 0.031 |
| **Amphibians — Total richness — timeProdArea** | | | | | |
| **HadCM3L-A** | **35 Ma** | **718.36** | **0.00** | **0.612** | **0.352** |
| **HadCM3L-A** | **15 Ma** | **718.77** | **0.41** | **0.593** | **0.346** |
| **HadCM3L-B** | **15 Ma** | **718.88** | **0.52** | **0.584** | **0.361** |
| **HadCM3L-B** | **35 Ma** | **719.97** | **1.61** | **0.593** | **0.350** |
| HadCM3L-A | 65 Ma | 720.43 | 2.07 | 0.604 | 0.342 |
| HadCM3L-B | 65 Ma | 721.49 | 3.13 | 0.586 | 0.332 |
| HadCM3L-C | 35 Ma | 721.83 | 3.47 | 0.601 | 0.336 |
| HadCM3L-C | 65 Ma | 722.03 | 3.67 | 0.539 | 0.315 |
| HadCM3L-C | 15 Ma | 722.19 | 3.83 | 0.611 | 0.347 |
| CESM | 65 Ma | 736.29 | 17.93 | 0.384 | 0.071 |
| CESM | 35 Ma | 736.59 | 18.23 | 0.381 | 0.067 |
| CESM | 15 Ma | 736.70 | 18.34 | 0.381 | 0.064 |
| PlaSim | 65 Ma | 736.90 | 18.54 | 0.372 | 0.040 |
| PlaSim | 15 Ma | 737.07 | 18.71 | 0.369 | 0.040 |
| PlaSim | 35 Ma | 737.14 | 18.78 | 0.370 | 0.038 |
| **Amphibians — Resident richness — timeArea** | | | | | |
| **HadCM3L-A** | **15 Ma** | **609.48** | **0.00** | **0.647** | **0.433** |
| **HadCM3L-A** | **35 Ma** | **609.97** | **0.49** | **0.658** | **0.418** |
| HadCM3L-A | 65 Ma | 612.14 | 2.66 | 0.615 | 0.345 |
| HadCM3L-B | 15 Ma | 612.85 | 3.37 | 0.584 | 0.344 |
| HadCM3L-B | 65 Ma | 614.07 | 4.59 | 0.581 | 0.298 |
| HadCM3L-B | 35 Ma | 614.40 | 4.92 | 0.569 | 0.297 |
| HadCM3L-C | 65 Ma | 615.49 | 6.01 | 0.503 | 0.250 |
| HadCM3L-C | 35 Ma | 616.84 | 7.36 | 0.528 | 0.225 |
| HadCM3L-C | 15 Ma | 617.77 | 8.29 | 0.514 | 0.206 |
| PlaSim | 65 Ma | 619.21 | 9.73 | 0.341 | 0.112 |
| CESM | 65 Ma | 619.39 | 9.91 | 0.360 | 0.133 |
| PlaSim | 35 Ma | 619.69 | 10.21 | 0.333 | 0.106 |
| PlaSim | 15 Ma | 619.75 | 10.27 | 0.330 | 0.111 |
| CESM | 35 Ma | 619.78 | 10.30 | 0.351 | 0.127 |
| CESM | 15 Ma | 619.92 | 10.44 | 0.348 | 0.125 |
| **Amphibians — Resident richness — timeProdArea** | | | | | |
| **HadCM3L-A** | **15 Ma** | **595.43** | **0.00** | **0.716** | **0.571** |
| **HadCM3L-A** | **35 Ma** | **597.14** | **1.71** | **0.724** | **0.549** |
| HadCM3L-B | 15 Ma | 599.12 | 3.69 | 0.665 | 0.508 |
| HadCM3L-A | 65 Ma | 601.55 | 6.12 | 0.683 | 0.479 |
| HadCM3L-B | 35 Ma | 601.85 | 6.42 | 0.667 | 0.480 |
| HadCM3L-B | 65 Ma | 603.48 | 8.05 | 0.662 | 0.456 |
| HadCM3L-C | 65 Ma | 604.81 | 9.38 | 0.582 | 0.408 |
| HadCM3L-C | 15 Ma | 605.87 | 10.44 | 0.651 | 0.425 |
| HadCM3L-C | 35 Ma | 605.91 | 10.48 | 0.635 | 0.407 |
| PlaSim | 65 Ma | 618.15 | 22.72 | 0.338 | 0.127 |
| PlaSim | 15 Ma | 618.37 | 22.94 | 0.330 | 0.130 |
| PlaSim | 35 Ma | 618.48 | 23.05 | 0.332 | 0.124 |
| CESM | 65 Ma | 618.65 | 23.22 | 0.360 | 0.145 |
| CESM | 35 Ma | 618.96 | 23.53 | 0.353 | 0.141 |
| CESM | 15 Ma | 619.05 | 23.62 | 0.351 | 0.140 |
| **Amphibians — Endemic richness — timeArea** | | | | | |
| **HadCM3L-A** | **15 Ma** | **511.23** | **0.00** | **0.648** | **0.383** |
| **HadCM3L-A** | **35 Ma** | **511.87** | **0.64** | **0.652** | **0.367** |
| HadCM3L-A | 65 Ma | 513.54 | 2.31 | 0.621 | 0.301 |
| HadCM3L-B | 15 Ma | 513.74 | 2.51 | 0.598 | 0.304 |
| HadCM3L-B | 65 Ma | 515.06 | 3.83 | 0.595 | 0.254 |
| HadCM3L-B | 35 Ma | 515.25 | 4.02 | 0.582 | 0.256 |
| HadCM3L-C | 65 Ma | 516.39 | 5.16 | 0.537 | 0.208 |
| HadCM3L-C | 35 Ma | 517.61 | 6.38 | 0.542 | 0.181 |
| CESM | 65 Ma | 517.72 | 6.49 | 0.437 | 0.174 |
| CESM | 35 Ma | 518.18 | 6.95 | 0.426 | 0.169 |
| HadCM3L-C | 15 Ma | 518.34 | 7.11 | 0.529 | 0.162 |
| CESM | 15 Ma | 518.37 | 7.14 | 0.421 | 0.166 |
| PlaSim | 65 Ma | 519.63 | 8.40 | 0.372 | 0.076 |
| PlaSim | 35 Ma | 520.13 | 8.90 | 0.361 | 0.070 |
| PlaSim | 15 Ma | 520.20 | 8.97 | 0.355 | 0.074 |
| **Amphibians — Endemic richness — timeProdArea** | | | | | |
| **HadCM3L-A** | **15 Ma** | **496.81** | **0.00** | **0.739** | **0.562** |
| HadCM3L-A | 35 Ma | 498.92 | 2.11 | 0.737 | 0.533 |
| HadCM3L-B | 15 Ma | 499.31 | 2.50 | 0.699 | 0.504 |
| HadCM3L-B | 35 Ma | 502.49 | 5.68 | 0.692 | 0.464 |
| HadCM3L-A | 65 Ma | 502.72 | 5.91 | 0.702 | 0.460 |
| HadCM3L-B | 65 Ma | 504.34 | 7.53 | 0.688 | 0.432 |
| HadCM3L-C | 65 Ma | 504.93 | 8.12 | 0.641 | 0.401 |
| HadCM3L-C | 35 Ma | 506.50 | 9.69 | 0.665 | 0.388 |
| HadCM3L-C | 15 Ma | 506.68 | 9.87 | 0.668 | 0.393 |
| CESM | 65 Ma | 516.66 | 19.85 | 0.441 | 0.194 |
| CESM | 35 Ma | 517.02 | 20.21 | 0.431 | 0.191 |
| CESM | 15 Ma | 517.14 | 20.33 | 0.428 | 0.190 |
| PlaSim | 65 Ma | 518.58 | 21.77 | 0.369 | 0.094 |
| PlaSim | 15 Ma | 518.86 | 22.05 | 0.356 | 0.098 |
| PlaSim | 35 Ma | 518.95 | 22.14 | 0.360 | 0.092 |
| **Reptiles — Total richness — timeArea** | | | | | |
| **HadCM3L-A** | **35 Ma** | **774.83** | **0.00** | **0.468** | **0.249** |
| **HadCM3L-A** | **65 Ma** | **775.15** | **0.32** | **0.465** | **0.242** |
| **HadCM3L-A** | **15 Ma** | **776.22** | **1.39** | **0.432** | **0.231** |
| HadCM3L-B | 65 Ma | 777.05 | 2.22 | 0.424 | 0.216 |
| HadCM3L-B | 35 Ma | 777.36 | 2.53 | 0.415 | 0.215 |
| HadCM3L-B | 15 Ma | 778.83 | 4.00 | 0.386 | 0.184 |
| HadCM3L-C | 35 Ma | 779.66 | 4.83 | 0.389 | 0.182 |
| HadCM3L-C | 15 Ma | 779.87 | 5.04 | 0.392 | 0.181 |
| HadCM3L-C | 65 Ma | 780.04 | 5.21 | 0.354 | 0.166 |
| PlaSim | 65 Ma | 785.64 | 10.81 | 0.281 | 0.014 |
| PlaSim | 35 Ma | 785.84 | 11.01 | 0.284 | 0.011 |
| PlaSim | 15 Ma | 785.88 | 11.05 | 0.284 | 0.011 |
| CESM | 65 Ma | 786.37 | 11.54 | 0.301 | 0.004 |
| CESM | 35 Ma | 786.42 | 11.59 | 0.303 | 0.003 |
| CESM | 15 Ma | 786.44 | 11.61 | 0.305 | 0.002 |
| **Reptiles — Total richness — timeProdArea** | | | | | |
| **HadCM3L-A** | **35 Ma** | **772.61** | **0.00** | **0.463** | **0.291** |
| **HadCM3L-A** | **65 Ma** | **773.27** | **0.66** | **0.462** | **0.280** |
| **HadCM3L-A** | **15 Ma** | **774.01** | **1.40** | **0.422** | **0.277** |
| HadCM3L-B | 35 Ma | 774.64 | 2.03 | 0.416 | 0.275 |
| HadCM3L-B | 65 Ma | 774.75 | 2.14 | 0.426 | 0.267 |
| HadCM3L-B | 15 Ma | 776.58 | 3.97 | 0.373 | 0.232 |
| HadCM3L-C | 15 Ma | 777.34 | 4.73 | 0.399 | 0.258 |
| HadCM3L-C | 35 Ma | 777.77 | 5.16 | 0.380 | 0.234 |
| HadCM3L-C | 65 Ma | 778.31 | 5.70 | 0.336 | 0.207 |
| PlaSim | 65 Ma | 785.81 | 13.20 | 0.280 | 0.011 |
| PlaSim | 35 Ma | 785.97 | 13.36 | 0.283 | 0.009 |
| PlaSim | 15 Ma | 786.02 | 13.41 | 0.283 | 0.009 |
| CESM | 65 Ma | 786.36 | 13.75 | 0.301 | 0.004 |
| CESM | 35 Ma | 786.40 | 13.79 | 0.302 | 0.003 |
| CESM | 15 Ma | 786.42 | 13.81 | 0.303 | 0.003 |
| **Reptiles — Resident richness — timeArea** | | | | | |
| **HadCM3L-A** | **35 Ma** | **655.66** | **0.00** | **0.483** | **0.321** |
| **HadCM3L-A** | **15 Ma** | **655.96** | **0.30** | **0.444** | **0.314** |
| **HadCM3L-A** | **65 Ma** | **656.58** | **0.92** | **0.464** | **0.297** |
| HadCM3L-B | 35 Ma | 658.32 | 2.66 | 0.395 | 0.256 |
| HadCM3L-B | 65 Ma | 658.45 | 2.79 | 0.398 | 0.247 |
| HadCM3L-B | 15 Ma | 659.52 | 3.86 | 0.354 | 0.216 |
| HadCM3L-C | 15 Ma | 660.14 | 4.48 | 0.358 | 0.218 |
| HadCM3L-C | 35 Ma | 660.57 | 4.91 | 0.329 | 0.194 |
| HadCM3L-C | 65 Ma | 660.81 | 5.15 | 0.286 | 0.177 |
| PlaSim | 65 Ma | 663.63 | 7.97 | 0.158 | 0.075 |
| PlaSim | 35 Ma | 663.94 | 8.28 | 0.157 | 0.068 |
| PlaSim | 15 Ma | 663.96 | 8.30 | 0.155 | 0.069 |
| CESM | 65 Ma | 666.37 | 10.71 | 0.189 | 0.001 |
| CESM | 35 Ma | 666.39 | 10.73 | 0.190 | 0.001 |
| CESM | 15 Ma | 666.39 | 10.73 | 0.191 | 0.000 |
| **Reptiles — Resident richness — timeProdArea** | | | | | |
| **HadCM3L-A** | **35 Ma** | **655.68** | **0.00** | **0.409** | **0.307** |
| **HadCM3L-A** | **15 Ma** | **655.68** | **0.00** | **0.365** | **0.308** |
| **HadCM3L-A** | **65 Ma** | **656.58** | **0.90** | **0.403** | **0.288** |
| **HadCM3L-B** | **35 Ma** | **657.47** | **1.79** | **0.341** | **0.269** |
| HadCM3L-B | 65 Ma | 657.86 | 2.18 | 0.350 | 0.257 |
| HadCM3L-B | 15 Ma | 658.86 | 3.18 | 0.284 | 0.220 |
| HadCM3L-C | 15 Ma | 658.99 | 3.31 | 0.317 | 0.262 |
| HadCM3L-C | 35 Ma | 659.97 | 4.29 | 0.274 | 0.215 |
| HadCM3L-C | 65 Ma | 660.31 | 4.63 | 0.224 | 0.188 |
| PlaSim | 65 Ma | 663.90 | 8.22 | 0.139 | 0.074 |
| PlaSim | 35 Ma | 664.15 | 8.47 | 0.138 | 0.068 |
| PlaSim | 15 Ma | 664.20 | 8.52 | 0.133 | 0.069 |
| CESM | 65 Ma | 666.38 | 10.70 | 0.189 | 0.001 |
| CESM | 35 Ma | 666.39 | 10.71 | 0.190 | 0.001 |
| CESM | 15 Ma | 666.39 | 10.71 | 0.191 | 0.000 |
| **Reptiles — Endemic richness — timeArea** | | | | | |
| **HadCM3L-A** | **35 Ma** | **561.21** | **0.00** | **0.511** | **0.224** |
| **HadCM3L-A** | **65 Ma** | **561.68** | **0.47** | **0.502** | **0.209** |
| **HadCM3L-A** | **15 Ma** | **561.83** | **0.62** | **0.479** | **0.207** |
| HadCM3L-B | 65 Ma | 563.79 | 2.58 | 0.446 | 0.149 |
| HadCM3L-B | 35 Ma | 563.95 | 2.74 | 0.440 | 0.149 |
| HadCM3L-B | 15 Ma | 564.88 | 3.67 | 0.420 | 0.114 |
| HadCM3L-C | 15 Ma | 565.01 | 3.80 | 0.430 | 0.120 |
| HadCM3L-C | 35 Ma | 565.41 | 4.20 | 0.409 | 0.102 |
| HadCM3L-C | 65 Ma | 565.74 | 4.53 | 0.385 | 0.088 |
| PlaSim | 65 Ma | 567.43 | 6.22 | 0.351 | 0.023 |
| PlaSim | 15 Ma | 567.66 | 6.45 | 0.353 | 0.020 |
| PlaSim | 35 Ma | 567.68 | 6.47 | 0.354 | 0.019 |
| CESM | 15 Ma | 568.51 | 7.30 | 0.418 | 0.003 |
| CESM | 35 Ma | 568.53 | 7.32 | 0.416 | 0.003 |
| CESM | 65 Ma | 568.55 | 7.34 | 0.413 | 0.002 |
| **Reptiles — Endemic richness — timeProdArea** | | | | | |
| **HadCM3L-A** | **35 Ma** | **560.73** | **0.00** | **0.458** | **0.239** |
| **HadCM3L-A** | **15 Ma** | **561.24** | **0.51** | **0.418** | **0.228** |
| **HadCM3L-A** | **65 Ma** | **561.28** | **0.55** | **0.457** | **0.224** |
| HadCM3L-B | 35 Ma | 562.96 | 2.23 | 0.396 | 0.185 |
| HadCM3L-B | 65 Ma | 563.02 | 2.29 | 0.408 | 0.179 |
| HadCM3L-C | 15 Ma | 564.16 | 3.43 | 0.382 | 0.166 |
| HadCM3L-B | 15 Ma | 564.20 | 3.47 | 0.364 | 0.134 |
| HadCM3L-C | 35 Ma | 564.95 | 4.22 | 0.356 | 0.127 |
| HadCM3L-C | 65 Ma | 565.41 | 4.68 | 0.330 | 0.103 |
| PlaSim | 65 Ma | 567.64 | 6.91 | 0.344 | 0.020 |
| PlaSim | 35 Ma | 567.84 | 7.11 | 0.347 | 0.017 |
| PlaSim | 15 Ma | 567.85 | 7.12 | 0.345 | 0.017 |
| CESM | 15 Ma | 568.51 | 7.78 | 0.418 | 0.003 |
| CESM | 35 Ma | 568.53 | 7.80 | 0.417 | 0.002 |
| CESM | 65 Ma | 568.55 | 7.82 | 0.414 | 0.002 |
| **Birds — Total richness — timeArea** | | | | | |
| **HadCM3L-C** | **35 Ma** | **852.87** | **0.00** | **0.594** | **0.223** |
| **HadCM3L-C** | **15 Ma** | **854.53** | **1.66** | **0.582** | **0.206** |
| **HadCM3L-A** | **35 Ma** | **854.82** | **1.95** | **0.561** | **0.167** |
| HadCM3L-C | 65 Ma | 855.15 | 2.28 | 0.544 | 0.188 |
| HadCM3L-A | 65 Ma | 855.21 | 2.34 | 0.559 | 0.172 |
| HadCM3L-A | 15 Ma | 855.79 | 2.92 | 0.549 | 0.158 |
| HadCM3L-B | 15 Ma | 855.84 | 2.97 | 0.543 | 0.169 |
| HadCM3L-B | 35 Ma | 856.09 | 3.22 | 0.544 | 0.166 |
| HadCM3L-B | 65 Ma | 856.26 | 3.39 | 0.543 | 0.163 |
| PlaSim | 65 Ma | 865.39 | 12.52 | 0.450 | 0.025 |
| PlaSim | 15 Ma | 865.58 | 12.71 | 0.448 | 0.024 |
| PlaSim | 35 Ma | 865.63 | 12.76 | 0.449 | 0.023 |
| CESM | 65 Ma | 865.95 | 13.08 | 0.442 | 0.025 |
| CESM | 35 Ma | 866.12 | 13.25 | 0.442 | 0.023 |
| CESM | 15 Ma | 866.20 | 13.33 | 0.444 | 0.022 |
| **Birds — Total richness — timeProdArea** | | | | | |
| **HadCM3L-C** | **35 Ma** | **844.75** | **0.00** | **0.634** | **0.318** |
| **HadCM3L-C** | **15 Ma** | **845.41** | **0.66** | **0.636** | **0.321** |
| HadCM3L-C | 65 Ma | 848.13 | 3.38 | 0.573 | 0.271 |
| HadCM3L-A | 35 Ma | 849.00 | 4.25 | 0.587 | 0.228 |
| HadCM3L-B | 15 Ma | 849.21 | 4.46 | 0.570 | 0.245 |
| HadCM3L-B | 35 Ma | 849.30 | 4.55 | 0.577 | 0.247 |
| HadCM3L-A | 65 Ma | 849.73 | 4.98 | 0.585 | 0.235 |
| HadCM3L-A | 15 Ma | 849.93 | 5.18 | 0.574 | 0.219 |
| HadCM3L-B | 65 Ma | 850.46 | 5.71 | 0.571 | 0.234 |
| PlaSim | 65 Ma | 865.24 | 20.49 | 0.448 | 0.026 |
| PlaSim | 15 Ma | 865.37 | 20.62 | 0.446 | 0.026 |
| PlaSim | 35 Ma | 865.43 | 20.68 | 0.447 | 0.025 |
| CESM | 65 Ma | 865.76 | 21.01 | 0.442 | 0.027 |
| CESM | 35 Ma | 865.92 | 21.17 | 0.442 | 0.025 |
| CESM | 15 Ma | 865.99 | 21.24 | 0.442 | 0.024 |
| **Birds — Resident richness — timeArea** | | | | | |
| **HadCM3L-A** | **15 Ma** | **630.77** | **0.00** | **0.599** | **0.448** |
| **HadCM3L-A** | **35 Ma** | **632.11** | **1.34** | **0.602** | **0.426** |
| HadCM3L-B | 15 Ma | 633.74 | 2.97 | 0.571 | 0.394 |
| HadCM3L-A | 65 Ma | 634.95 | 4.18 | 0.564 | 0.371 |
| HadCM3L-B | 35 Ma | 635.39 | 4.62 | 0.553 | 0.366 |
| HadCM3L-B | 65 Ma | 636.63 | 5.86 | 0.533 | 0.340 |
| HadCM3L-C | 65 Ma | 637.02 | 6.25 | 0.464 | 0.309 |
| HadCM3L-C | 35 Ma | 637.97 | 7.20 | 0.509 | 0.304 |
| HadCM3L-C | 15 Ma | 638.36 | 7.59 | 0.510 | 0.304 |
| PlaSim | 15 Ma | 641.13 | 10.36 | 0.208 | 0.200 |
| PlaSim | 65 Ma | 641.32 | 10.55 | 0.206 | 0.197 |
| PlaSim | 35 Ma | 641.51 | 10.74 | 0.203 | 0.188 |
| CESM | 65 Ma | 644.97 | 14.20 | 0.138 | 0.066 |
| CESM | 15 Ma | 644.97 | 14.20 | 0.136 | 0.066 |
| CESM | 35 Ma | 644.99 | 14.22 | 0.136 | 0.066 |
| **Birds — Resident richness — timeProdArea** | | | | | |
| **HadCM3L-A** | **15 Ma** | **626.04** | **0.00** | **0.585** | **0.496** |
| HadCM3L-A | 35 Ma | 628.12 | 2.08 | 0.591 | 0.468 |
| HadCM3L-B | 15 Ma | 629.68 | 3.64 | 0.531 | 0.426 |
| HadCM3L-B | 35 Ma | 630.65 | 4.61 | 0.552 | 0.436 |
| HadCM3L-A | 65 Ma | 631.49 | 5.45 | 0.557 | 0.414 |
| HadCM3L-B | 65 Ma | 632.66 | 6.62 | 0.538 | 0.407 |
| HadCM3L-C | 15 Ma | 632.97 | 6.93 | 0.545 | 0.422 |
| HadCM3L-C | 65 Ma | 633.36 | 7.32 | 0.439 | 0.360 |
| HadCM3L-C | 35 Ma | 633.82 | 7.78 | 0.508 | 0.384 |
| PlaSim | 15 Ma | 640.72 | 14.68 | 0.202 | 0.202 |
| PlaSim | 65 Ma | 640.95 | 14.91 | 0.200 | 0.200 |
| PlaSim | 35 Ma | 641.08 | 15.04 | 0.195 | 0.195 |
| CESM | 65 Ma | 644.85 | 18.81 | 0.131 | 0.068 |
| CESM | 15 Ma | 644.85 | 18.81 | 0.128 | 0.068 |
| CESM | 35 Ma | 644.87 | 18.83 | 0.129 | 0.068 |
| **Birds — Endemic richness — timeArea** | | | | | |
| **PlaSim** | **15 Ma** | **344.41** | **0.00** | **0.879** | **0.723** |
| PlaSim | 65 Ma | 346.42 | 2.01 | 0.844 | 0.636 |
| PlaSim | 35 Ma | 346.85 | 2.44 | 0.849 | 0.652 |
| HadCM3L-A | 15 Ma | 349.44 | 5.03 | 0.619 | 0.228 |
| HadCM3L-A | 35 Ma | 349.87 | 5.46 | 0.617 | 0.212 |
| HadCM3L-A | 65 Ma | 351.15 | 6.74 | 0.589 | 0.154 |
| HadCM3L-B | 15 Ma | 351.38 | 6.97 | 0.574 | 0.151 |
| HadCM3L-B | 35 Ma | 352.19 | 7.78 | 0.562 | 0.118 |
| HadCM3L-B | 65 Ma | 352.29 | 7.88 | 0.568 | 0.112 |
| HadCM3L-C | 65 Ma | 352.79 | 8.38 | 0.548 | 0.084 |
| HadCM3L-C | 15 Ma | 353.05 | 8.64 | 0.554 | 0.082 |
| HadCM3L-C | 35 Ma | 353.12 | 8.71 | 0.545 | 0.075 |
| CESM | 65 Ma | 354.67 | 10.26 | 0.459 | 0.028 |
| CESM | 35 Ma | 354.69 | 10.28 | 0.458 | 0.027 |
| CESM | 15 Ma | 354.72 | 10.31 | 0.457 | 0.026 |
| **Birds — Endemic richness — timeProdArea** | | | | | |
| **PlaSim** | **15 Ma** | **338.11** | **0.00** | **0.921** | **0.847** |
| PlaSim | 35 Ma | 341.82 | 3.71 | 0.897 | 0.796 |
| PlaSim | 65 Ma | 341.96 | 3.85 | 0.890 | 0.778 |
| HadCM3L-A | 15 Ma | 343.71 | 5.60 | 0.653 | 0.382 |
| HadCM3L-A | 35 Ma | 344.92 | 6.81 | 0.645 | 0.349 |
| HadCM3L-B | 15 Ma | 345.91 | 7.80 | 0.602 | 0.309 |
| HadCM3L-A | 65 Ma | 347.13 | 9.02 | 0.613 | 0.274 |
| HadCM3L-B | 35 Ma | 347.49 | 9.38 | 0.594 | 0.270 |
| HadCM3L-B | 65 Ma | 348.55 | 10.44 | 0.592 | 0.232 |
| HadCM3L-C | 15 Ma | 348.63 | 10.52 | 0.581 | 0.237 |
| HadCM3L-C | 65 Ma | 349.07 | 10.96 | 0.560 | 0.199 |
| HadCM3L-C | 35 Ma | 349.48 | 11.37 | 0.559 | 0.201 |
| CESM | 65 Ma | 354.42 | 16.31 | 0.447 | 0.038 |
| CESM | 35 Ma | 354.44 | 16.33 | 0.446 | 0.038 |
| CESM | 15 Ma | 354.45 | 16.34 | 0.444 | 0.038 |
| **Mammals — Total richness — timeArea** | | | | | |
| **HadCM3L-A** | **35 Ma** | **771.47** | **0.00** | **0.511** | **0.273** |
| **HadCM3L-A** | **65 Ma** | **771.99** | **0.52** | **0.505** | **0.278** |
| **HadCM3L-A** | **15 Ma** | **773.23** | **1.76** | **0.487** | **0.254** |
| HadCM3L-B | 65 Ma | 774.06 | 2.59 | 0.472 | 0.257 |
| HadCM3L-C | 35 Ma | 774.30 | 2.83 | 0.483 | 0.270 |
| HadCM3L-B | 35 Ma | 774.41 | 2.94 | 0.471 | 0.253 |
| HadCM3L-B | 15 Ma | 774.89 | 3.42 | 0.460 | 0.247 |
| HadCM3L-C | 15 Ma | 775.35 | 3.88 | 0.478 | 0.262 |
| HadCM3L-C | 65 Ma | 776.57 | 5.10 | 0.422 | 0.227 |
| PlaSim | 65 Ma | 785.57 | 14.10 | 0.333 | 0.045 |
| PlaSim | 15 Ma | 785.67 | 14.20 | 0.333 | 0.045 |
| PlaSim | 35 Ma | 785.90 | 14.43 | 0.332 | 0.041 |
| CESM | 65 Ma | 787.14 | 15.67 | 0.315 | 0.032 |
| CESM | 35 Ma | 787.33 | 15.86 | 0.316 | 0.029 |
| CESM | 15 Ma | 787.39 | 15.92 | 0.318 | 0.028 |
| **Mammals — Total richness — timeProdArea** | | | | | |
| **HadCM3L-A** | **35 Ma** | **765.27** | **0.00** | **0.548** | **0.341** |
| **HadCM3L-A** | **65 Ma** | **766.25** | **0.98** | **0.543** | **0.347** |
| **HadCM3L-A** | **15 Ma** | **767.18** | **1.91** | **0.522** | **0.321** |
| HadCM3L-C | 15 Ma | 767.57 | 2.30 | 0.542 | 0.370 |
| HadCM3L-B | 35 Ma | 767.65 | 2.38 | 0.518 | 0.338 |
| HadCM3L-C | 35 Ma | 767.83 | 2.56 | 0.527 | 0.354 |
| HadCM3L-B | 65 Ma | 768.14 | 2.87 | 0.514 | 0.334 |
| HadCM3L-B | 15 Ma | 768.65 | 3.38 | 0.497 | 0.322 |
| HadCM3L-C | 65 Ma | 771.10 | 5.83 | 0.454 | 0.297 |
| PlaSim | 65 Ma | 785.51 | 20.24 | 0.333 | 0.045 |
| PlaSim | 15 Ma | 785.58 | 20.31 | 0.332 | 0.045 |
| PlaSim | 35 Ma | 785.79 | 20.52 | 0.332 | 0.042 |
| CESM | 65 Ma | 787.03 | 21.76 | 0.316 | 0.033 |
| CESM | 35 Ma | 787.20 | 21.93 | 0.316 | 0.030 |
| CESM | 15 Ma | 787.25 | 21.98 | 0.318 | 0.029 |
| **Mammals — Resident richness — timeArea** | | | | | |
| **HadCM3L-A** | **15 Ma** | **603.50** | **0.00** | **0.500** | **0.495** |
| **HadCM3L-A** | **35 Ma** | **604.59** | **1.09** | **0.507** | **0.471** |
| HadCM3L-B | 15 Ma | 607.41 | 3.91 | 0.383 | 0.382 |
| HadCM3L-A | 65 Ma | 607.89 | 4.39 | 0.404 | 0.368 |
| HadCM3L-B | 35 Ma | 608.78 | 5.28 | 0.366 | 0.346 |
| HadCM3L-B | 65 Ma | 609.57 | 6.07 | 0.349 | 0.324 |
| HadCM3L-C | 65 Ma | 610.98 | 7.48 | 0.252 | 0.252 |
| HadCM3L-C | 35 Ma | 611.52 | 8.02 | 0.284 | 0.263 |
| HadCM3L-C | 15 Ma | 611.72 | 8.22 | 0.302 | 0.273 |
| PlaSim | 15 Ma | 614.77 | 11.27 | 0.151 | 0.151 |
| PlaSim | 65 Ma | 614.83 | 11.33 | 0.150 | 0.150 |
| PlaSim | 35 Ma | 615.08 | 11.58 | 0.144 | 0.144 |
| CESM | 65 Ma | 617.79 | 14.29 | 0.075 | 0.075 |
| CESM | 15 Ma | 617.83 | 14.33 | 0.075 | 0.075 |
| CESM | 35 Ma | 617.84 | 14.34 | 0.074 | 0.074 |
| **Mammals — Resident richness — timeProdArea** | | | | | |
| **HadCM3L-A** | **15 Ma** | **598.29** | **0.00** | **0.507** | **0.507** |
| **HadCM3L-A** | **35 Ma** | **599.75** | **1.46** | **0.502** | **0.502** |
| HadCM3L-B | 15 Ma | 602.69 | 4.40 | 0.399 | 0.399 |
| HadCM3L-B | 35 Ma | 603.62 | 5.33 | 0.404 | 0.404 |
| HadCM3L-A | 65 Ma | 603.69 | 5.40 | 0.418 | 0.418 |
| HadCM3L-B | 65 Ma | 605.11 | 6.82 | 0.387 | 0.387 |
| HadCM3L-C | 15 Ma | 606.39 | 8.10 | 0.366 | 0.366 |
| HadCM3L-C | 35 Ma | 607.17 | 8.88 | 0.332 | 0.332 |
| HadCM3L-C | 65 Ma | 607.51 | 9.22 | 0.283 | 0.283 |
| PlaSim | 15 Ma | 614.59 | 16.30 | 0.148 | 0.148 |
| PlaSim | 65 Ma | 614.67 | 16.38 | 0.147 | 0.147 |
| PlaSim | 35 Ma | 614.86 | 16.57 | 0.143 | 0.143 |
| CESM | 65 Ma | 617.68 | 19.39 | 0.077 | 0.077 |
| CESM | 15 Ma | 617.72 | 19.43 | 0.076 | 0.076 |
| CESM | 35 Ma | 617.73 | 19.44 | 0.076 | 0.076 |
| **Mammals — Endemic richness — timeArea** | | | | | |
| **HadCM3L-A** | **15 Ma** | **421.24** | **0.00** | **0.449** | **0.292** |
| **HadCM3L-A** | **35 Ma** | **421.47** | **0.23** | **0.462** | **0.284** |
| HadCM3L-A | 65 Ma | 423.40 | 2.16 | 0.393 | 0.207 |
| HadCM3L-B | 15 Ma | 423.44 | 2.20 | 0.376 | 0.202 |
| HadCM3L-B | 35 Ma | 424.35 | 3.11 | 0.356 | 0.170 |
| HadCM3L-B | 65 Ma | 424.84 | 3.60 | 0.343 | 0.154 |
| HadCM3L-C | 15 Ma | 425.75 | 4.51 | 0.330 | 0.124 |
| HadCM3L-C | 35 Ma | 426.03 | 4.79 | 0.304 | 0.107 |
| PlaSim | 15 Ma | 426.32 | 5.08 | 0.253 | 0.093 |
| PlaSim | 65 Ma | 426.50 | 5.26 | 0.253 | 0.086 |
| HadCM3L-C | 65 Ma | 426.54 | 5.30 | 0.269 | 0.084 |
| PlaSim | 35 Ma | 426.71 | 5.47 | 0.250 | 0.080 |
| CESM | 65 Ma | 428.67 | 7.43 | 0.205 | 0.014 |
| CESM | 35 Ma | 428.69 | 7.45 | 0.206 | 0.013 |
| CESM | 15 Ma | 428.69 | 7.45 | 0.206 | 0.013 |
| **Mammals — Endemic richness — timeProdArea** | | | | | |
| **HadCM3L-A** | **15 Ma** | **415.29** | **0.00** | **0.433** | **0.433** |
| **HadCM3L-A** | **35 Ma** | **416.26** | **0.97** | **0.469** | **0.402** |
| HadCM3L-B | 15 Ma | 417.90 | 2.61 | 0.342 | 0.342 |
| HadCM3L-A | 65 Ma | 419.04 | 3.75 | 0.409 | 0.325 |
| HadCM3L-B | 35 Ma | 419.28 | 3.99 | 0.361 | 0.311 |
| HadCM3L-B | 65 Ma | 420.71 | 5.42 | 0.355 | 0.277 |
| HadCM3L-C | 15 Ma | 420.76 | 5.47 | 0.337 | 0.291 |
| HadCM3L-C | 35 Ma | 421.90 | 6.61 | 0.288 | 0.250 |
| HadCM3L-C | 65 Ma | 422.77 | 7.48 | 0.215 | 0.215 |
| PlaSim | 15 Ma | 425.58 | 10.29 | 0.220 | 0.127 |
| PlaSim | 65 Ma | 425.88 | 10.59 | 0.227 | 0.113 |
| PlaSim | 35 Ma | 426.01 | 10.72 | 0.222 | 0.109 |
| CESM | 65 Ma | 428.49 | 13.20 | 0.185 | 0.022 |
| CESM | 15 Ma | 428.50 | 13.21 | 0.184 | 0.022 |
| CESM | 35 Ma | 428.51 | 13.22 | 0.185 | 0.021 |

**Table E** Model selection and explained variance (Nagelkerke pseudo-R^2^) estimates from 13 models of species diversity estimated with three measures: total, resident, and endemic. temp=temperature; area=present-day area; prodArea = present-day area-integrated productivity; timeArea = time-integrated area from the relevant best fitting model of timeArea (Table S4); timeProdArea = time-and-area-integrated productivity from the relevant best fitting model of timeProdArea (Table S4), traj = the trajectory estimated for the biome based which climate-model was selected as the relevant best fitting in timeArea. Traj had three levels: growth, decline, or no trend. Cond R^2^ is the conditional pseudo-R^2^ value including fixed and random effects. Mrg. R^2^ is the marginal pseudo-R^2^ of the fixed effects. AIC = Akaike Information Criteria. Best fitting models (ΔAIC < 2) are highlighted in bold.

| Model | AIC | ΔAIC | Cond. R² | Marg. R² |
| --- | --- | --- | --- | --- |
| **prodArea+temp** | **677.10** | **0.00** | **0.817** | **0.740** |
| **prodArea+temp+traj** | **678.70** | **1.60** | **0.825** | **0.741** |
| prodArea | 694.00 | 16.90 | 0.743 | 0.597 |
| area+temp | 696.60 | 19.50 | 0.759 | 0.666 |
| area+temp+traj | 698.90 | 21.80 | 0.766 | 0.668 |
| timeProdArea+temp | 704.00 | 26.90 | 0.718 | 0.578 |
| timeProdArea+temp+traj | 706.50 | 29.40 | 0.726 | 0.583 |
| timeArea+temp | 711.70 | 34.60 | 0.686 | 0.537 |
| timeArea+temp+traj | 714.60 | 37.40 | 0.693 | 0.542 |
| area | 717.80 | 40.70 | 0.632 | 0.406 |
| timeProdArea | 718.40 | 41.20 | 0.612 | 0.352 |
| timeArea | 728.20 | 51.10 | 0.559 | 0.241 |
| trajectory | 741.60 | 64.40 | 0.389 | 0.004 |
| **prodArea+temp** | **545.80** | **0.00** | **0.880** | **0.853** |
| **prodArea+temp+traj** | **547.20** | **1.40** | **0.882** | **0.859** |
| prodArea | 566.00 | 20.10 | 0.829 | 0.761 |
| area+temp | 568.60 | 22.70 | 0.837 | 0.810 |
| area+temp+traj | 570.40 | 24.60 | 0.839 | 0.817 |
| timeProdArea+temp | 575.50 | 29.60 | 0.809 | 0.751 |
| timeProdArea+temp+traj | 576.90 | 31.10 | 0.823 | 0.771 |
| timeArea+temp | 586.50 | 40.70 | 0.777 | 0.719 |
| timeArea+temp+traj | 588.40 | 42.60 | 0.790 | 0.740 |
| timeProdArea | 595.40 | 49.60 | 0.716 | 0.571 |
| area | 596.00 | 50.10 | 0.730 | 0.622 |
| timeArea | 609.50 | 63.60 | 0.647 | 0.433 |
| trajectory | 625.10 | 79.30 | 0.326 | 0.011 |
| **prodArea+temp** | **465.00** | **0.00** | **0.843** | **0.792** |
| prodArea+temp+traj | 467.60 | 2.60 | 0.841 | 0.800 |
| timeProdArea+temp | 478.70 | 13.70 | 0.808 | 0.736 |
| area+temp | 479.50 | 14.50 | 0.813 | 0.761 |
| timeProdArea+temp+traj | 480.20 | 15.20 | 0.815 | 0.762 |
| area+temp+traj | 482.00 | 17.00 | 0.810 | 0.772 |
| prodArea | 482.70 | 17.70 | 0.772 | 0.652 |
| timeArea+temp | 488.10 | 23.10 | 0.781 | 0.705 |
| timeArea+temp+traj | 490.20 | 25.20 | 0.784 | 0.730 |
| timeProdArea | 496.80 | 31.80 | 0.739 | 0.562 |
| area | 504.50 | 39.50 | 0.676 | 0.504 |
| timeArea | 511.20 | 46.20 | 0.648 | 0.383 |
| trajectory | 524.30 | 59.30 | 0.387 | 0.008 |
| **prodArea+temp+traj** | **713.50** | **0.00** | **0.832** | **0.776** |
| area+temp+traj | 717.10 | 3.60 | 0.820 | 0.745 |
| prodArea+temp | 718.00 | 4.60 | 0.839 | 0.780 |
| area+temp | 721.60 | 8.10 | 0.827 | 0.753 |
| timeProdArea+temp+traj | 736.20 | 22.70 | 0.762 | 0.677 |
| timeArea+temp+traj | 737.20 | 23.70 | 0.754 | 0.667 |
| timeProdArea+temp | 741.50 | 28.00 | 0.768 | 0.686 |
| timeArea+temp | 742.70 | 29.20 | 0.759 | 0.675 |
| prodArea | 749.10 | 35.60 | 0.692 | 0.557 |
| area | 757.00 | 43.50 | 0.668 | 0.453 |
| timeProdArea | 772.60 | 59.10 | 0.463 | 0.291 |
| trajectory | 773.90 | 60.50 | 0.378 | 0.035 |
| timeArea | 774.80 | 61.40 | 0.468 | 0.249 |
| **area+temp** | **581.40** | **0.00** | **0.896** | **0.859** |
| **area+temp+traj** | **582.40** | **1.00** | **0.889** | **0.852** |
| prodArea+temp+traj | 586.50 | 5.10 | 0.883 | 0.859 |
| prodArea+temp | 587.60 | 6.20 | 0.886 | 0.859 |
| prodArea | 615.90 | 34.50 | 0.812 | 0.763 |
| area | 618.50 | 37.10 | 0.830 | 0.688 |
| timeArea+temp+traj | 622.20 | 40.80 | 0.766 | 0.735 |
| timeArea+temp | 623.00 | 41.60 | 0.776 | 0.740 |
| timeProdArea+temp+traj | 623.40 | 42.00 | 0.763 | 0.732 |
| timeProdArea+temp | 625.00 | 43.60 | 0.769 | 0.732 |
| timeArea | 655.70 | 74.30 | 0.483 | 0.321 |
| timeProdArea | 655.70 | 74.30 | 0.409 | 0.307 |
| trajectory | 659.20 | 77.80 | 0.368 | 0.080 |
| **area+temp** | **499.80** | **0.00** | **0.869** | **0.789** |
| **area+temp+traj** | **500.70** | **0.80** | **0.860** | **0.783** |
| **prodArea+temp+traj** | **501.60** | **1.80** | **0.861** | **0.815** |
| prodArea+temp | 502.30 | 2.40 | 0.864 | 0.808 |
| prodArea | 527.00 | 27.10 | 0.796 | 0.681 |
| timeArea+temp+traj | 528.90 | 29.10 | 0.764 | 0.714 |
| timeProdArea+temp+traj | 529.30 | 29.50 | 0.763 | 0.715 |
| timeArea+temp | 529.90 | 30.10 | 0.767 | 0.706 |
| area | 530.00 | 30.10 | 0.817 | 0.581 |
| timeProdArea+temp | 530.70 | 30.90 | 0.763 | 0.703 |
| timeProdArea | 560.70 | 60.90 | 0.458 | 0.239 |
| timeArea | 561.20 | 61.40 | 0.511 | 0.224 |
| trajectory | 563.10 | 63.20 | 0.477 | 0.044 |
| **prodArea+temp+traj** | **822.30** | **0.00** | **0.764** | **0.559** |
| **timeProdArea+temp+traj** | **823.00** | **0.70** | **0.802** | **0.593** |
| prodArea+temp | 824.40 | 2.10 | 0.727 | 0.501 |
| timeProdArea+temp | 829.00 | 6.70 | 0.755 | 0.536 |
| prodArea | 829.40 | 7.10 | 0.700 | 0.421 |
| timeArea+temp+traj | 832.90 | 10.50 | 0.759 | 0.539 |
| area+temp+traj | 835.00 | 12.70 | 0.710 | 0.475 |
| area+temp | 835.40 | 13.10 | 0.677 | 0.416 |
| timeArea+temp | 835.80 | 13.40 | 0.723 | 0.490 |
| area | 843.40 | 21.10 | 0.643 | 0.292 |
| timeProdArea | 844.70 | 22.40 | 0.634 | 0.318 |
| timeArea | 852.90 | 30.50 | 0.594 | 0.223 |
| trajectory | 868.20 | 45.90 | 0.475 | 0.019 |
| **prodArea+temp** | **554.30** | **0.00** | **0.923** | **0.892** |
| prodArea | 557.40 | 3.10 | 0.919 | 0.873 |
| prodArea+temp+traj | 558.10 | 3.80 | 0.923 | 0.894 |
| area+temp | 568.00 | 13.60 | 0.910 | 0.858 |
| area+temp+traj | 569.60 | 15.30 | 0.911 | 0.873 |
| area | 576.70 | 22.40 | 0.907 | 0.794 |
| timeProdArea+temp | 620.00 | 65.70 | 0.646 | 0.625 |
| timeArea+temp | 623.10 | 68.80 | 0.645 | 0.618 |
| timeProdArea+temp+traj | 623.50 | 69.10 | 0.645 | 0.634 |
| timeArea+temp+traj | 625.70 | 71.40 | 0.649 | 0.636 |
| timeProdArea | 626.00 | 71.70 | 0.585 | 0.496 |
| timeArea | 630.80 | 76.40 | 0.599 | 0.448 |
| trajectory | 647.70 | 93.40 | 0.165 | 0.044 |
| **prodArea+temp** | **323.00** | **0.00** | **0.811** | **0.704** |
| **prodArea** | **324.10** | **1.10** | **0.819** | **0.633** |
| prodArea+temp+traj | 325.80 | 2.80 | 0.817 | 0.690 |
| area+temp | 331.70 | 8.70 | 0.775 | 0.634 |
| timeProdArea+temp | 332.40 | 9.50 | 0.903 | 0.864 |
| timeProdArea+temp+traj | 333.30 | 10.30 | 0.925 | 0.887 |
| area+temp+traj | 334.10 | 11.10 | 0.794 | 0.622 |
| area | 335.70 | 12.80 | 0.807 | 0.493 |
| timeArea+temp | 336.40 | 13.40 | 0.861 | 0.801 |
| timeArea+temp+traj | 336.80 | 13.90 | 0.903 | 0.844 |
| timeProdArea | 338.10 | 15.10 | 0.921 | 0.847 |
| timeArea | 344.40 | 21.40 | 0.879 | 0.723 |
| trajectory | 355.90 | 32.90 | 0.491 | 0.042 |
| **prodArea+temp** | **726.60** | **0.00** | **0.792** | **0.673** |
| prodArea+temp+traj | 730.40 | 3.70 | 0.796 | 0.664 |
| prodArea | 736.40 | 9.70 | 0.737 | 0.602 |
| area+temp | 740.40 | 13.70 | 0.736 | 0.597 |
| area+temp+traj | 743.70 | 17.10 | 0.744 | 0.583 |
| area | 753.90 | 27.30 | 0.659 | 0.460 |
| timeProdArea+temp | 756.50 | 29.90 | 0.656 | 0.463 |
| timeProdArea+temp+traj | 760.30 | 33.70 | 0.664 | 0.457 |
| timeArea+temp | 761.40 | 34.80 | 0.625 | 0.425 |
| timeArea+temp+traj | 765.00 | 38.40 | 0.635 | 0.418 |
| timeProdArea | 765.30 | 38.70 | 0.548 | 0.341 |
| timeArea | 771.50 | 44.90 | 0.511 | 0.273 |
| trajectory | 790.30 | 63.70 | 0.326 | 0.013 |
| **prodArea+temp** | **526.50** | **0.00** | **0.903** | **0.903** |
| prodArea+temp+traj | 529.80 | 3.40 | 0.904 | 0.904 |
| area+temp | 539.30 | 12.90 | 0.884 | 0.884 |
| area+temp+traj | 540.10 | 13.70 | 0.890 | 0.890 |
| prodArea | 541.10 | 14.60 | 0.875 | 0.871 |
| area | 565.80 | 39.30 | 0.840 | 0.788 |
| timeProdArea+temp | 588.30 | 61.80 | 0.645 | 0.645 |
| timeArea+temp | 589.40 | 62.90 | 0.668 | 0.668 |
| timeProdArea+temp+traj | 592.10 | 65.60 | 0.648 | 0.648 |
| timeArea+temp+traj | 592.30 | 65.80 | 0.684 | 0.684 |
| timeProdArea | 598.30 | 71.80 | 0.507 | 0.507 |
| timeArea | 603.50 | 77.00 | 0.500 | 0.495 |
| trajectory | 620.80 | 94.40 | 0.041 | 0.041 |
| **prodArea+temp+traj** | **364.50** | **0.00** | **0.861** | **0.861** |
| **prodArea+temp** | **366.00** | **1.50** | **0.850** | **0.850** |
| area+temp+traj | 374.30 | 9.80 | 0.837 | 0.837 |
| area+temp | 377.20 | 12.70 | 0.824 | 0.824 |
| prodArea | 384.90 | 20.40 | 0.798 | 0.734 |
| timeProdArea+temp | 392.60 | 28.10 | 0.708 | 0.708 |
| timeProdArea+temp+traj | 393.00 | 28.50 | 0.728 | 0.728 |
| timeArea+temp+traj | 395.00 | 30.50 | 0.737 | 0.737 |
| timeArea+temp | 395.70 | 31.20 | 0.707 | 0.707 |
| area | 399.90 | 35.40 | 0.780 | 0.583 |
| timeProdArea | 415.30 | 50.80 | 0.433 | 0.433 |
| timeArea | 421.20 | 56.70 | 0.449 | 0.292 |
| trajectory | 430.90 | 66.30 | 0.256 | 0.005 |

**Table F.** Model selection and explained variance (Nagelkerke pseudo-R²) estimates from 19 models of species diversity estimated with three measures (total, resident, and endemic), comparing present-day biome area with time-integrated biome area derived from five different climate models (HadCM3L-A, HadCM3L-B, HadCM3L-C, CESM, PlaSim) and from Scotese et al. proxy-based reconstructions, across three time windows (15-0 Ma, 35-0 Ma, 65-0 Ma), under the broad Köppen-biome classification scheme (A–E). Cnd. R² is the conditional pseudo-R² value including fixed and random effects. Mrg. R² is the marginal pseudo-R² of the fixed effects. AIC = Akaike Information Criteria. Best fitting models (ΔAIC < 2) are highlighted in bold.

| Model | AIC | ΔAIC | Cond. R² | Marg. R² |
| --- | --- | --- | --- | --- |
| **Amphibians — Total richness** | | | | |
| **Present-day area (broad)** | **731.20** | **0.00** | **0.483** | **0.212** |
| **GCM HadCM3L-A area (65 Ma)** | **732.70** | **1.50** | **0.500** | **0.158** |
| GCM HadCM3L-A area (35 Ma) | 733.30 | 2.10 | 0.486 | 0.154 |
| GCM PlaSim area (65 Ma) | 733.40 | 2.20 | 0.469 | 0.135 |
| GCM HadCM3L-B area (65 Ma) | 734.30 | 3.10 | 0.457 | 0.132 |
| GCM PlaSim area (35 Ma) | 734.70 | 3.50 | 0.441 | 0.120 |
| GCM HadCM3L-A area (15 Ma) | 734.90 | 3.70 | 0.448 | 0.116 |
| GCM HadCM3L-B area (35 Ma) | 735.50 | 4.30 | 0.430 | 0.114 |
| GCM PlaSim area (15 Ma) | 736.20 | 5.00 | 0.412 | 0.093 |
| GCM HadCM3L-B area (15 Ma) | 736.30 | 5.10 | 0.407 | 0.096 |
| GCM HadCM3L-C area (35 Ma) | 736.90 | 5.70 | 0.399 | 0.076 |
| GCM HadCM3L-C area (65 Ma) | 736.90 | 5.70 | 0.400 | 0.073 |
| Proxy area (65 Ma) | 737.20 | 6.00 | 0.446 | 0.043 |
| Proxy area (35 Ma) | 737.40 | 6.20 | 0.443 | 0.041 |
| GCM HadCM3L-C area (15 Ma) | 737.40 | 6.20 | 0.392 | 0.067 |
| GCM CESM area (65 Ma) | 737.60 | 6.40 | 0.378 | 0.047 |
| Proxy area (15 Ma) | 737.70 | 6.50 | 0.434 | 0.037 |
| GCM CESM area (35 Ma) | 738.30 | 7.10 | 0.366 | 0.036 |
| GCM CESM area (15 Ma) | 738.80 | 7.60 | 0.357 | 0.024 |
| **Amphibians — Resident richness** | | | | |
| **Present-day area (broad)** | **612.80** | **0.00** | **0.561** | **0.422** |
| GCM HadCM3L-A area (65 Ma) | 616.40 | 3.60 | 0.507 | 0.240 |
| GCM PlaSim area (65 Ma) | 616.60 | 3.80 | 0.481 | 0.212 |
| GCM HadCM3L-A area (35 Ma) | 616.70 | 3.90 | 0.494 | 0.246 |
| GCM PlaSim area (35 Ma) | 617.50 | 4.70 | 0.449 | 0.215 |
| GCM HadCM3L-A area (15 Ma) | 617.70 | 4.90 | 0.451 | 0.212 |
| GCM HadCM3L-B area (65 Ma) | 618.30 | 5.50 | 0.451 | 0.192 |
| GCM PlaSim area (15 Ma) | 618.70 | 5.90 | 0.414 | 0.204 |
| GCM HadCM3L-B area (35 Ma) | 619.40 | 6.60 | 0.413 | 0.174 |
| GCM HadCM3L-B area (15 Ma) | 619.80 | 7.00 | 0.392 | 0.171 |
| Proxy area (65 Ma) | 620.40 | 7.60 | 0.402 | 0.084 |
| GCM HadCM3L-C area (35 Ma) | 620.50 | 7.70 | 0.373 | 0.127 |
| GCM HadCM3L-C area (65 Ma) | 620.50 | 7.70 | 0.367 | 0.118 |
| Proxy area (35 Ma) | 620.60 | 7.80 | 0.394 | 0.080 |
| GCM HadCM3L-C area (15 Ma) | 620.60 | 7.80 | 0.368 | 0.130 |
| GCM CESM area (65 Ma) | 620.60 | 7.80 | 0.329 | 0.098 |
| Proxy area (15 Ma) | 621.00 | 8.20 | 0.380 | 0.075 |
| GCM CESM area (35 Ma) | 621.20 | 8.40 | 0.304 | 0.089 |
| GCM CESM area (15 Ma) | 621.60 | 8.80 | 0.280 | 0.080 |
| **Amphibians — Endemic richness** | | | | |
| **GCM PlaSim area (65 Ma)** | **516.40** | **0.00** | **0.545** | **0.192** |
| **Present-day area (broad)** | **516.50** | **0.10** | **0.511** | **0.294** |
| **GCM HadCM3L-A area (65 Ma)** | **516.50** | **0.10** | **0.547** | **0.217** |
| **GCM HadCM3L-A area (35 Ma)** | **517.00** | **0.60** | **0.528** | **0.218** |
| **GCM HadCM3L-A area (15 Ma)** | **517.80** | **1.40** | **0.496** | **0.193** |
| **GCM PlaSim area (35 Ma)** | **517.80** | **1.40** | **0.495** | **0.181** |
| **GCM HadCM3L-B area (65 Ma)** | **518.20** | **1.80** | **0.509** | **0.173** |
| GCM CESM area (65 Ma) | 518.70 | 2.30 | 0.441 | 0.137 |
| Proxy area (65 Ma) | 518.90 | 2.50 | 0.471 | 0.100 |
| Proxy area (35 Ma) | 519.30 | 2.90 | 0.459 | 0.094 |
| GCM HadCM3L-B area (35 Ma) | 519.30 | 2.90 | 0.468 | 0.151 |
| GCM PlaSim area (15 Ma) | 519.30 | 2.90 | 0.445 | 0.155 |
| GCM CESM area (35 Ma) | 519.60 | 3.20 | 0.400 | 0.125 |
| Proxy area (15 Ma) | 519.70 | 3.30 | 0.440 | 0.088 |
| GCM HadCM3L-B area (15 Ma) | 519.90 | 3.50 | 0.440 | 0.140 |
| GCM HadCM3L-C area (65 Ma) | 519.90 | 3.50 | 0.451 | 0.116 |
| GCM HadCM3L-C area (35 Ma) | 520.00 | 3.60 | 0.449 | 0.118 |
| GCM CESM area (15 Ma) | 520.20 | 3.80 | 0.363 | 0.111 |
| GCM HadCM3L-C area (15 Ma) | 520.40 | 4.00 | 0.436 | 0.113 |
| **Reptiles — Total richness** | | | | |
| **Present-day area (broad)** | **767.80** | **0.00** | **0.595** | **0.351** |
| GCM HadCM3L-A area (65 Ma) | 773.90 | 6.10 | 0.541 | 0.256 |
| GCM HadCM3L-A area (35 Ma) | 774.40 | 6.60 | 0.524 | 0.250 |
| GCM HadCM3L-A area (15 Ma) | 776.20 | 8.40 | 0.483 | 0.228 |
| GCM PlaSim area (65 Ma) | 776.20 | 8.40 | 0.503 | 0.223 |
| GCM HadCM3L-B area (65 Ma) | 776.80 | 9.00 | 0.485 | 0.226 |
| GCM PlaSim area (35 Ma) | 777.10 | 9.30 | 0.479 | 0.214 |
| GCM HadCM3L-B area (35 Ma) | 778.00 | 10.20 | 0.451 | 0.211 |
| GCM PlaSim area (15 Ma) | 778.00 | 10.20 | 0.457 | 0.209 |
| GCM HadCM3L-B area (15 Ma) | 779.00 | 11.20 | 0.426 | 0.198 |
| Proxy area (65 Ma) | 780.40 | 12.60 | 0.460 | 0.116 |
| Proxy area (35 Ma) | 780.70 | 12.90 | 0.455 | 0.112 |
| Proxy area (15 Ma) | 781.40 | 13.60 | 0.441 | 0.105 |
| GCM HadCM3L-C area (15 Ma) | 781.80 | 14.00 | 0.369 | 0.151 |
| GCM HadCM3L-C area (35 Ma) | 782.20 | 14.40 | 0.363 | 0.137 |
| GCM HadCM3L-C area (65 Ma) | 782.30 | 14.50 | 0.361 | 0.129 |
| GCM CESM area (65 Ma) | 785.00 | 17.20 | 0.305 | 0.047 |
| GCM CESM area (35 Ma) | 785.30 | 17.50 | 0.299 | 0.041 |
| GCM CESM area (15 Ma) | 785.40 | 17.60 | 0.288 | 0.039 |
| **Reptiles — Resident richness** | | | | |
| **Present-day area (broad)** | **639.30** | **0.00** | **0.778** | **0.618** |
| GCM HadCM3L-A area (35 Ma) | 654.10 | 14.80 | 0.607 | 0.448 |
| GCM HadCM3L-A area (65 Ma) | 654.50 | 15.20 | 0.607 | 0.427 |
| GCM HadCM3L-A area (15 Ma) | 656.00 | 16.70 | 0.543 | 0.405 |
| GCM PlaSim area (35 Ma) | 656.80 | 17.50 | 0.547 | 0.391 |
| GCM PlaSim area (65 Ma) | 656.90 | 17.60 | 0.551 | 0.375 |
| GCM PlaSim area (15 Ma) | 657.00 | 17.70 | 0.534 | 0.399 |
| GCM HadCM3L-B area (65 Ma) | 658.40 | 19.10 | 0.518 | 0.352 |
| GCM HadCM3L-B area (35 Ma) | 659.30 | 20.00 | 0.479 | 0.341 |
| GCM HadCM3L-B area (15 Ma) | 659.70 | 20.40 | 0.459 | 0.342 |
| Proxy area (65 Ma) | 661.40 | 22.10 | 0.397 | 0.159 |
| Proxy area (35 Ma) | 661.60 | 22.30 | 0.392 | 0.155 |
| Proxy area (15 Ma) | 662.00 | 22.70 | 0.374 | 0.149 |
| GCM HadCM3L-C area (15 Ma) | 662.70 | 23.40 | 0.310 | 0.202 |
| GCM HadCM3L-C area (35 Ma) | 663.40 | 24.10 | 0.283 | 0.162 |
| GCM HadCM3L-C area (65 Ma) | 663.50 | 24.20 | 0.272 | 0.148 |
| GCM CESM area (15 Ma) | 665.10 | 25.80 | 0.177 | 0.072 |
| GCM CESM area (65 Ma) | 665.20 | 25.90 | 0.189 | 0.056 |
| GCM CESM area (35 Ma) | 665.30 | 26.00 | 0.187 | 0.057 |
| **Reptiles — Endemic richness** | | | | |
| **Present-day area (broad)** | **543.60** | **0.00** | **0.799** | **0.531** |
| GCM HadCM3L-A area (65 Ma) | 557.20 | 13.60 | 0.657 | 0.339 |
| GCM HadCM3L-A area (35 Ma) | 557.30 | 13.70 | 0.652 | 0.353 |
| GCM HadCM3L-A area (15 Ma) | 559.40 | 15.80 | 0.605 | 0.316 |
| GCM PlaSim area (65 Ma) | 559.70 | 16.10 | 0.619 | 0.288 |
| GCM PlaSim area (35 Ma) | 560.30 | 16.70 | 0.606 | 0.293 |
| GCM PlaSim area (15 Ma) | 560.70 | 17.10 | 0.594 | 0.298 |
| GCM HadCM3L-B area (65 Ma) | 561.30 | 17.70 | 0.594 | 0.272 |
| Proxy area (65 Ma) | 562.50 | 18.90 | 0.550 | 0.151 |
| GCM HadCM3L-B area (35 Ma) | 562.60 | 19.00 | 0.559 | 0.255 |
| Proxy area (35 Ma) | 562.70 | 19.10 | 0.546 | 0.149 |
| Proxy area (15 Ma) | 563.30 | 19.70 | 0.532 | 0.143 |
| GCM HadCM3L-B area (15 Ma) | 563.40 | 19.80 | 0.538 | 0.245 |
| GCM HadCM3L-C area (15 Ma) | 566.10 | 22.50 | 0.447 | 0.133 |
| GCM HadCM3L-C area (35 Ma) | 566.40 | 22.80 | 0.436 | 0.108 |
| GCM HadCM3L-C area (65 Ma) | 566.50 | 22.90 | 0.433 | 0.102 |
| GCM CESM area (65 Ma) | 567.50 | 23.90 | 0.387 | 0.052 |
| GCM CESM area (15 Ma) | 567.60 | 24.00 | 0.369 | 0.053 |
| GCM CESM area (35 Ma) | 567.60 | 24.00 | 0.382 | 0.051 |
| **Birds — Total richness** | | | | |
| **Present-day area (broad)** | **860.10** | **0.00** | **0.509** | **0.110** |
| **GCM HadCM3L-A area (65 Ma)** | **860.70** | **0.60** | **0.526** | **0.100** |
| **GCM HadCM3L-A area (35 Ma)** | **860.80** | **0.70** | **0.521** | **0.101** |
| GCM PlaSim area (65 Ma) | 862.10 | 2.00 | 0.502 | 0.079 |
| GCM HadCM3L-B area (65 Ma) | 862.30 | 2.20 | 0.494 | 0.082 |
| GCM HadCM3L-A area (15 Ma) | 862.60 | 2.50 | 0.496 | 0.076 |
| GCM PlaSim area (35 Ma) | 862.90 | 2.80 | 0.489 | 0.071 |
| GCM HadCM3L-B area (35 Ma) | 863.00 | 2.90 | 0.480 | 0.075 |
| GCM HadCM3L-B area (15 Ma) | 864.00 | 3.90 | 0.465 | 0.062 |
| GCM PlaSim area (15 Ma) | 864.10 | 4.00 | 0.473 | 0.055 |
| GCM HadCM3L-C area (35 Ma) | 865.20 | 5.10 | 0.454 | 0.042 |
| GCM HadCM3L-C area (65 Ma) | 865.20 | 5.10 | 0.455 | 0.040 |
| GCM HadCM3L-C area (15 Ma) | 865.40 | 5.30 | 0.453 | 0.041 |
| GCM CESM area (65 Ma) | 866.20 | 6.10 | 0.449 | 0.023 |
| GCM CESM area (35 Ma) | 866.50 | 6.40 | 0.446 | 0.020 |
| GCM CESM area (15 Ma) | 866.90 | 6.80 | 0.442 | 0.014 |
| Proxy area (65 Ma) | 867.00 | 6.90 | 0.475 | 0.008 |
| Proxy area (35 Ma) | 867.10 | 7.00 | 0.475 | 0.008 |
| Proxy area (15 Ma) | 867.20 | 7.10 | 0.472 | 0.006 |
| **Birds — Resident richness** | | | | |
| **Present-day area (broad)** | **626.70** | **0.00** | **0.759** | **0.635** |
| GCM PlaSim area (15 Ma) | 640.80 | 14.10 | 0.349 | 0.244 |
| GCM HadCM3L-A area (35 Ma) | 641.10 | 14.40 | 0.349 | 0.231 |
| GCM PlaSim area (35 Ma) | 641.30 | 14.60 | 0.330 | 0.217 |
| GCM HadCM3L-A area (15 Ma) | 641.40 | 14.70 | 0.305 | 0.208 |
| GCM HadCM3L-A area (65 Ma) | 641.70 | 15.00 | 0.332 | 0.208 |
| GCM PlaSim area (65 Ma) | 642.10 | 15.40 | 0.296 | 0.185 |
| GCM HadCM3L-B area (15 Ma) | 643.10 | 16.40 | 0.241 | 0.164 |
| GCM HadCM3L-B area (65 Ma) | 643.30 | 16.60 | 0.261 | 0.149 |
| GCM HadCM3L-B area (35 Ma) | 643.40 | 16.70 | 0.245 | 0.148 |
| GCM HadCM3L-C area (15 Ma) | 643.60 | 16.90 | 0.210 | 0.139 |
| GCM HadCM3L-C area (35 Ma) | 644.00 | 17.30 | 0.197 | 0.116 |
| GCM HadCM3L-C area (65 Ma) | 644.30 | 17.60 | 0.181 | 0.103 |
| GCM CESM area (15 Ma) | 644.40 | 17.70 | 0.120 | 0.087 |
| GCM CESM area (35 Ma) | 644.80 | 18.10 | 0.125 | 0.072 |
| GCM CESM area (65 Ma) | 644.90 | 18.20 | 0.124 | 0.067 |
| Proxy area (15 Ma) | 645.50 | 18.80 | 0.166 | 0.053 |
| Proxy area (35 Ma) | 645.50 | 18.80 | 0.163 | 0.053 |
| Proxy area (65 Ma) | 645.50 | 18.80 | 0.158 | 0.052 |
| **Birds — Endemic richness** | | | | |
| **Present-day area (broad)** | **348.00** | **0.00** | **0.727** | **0.308** |
| GCM PlaSim area (65 Ma) | 353.10 | 5.10 | 0.593 | 0.079 |
| GCM PlaSim area (35 Ma) | 353.40 | 5.40 | 0.588 | 0.078 |
| GCM PlaSim area (15 Ma) | 353.60 | 5.60 | 0.582 | 0.077 |
| GCM HadCM3L-A area (65 Ma) | 353.80 | 5.80 | 0.541 | 0.057 |
| GCM CESM area (65 Ma) | 353.90 | 5.90 | 0.541 | 0.049 |
| GCM HadCM3L-A area (35 Ma) | 354.00 | 6.00 | 0.537 | 0.057 |
| GCM CESM area (35 Ma) | 354.00 | 6.00 | 0.532 | 0.048 |
| GCM HadCM3L-A area (15 Ma) | 354.10 | 6.10 | 0.534 | 0.052 |
| GCM CESM area (15 Ma) | 354.10 | 6.10 | 0.514 | 0.044 |
| Proxy area (15 Ma) | 354.20 | 6.20 | 0.527 | 0.031 |
| Proxy area (35 Ma) | 354.20 | 6.20 | 0.530 | 0.032 |
| Proxy area (65 Ma) | 354.20 | 6.20 | 0.531 | 0.033 |
| GCM HadCM3L-B area (65 Ma) | 354.50 | 6.50 | 0.530 | 0.033 |
| GCM HadCM3L-B area (15 Ma) | 354.90 | 6.90 | 0.517 | 0.024 |
| GCM HadCM3L-B area (35 Ma) | 354.90 | 6.90 | 0.517 | 0.023 |
| GCM HadCM3L-C area (65 Ma) | 355.00 | 7.00 | 0.517 | 0.016 |
| GCM HadCM3L-C area (15 Ma) | 355.10 | 7.10 | 0.508 | 0.013 |
| GCM HadCM3L-C area (35 Ma) | 355.10 | 7.10 | 0.514 | 0.015 |
| **Mammals — Total richness** | | | | |
| **Present-day area (broad)** | **775.70** | **0.00** | **0.460** | **0.233** |
| GCM HadCM3L-A area (65 Ma) | 779.70 | 4.00 | 0.438 | 0.164 |
| GCM HadCM3L-A area (35 Ma) | 780.00 | 4.30 | 0.431 | 0.165 |
| GCM PlaSim area (65 Ma) | 781.60 | 5.90 | 0.398 | 0.130 |
| GCM HadCM3L-B area (65 Ma) | 781.90 | 6.20 | 0.385 | 0.137 |
| GCM HadCM3L-A area (15 Ma) | 782.30 | 6.60 | 0.389 | 0.125 |
| GCM PlaSim area (35 Ma) | 782.50 | 6.80 | 0.382 | 0.119 |
| GCM HadCM3L-B area (35 Ma) | 783.10 | 7.40 | 0.361 | 0.121 |
| GCM PlaSim area (15 Ma) | 783.80 | 8.10 | 0.360 | 0.102 |
| GCM HadCM3L-B area (15 Ma) | 784.30 | 8.60 | 0.339 | 0.101 |
| GCM HadCM3L-C area (35 Ma) | 785.90 | 10.20 | 0.316 | 0.068 |
| GCM HadCM3L-C area (15 Ma) | 786.00 | 10.30 | 0.315 | 0.067 |
| GCM HadCM3L-C area (65 Ma) | 786.00 | 10.30 | 0.316 | 0.063 |
| Proxy area (65 Ma) | 787.00 | 11.30 | 0.377 | 0.028 |
| Proxy area (35 Ma) | 787.10 | 11.40 | 0.376 | 0.026 |
| Proxy area (15 Ma) | 787.40 | 11.70 | 0.372 | 0.023 |
| GCM CESM area (65 Ma) | 787.40 | 11.70 | 0.310 | 0.029 |
| GCM CESM area (35 Ma) | 787.80 | 12.10 | 0.308 | 0.024 |
| GCM CESM area (15 Ma) | 788.20 | 12.50 | 0.307 | 0.016 |
| **Mammals — Resident richness** | | | | |
| **Present-day area (broad)** | **600.70** | **0.00** | **0.628** | **0.611** |
| GCM HadCM3L-A area (35 Ma) | 613.20 | 12.50 | 0.260 | 0.260 |
| GCM HadCM3L-A area (65 Ma) | 613.60 | 12.90 | 0.242 | 0.242 |
| GCM HadCM3L-A area (15 Ma) | 613.80 | 13.10 | 0.233 | 0.233 |
| GCM PlaSim area (15 Ma) | 613.90 | 13.20 | 0.228 | 0.228 |
| GCM PlaSim area (35 Ma) | 614.00 | 13.30 | 0.206 | 0.206 |
| GCM PlaSim area (65 Ma) | 614.30 | 13.60 | 0.194 | 0.194 |
| GCM HadCM3L-B area (65 Ma) | 615.30 | 14.60 | 0.174 | 0.174 |
| GCM HadCM3L-B area (15 Ma) | 615.80 | 15.10 | 0.169 | 0.169 |
| GCM HadCM3L-B area (35 Ma) | 615.80 | 15.10 | 0.164 | 0.164 |
| GCM HadCM3L-C area (15 Ma) | 616.80 | 16.10 | 0.121 | 0.121 |
| GCM HadCM3L-C area (35 Ma) | 617.00 | 16.30 | 0.109 | 0.109 |
| GCM HadCM3L-C area (65 Ma) | 617.30 | 16.60 | 0.098 | 0.098 |
| GCM CESM area (15 Ma) | 617.80 | 17.10 | 0.066 | 0.066 |
| GCM CESM area (35 Ma) | 617.90 | 17.20 | 0.065 | 0.065 |
| GCM CESM area (65 Ma) | 617.90 | 17.20 | 0.065 | 0.065 |
| Proxy area (65 Ma) | 618.00 | 17.30 | 0.105 | 0.081 |
| Proxy area (15 Ma) | 618.10 | 17.40 | 0.104 | 0.077 |
| Proxy area (35 Ma) | 618.10 | 17.40 | 0.108 | 0.080 |
| **Mammals — Endemic richness** | | | | |
| **Present-day area (broad)** | **415.00** | **0.00** | **0.722** | **0.493** |
| GCM HadCM3L-A area (65 Ma) | 424.60 | 9.60 | 0.435 | 0.182 |
| GCM HadCM3L-A area (35 Ma) | 424.80 | 9.80 | 0.427 | 0.185 |
| GCM HadCM3L-A area (15 Ma) | 425.50 | 10.50 | 0.386 | 0.162 |
| Proxy area (65 Ma) | 425.70 | 10.70 | 0.392 | 0.114 |
| GCM PlaSim area (65 Ma) | 425.70 | 10.70 | 0.399 | 0.128 |
| Proxy area (35 Ma) | 425.80 | 10.80 | 0.389 | 0.112 |
| GCM PlaSim area (35 Ma) | 425.90 | 10.90 | 0.386 | 0.126 |
| Proxy area (15 Ma) | 426.00 | 11.00 | 0.376 | 0.108 |
| GCM PlaSim area (15 Ma) | 426.20 | 11.20 | 0.376 | 0.129 |
| GCM HadCM3L-B area (65 Ma) | 426.70 | 11.70 | 0.353 | 0.105 |
| GCM HadCM3L-B area (35 Ma) | 427.50 | 12.50 | 0.311 | 0.076 |
| GCM HadCM3L-B area (15 Ma) | 427.70 | 12.70 | 0.297 | 0.069 |
| GCM CESM area (35 Ma) | 428.10 | 13.10 | 0.236 | 0.037 |
| GCM CESM area (65 Ma) | 428.10 | 13.10 | 0.240 | 0.036 |
| GCM CESM area (15 Ma) | 428.20 | 13.20 | 0.221 | 0.036 |
| GCM HadCM3L-C area (15 Ma) | 428.40 | 13.40 | 0.259 | 0.029 |
| GCM HadCM3L-C area (35 Ma) | 428.50 | 13.50 | 0.258 | 0.026 |
| GCM HadCM3L-C area (65 Ma) | 428.50 | 13.50 | 0.257 | 0.021 |

**Table G.** Model selection and explained variance (pseudo-R²) estimates from 11 models of species diversity estimated with three measures (total, resident, and endemic), comparing present-day biome area with minimum biome area and time-integrated biome area derived from five different climate models (HadCM3L-A, HadCM3L-B, HadCM3L-C, CESM, PlaSim). Cond. R² is the conditional pseudo-R² value including fixed and random effects. Marg. R² is the marginal pseudo-R² of the fixed effects. AIC = Akaike Information Criteria. Best fitting models (ΔAIC < 2) are highlighted in bold.

| Model | AIC | ΔAIC | Cond. R² | Marg. R² |
| --- | --- | --- | --- | --- |
| **Amphibians — Total richness** | | | | |
| **Present-day area** | **717.80** | **0.00** | **0.632** | **0.406** |
| minArea HadCM3L-B | 719.80 | 2.00 | 0.616 | 0.346 |
| minArea HadCM3L-A | 724.10 | 6.30 | 0.540 | 0.281 |
| timeArea HadCM3L-A (35 Ma) | 728.20 | 10.40 | 0.559 | 0.241 |
| timeArea HadCM3L-B (35 Ma) | 730.80 | 13.00 | 0.520 | 0.201 |
| timeArea HadCM3L-C (35 Ma) | 732.50 | 14.70 | 0.510 | 0.170 |
| minArea CESM | 733.20 | 15.40 | 0.424 | 0.162 |
| minArea PlaSim | 734.00 | 16.20 | 0.419 | 0.098 |
| minArea HadCM3L-C | 735.20 | 17.40 | 0.396 | 0.126 |
| timeArea CESM (35 Ma) | 737.20 | 19.40 | 0.382 | 0.057 |
| timeArea PlaSim (35 Ma) | 737.80 | 20.00 | 0.374 | 0.030 |
| **Amphibians — Resident richness** | | | | |
| **Present-day area** | **596.00** | **0.00** | **0.730** | **0.622** |
| minArea HadCM3L-B | 603.10 | 7.10 | 0.666 | 0.457 |
| minArea HadCM3L-A | 607.40 | 11.40 | 0.558 | 0.399 |
| timeArea HadCM3L-A (35 Ma) | 610.00 | 14.00 | 0.658 | 0.418 |
| timeArea HadCM3L-B (35 Ma) | 614.40 | 18.40 | 0.569 | 0.297 |
| timeArea HadCM3L-C (35 Ma) | 616.80 | 20.80 | 0.528 | 0.225 |
| minArea PlaSim | 617.30 | 21.30 | 0.377 | 0.150 |
| minArea CESM | 619.20 | 23.20 | 0.416 | 0.158 |
| timeArea PlaSim (35 Ma) | 619.70 | 23.70 | 0.333 | 0.106 |
| timeArea CESM (35 Ma) | 619.80 | 23.80 | 0.351 | 0.127 |
| minArea HadCM3L-C | 620.80 | 24.80 | 0.344 | 0.104 |
| **Amphibians — Endemic richness** | | | | |
| **minArea HadCM3L-B** | **504.40** | **0.00** | **0.676** | **0.424** |
| **Present-day area** | **504.50** | **0.10** | **0.676** | **0.504** |
| minArea HadCM3L-A | 508.50 | 4.10 | 0.569 | 0.378 |
| timeArea HadCM3L-A (35 Ma) | 511.90 | 7.50 | 0.652 | 0.367 |
| timeArea HadCM3L-B (35 Ma) | 515.20 | 10.80 | 0.582 | 0.256 |
| minArea PlaSim | 516.80 | 12.40 | 0.422 | 0.138 |
| timeArea HadCM3L-C (35 Ma) | 517.60 | 13.20 | 0.542 | 0.181 |
| timeArea CESM (35 Ma) | 518.20 | 13.80 | 0.426 | 0.169 |
| minArea CESM | 519.90 | 15.50 | 0.448 | 0.102 |
| timeArea PlaSim (35 Ma) | 520.10 | 15.70 | 0.361 | 0.070 |
| minArea HadCM3L-C | 520.30 | 15.90 | 0.428 | 0.089 |
| **Reptiles — Total richness** | | | | |
| **Present-day area** | **757.00** | **0.00** | **0.668** | **0.453** |
| minArea HadCM3L-A | 773.70 | 16.70 | 0.442 | 0.264 |
| timeArea HadCM3L-A (35 Ma) | 774.80 | 17.80 | 0.468 | 0.249 |
| minArea HadCM3L-B | 776.00 | 19.00 | 0.438 | 0.216 |
| timeArea HadCM3L-B (35 Ma) | 777.40 | 20.40 | 0.415 | 0.215 |
| timeArea HadCM3L-C (35 Ma) | 779.70 | 22.70 | 0.389 | 0.182 |
| minArea PlaSim | 779.80 | 22.80 | 0.357 | 0.123 |
| minArea CESM | 781.10 | 24.10 | 0.334 | 0.145 |
| minArea HadCM3L-C | 784.20 | 27.20 | 0.276 | 0.080 |
| timeArea PlaSim (35 Ma) | 785.80 | 28.80 | 0.284 | 0.011 |
| timeArea CESM (35 Ma) | 786.40 | 29.40 | 0.303 | 0.003 |
| **Reptiles — Resident richness** | | | | |
| **Present-day area** | **618.50** | **0.00** | **0.830** | **0.688** |
| timeArea HadCM3L-A (35 Ma) | 655.70 | 37.20 | 0.483 | 0.321 |
| minArea HadCM3L-A | 657.50 | 39.00 | 0.363 | 0.258 |
| timeArea HadCM3L-B (35 Ma) | 658.30 | 39.80 | 0.395 | 0.256 |
| minArea PlaSim | 660.30 | 41.80 | 0.286 | 0.162 |
| minArea HadCM3L-B | 660.40 | 41.90 | 0.336 | 0.174 |
| timeArea HadCM3L-C (35 Ma) | 660.60 | 42.10 | 0.329 | 0.194 |
| minArea CESM | 663.90 | 45.40 | 0.231 | 0.087 |
| timeArea PlaSim (35 Ma) | 663.90 | 45.40 | 0.157 | 0.068 |
| minArea HadCM3L-C | 665.50 | 47.00 | 0.164 | 0.040 |
| timeArea CESM (35 Ma) | 666.40 | 47.90 | 0.190 | 0.001 |
| **Reptiles — Endemic richness** | | | | |
| **Present-day area** | **530.00** | **0.00** | **0.817** | **0.581** |
| timeArea HadCM3L-A (35 Ma) | 561.20 | 31.20 | 0.511 | 0.224 |
| minArea HadCM3L-A | 561.40 | 31.40 | 0.442 | 0.214 |
| minArea PlaSim | 562.80 | 32.80 | 0.410 | 0.138 |
| timeArea HadCM3L-B (35 Ma) | 563.90 | 33.90 | 0.440 | 0.149 |
| minArea HadCM3L-B | 564.60 | 34.60 | 0.424 | 0.110 |
| timeArea HadCM3L-C (35 Ma) | 565.40 | 35.40 | 0.409 | 0.102 |
| timeArea PlaSim (35 Ma) | 567.70 | 37.70 | 0.354 | 0.019 |
| minArea CESM | 567.80 | 37.80 | 0.373 | 0.028 |
| minArea HadCM3L-C | 568.40 | 38.40 | 0.371 | 0.008 |
| timeArea CESM (35 Ma) | 568.50 | 38.50 | 0.416 | 0.003 |
| **Birds — Total richness** | | | | |
| **Present-day area** | **843.40** | **0.00** | **0.643** | **0.292** |
| minArea HadCM3L-B | 847.50 | 4.10 | 0.615 | 0.240 |
| minArea HadCM3L-A | 852.40 | 9.00 | 0.556 | 0.187 |
| timeArea HadCM3L-C (35 Ma) | 852.90 | 9.50 | 0.594 | 0.223 |
| timeArea HadCM3L-A (35 Ma) | 854.80 | 11.40 | 0.561 | 0.167 |
| timeArea HadCM3L-B (35 Ma) | 856.10 | 12.70 | 0.544 | 0.166 |
| minArea PlaSim | 859.30 | 15.90 | 0.496 | 0.101 |
| minArea HadCM3L-C | 859.40 | 16.00 | 0.467 | 0.149 |
| minArea CESM | 859.90 | 16.50 | 0.459 | 0.125 |
| timeArea PlaSim (35 Ma) | 865.60 | 22.20 | 0.449 | 0.023 |
| timeArea CESM (35 Ma) | 866.10 | 22.70 | 0.442 | 0.023 |
| **Birds — Resident richness** | | | | |
| **Present-day area** | **576.70** | **0.00** | **0.907** | **0.794** |
| timeArea HadCM3L-A (35 Ma) | 632.10 | 55.40 | 0.602 | 0.426 |
| timeArea HadCM3L-B (35 Ma) | 635.40 | 58.70 | 0.553 | 0.366 |
| minArea HadCM3L-B | 636.20 | 59.50 | 0.498 | 0.345 |
| minArea HadCM3L-A | 636.30 | 59.60 | 0.401 | 0.336 |
| timeArea HadCM3L-C (35 Ma) | 638.00 | 61.30 | 0.509 | 0.304 |
| minArea PlaSim | 638.70 | 62.00 | 0.272 | 0.272 |
| timeArea PlaSim (35 Ma) | 641.50 | 64.80 | 0.203 | 0.188 |
| minArea CESM | 644.40 | 67.70 | 0.210 | 0.097 |
| timeArea CESM (35 Ma) | 645.00 | 68.30 | 0.136 | 0.066 |
| minArea HadCM3L-C | 645.30 | 68.60 | 0.122 | 0.063 |
| **Birds — Endemic richness** | | | | |
| **Present-day area** | **335.70** | **0.00** | **0.807** | **0.493** |
| minArea PlaSim | 342.00 | 6.30 | 0.603 | 0.418 |
| minArea HadCM3L-B | 345.90 | 10.20 | 0.626 | 0.304 |
| timeArea PlaSim (35 Ma) | 346.80 | 11.10 | 0.849 | 0.652 |
| minArea HadCM3L-A | 347.70 | 12.00 | 0.548 | 0.281 |
| timeArea HadCM3L-A (35 Ma) | 349.90 | 14.20 | 0.617 | 0.212 |
| timeArea HadCM3L-B (35 Ma) | 352.20 | 16.50 | 0.562 | 0.118 |
| timeArea HadCM3L-C (35 Ma) | 353.10 | 17.40 | 0.545 | 0.075 |
| minArea HadCM3L-C | 354.60 | 18.90 | 0.531 | 0.029 |
| timeArea CESM (35 Ma) | 354.70 | 19.00 | 0.458 | 0.027 |
| minArea CESM | 354.80 | 19.10 | 0.516 | 0.018 |
| **Mammals — Total richness** | | | | |
| **Present-day area** | **753.90** | **0.00** | **0.659** | **0.460** |
| minArea HadCM3L-B | 765.40 | 11.50 | 0.559 | 0.339 |
| minArea HadCM3L-A | 768.40 | 14.50 | 0.510 | 0.300 |
| timeArea HadCM3L-A (35 Ma) | 771.50 | 17.60 | 0.511 | 0.273 |
| timeArea HadCM3L-C (35 Ma) | 774.30 | 20.40 | 0.483 | 0.270 |
| timeArea HadCM3L-B (35 Ma) | 774.40 | 20.50 | 0.471 | 0.253 |
| minArea CESM | 778.80 | 24.90 | 0.363 | 0.215 |
| minArea PlaSim | 778.80 | 24.90 | 0.408 | 0.147 |
| minArea HadCM3L-C | 781.10 | 27.20 | 0.340 | 0.192 |
| timeArea PlaSim (35 Ma) | 785.90 | 32.00 | 0.332 | 0.041 |
| timeArea CESM (35 Ma) | 787.30 | 33.40 | 0.316 | 0.029 |
| **Mammals — Resident richness** | | | | |
| **Present-day area** | **565.80** | **0.00** | **0.840** | **0.788** |
| timeArea HadCM3L-A (35 Ma) | 604.60 | 38.80 | 0.507 | 0.471 |
| minArea HadCM3L-A | 606.20 | 40.40 | 0.389 | 0.389 |
| minArea HadCM3L-B | 607.40 | 41.60 | 0.404 | 0.376 |
| timeArea HadCM3L-B (35 Ma) | 608.80 | 43.00 | 0.366 | 0.346 |
| timeArea HadCM3L-C (35 Ma) | 611.50 | 45.70 | 0.284 | 0.263 |
| minArea PlaSim | 612.40 | 46.60 | 0.222 | 0.222 |
| timeArea PlaSim (35 Ma) | 615.10 | 49.30 | 0.144 | 0.144 |
| minArea CESM | 616.60 | 50.80 | 0.103 | 0.103 |
| timeArea CESM (35 Ma) | 617.80 | 52.00 | 0.074 | 0.074 |
| minArea HadCM3L-C | 617.90 | 52.10 | 0.075 | 0.075 |
| **Mammals — Endemic richness** | | | | |
| **Present-day area** | **399.90** | **0.00** | **0.780** | **0.583** |
| minArea HadCM3L-A | 417.50 | 17.60 | 0.419 | 0.419 |
| minArea HadCM3L-B | 419.00 | 19.10 | 0.430 | 0.337 |
| timeArea HadCM3L-A (35 Ma) | 421.50 | 21.60 | 0.462 | 0.284 |
| minArea PlaSim | 422.30 | 22.40 | 0.298 | 0.207 |
| timeArea HadCM3L-B (35 Ma) | 424.30 | 24.40 | 0.356 | 0.170 |
| timeArea HadCM3L-C (35 Ma) | 426.00 | 26.10 | 0.304 | 0.107 |
| timeArea PlaSim (35 Ma) | 426.70 | 26.80 | 0.250 | 0.080 |
| minArea CESM | 428.50 | 28.60 | 0.245 | 0.017 |
| minArea HadCM3L-C | 428.60 | 28.70 | 0.238 | 0.015 |
| timeArea CESM (35 Ma) | 428.70 | 28.80 | 0.206 | 0.013 |

**Table H.** Species overlap among realm-biome units, at the secondary Koppen classification used in the main analyses and at the primary Koppen classification of the validation. Overlap is the median Jaccard similarity of species composition: for each realm-biome unit the median Jaccard similarity against all other units, then the median of those values across units, expressed as a percentage. Species = number of species contributing to each richness metric. Resident and endemic richness assign each species to a single unit, so their overlap is identically zero by construction.

| **Class** | **Classification level** | **Richness metric** | **Species** | **Median Jaccard (%)** |
| --- | --- | --- | --- | --- |
| Amphibians | Secondary Koppen | Total | 6,898 | 0.07 |
|  |  | Resident | 6,898 | NA |
|  |  | Endemic | 3,170 | NA |
|  | Primary Koppen | Total | 6,898 | 0.15 |
|  |  | Resident | 6,898 | NA |
|  |  | Endemic | 4,174 | NA |
| Reptiles | Secondary Koppen | Total | 8,389 | 0.11 |
|  |  | Resident | 8,389 | NA |
|  |  | Endemic | 3,736 | NA |
|  | Primary Koppen | Total | 8,389 | 0.09 |
|  |  | Resident | 8,389 | NA |
|  |  | Endemic | 4,890 | NA |
| Birds | Secondary Koppen | Total | 9,330 | 2.10 |
|  |  | Resident | 9,330 | NA |
|  |  | Endemic | 881 | NA |
|  | Primary Koppen | Total | 9,330 | 2.22 |
|  |  | Resident | 9,330 | NA |
|  |  | Endemic | 1,951 | NA |
| Mammals | Secondary Koppen | Total | 5,515 | 3.48 |
|  |  | Resident | 5,515 | NA |
|  |  | Endemic | 1,040 | NA |
|  | Primary Koppen | Total | 5,515 | 2.82 |
|  |  | Resident | 5,515 | NA |
|  |  | Endemic | 1,775 | NA |

## Supplementary Figures

**Fig A.** Distribution of Köppen biomes at 5 Ma intervals for the Cenozoic period from the HadCM3L-A climate model.

**Fig B.** Distribution of Köppen biomes at 5 Ma intervals for the Cenozoic period from the HadCM3L-B climate model.

**Fig C.** Distribution of Köppen biomes at 5 Ma intervals for the Cenozoic period from the HadCM3L-C climate model.

**Fig D.** Distribution of Köppen biomes at 5 Ma intervals for the Cenozoic period from the CESM climate model.

**Fig E.** Distribution of Köppen biomes at 5 Ma intervals for the Cenozoic period from the PlaSim climate model.

**Fig F.** Distribution of Köppen biomes at 5 Ma intervals for the Cenozoic period, hand drawn based on the lithological and fossil record. A = tropical, B = arid, C = subtropical/temperate, D = cold/continental, E = polar.

**Fig G.** Agreement between climate-models and the lithological and fossil proxy record. (A) Cohen’s κ of cell-to-cell comparison of the primary Koppen-biome levels between the hand drawn, proxy-based reconstructions of Scotese and each of the five climate models used in this study at each time interval. Following the convention establish in Landis & Koch (1977), κ < 0.2 = poor agreement; 0.4 > κ > 0.2 = fair agreement; 0.6 > κ > 0.4 = moderate agreement; 0.8 > κ > 0.6 = substantial agreement. (B) The percentage of individual proxy records in agreement with each climate model (coloured lines) and the hand-drawn reconstruction (dashed line) at major time intervals.

**Fig H.** The proportion of each biome across six biogeographic realms through time for the HadCM3L-A climate model. Coloured by secondary Köppen classifications.

**Fig I.** The proportion of each biome across six biogeographic realms through time for the HadCM3L-B climate model. Coloured by secondary Köppen classifications.

**Fig J.** The proportion of each biome across six biogeographic realms through time for the HadCM3L-C climate model.

**Fig K.** The proportion of each biome across six biogeographic realms through time for the CESM climate model.

**Fig L.** The proportion of each biome across six biogeographic realms through time for the PlaSim climate model.

**Fig M.** Area of each biome across six biogeographic realms through time for the HadCM3L-A climate model. Coloured by biogeographic realm. Af = tropical, rainforest, Am = tropical, monsoonal, Aw = tropical, savannah, BW = arid, desert, BS = semi-arid, steppe, Cf = temperate, Cw = temperate, dry winter, Cs = temperate, dry summer, Df = cold, Ds = cold, dry summer, Dw = cold, dry winter, ET = tundra.

**Fig N.** Area of each biome across six biogeographic realms through time for the HadCM3L-B climate model. Coloured by biogeographic realm. Af = tropical, rainforest, Am = tropical, monsoonal, Aw = tropical, savannah, BW = arid, desert, BS = semi-arid, steppe, Cf = temperate, Cw = temperate, dry winter, Cs = temperate, dry summer, Df = cold, Ds = cold, dry summer, Dw = cold, dry winter, ET = tundra.

**Fig O.** Area of each biome across six biogeographic realms through time for the HadCM3L-C climate model. Coloured by biogeographic realm. Af = tropical, rainforest, Am = tropical, monsoonal, Aw = tropical, savannah, BW = arid, desert, BS = semi-arid, steppe, Cf = temperate, Cw = temperate, dry winter, Cs = temperate, dry summer, Df = cold, Ds = cold, dry summer, Dw = cold, dry winter, ET = tundra.

**Fig P.** Area of each biome across six biogeographic realms through time for the CESM climate model. Coloured by biogeographic realm. Af = tropical, rainforest, Am = tropical, monsoonal, Aw = tropical, savannah, BW = arid, desert, BS = semi-arid, steppe, Cf = temperate, Cw = temperate, dry winter, Cs = temperate, dry summer, Df = cold, Ds = cold, dry summer, Dw = cold, dry winter, ET = tundra.

**Fig Q.** Area of each biome across six biogeographic realms through time for the PlaSim climate model. Coloured by biogeographic realm. Af = tropical, rainforest, Am = tropical, monsoonal, Aw = tropical, savannah, BW = arid, desert, BS = semi-arid, steppe, Cf = temperate, Cw = temperate, dry winter, Cs = temperate, dry summer, Df = cold, Ds = cold, dry summer, Dw = cold, dry winter, ET = tundra.

## References

Brenning, A. (2008). Statistical geocomputing combining R and SAGA: The example of landslide susceptibility analysis with generalized additive models. *Hambg. Beitr. Zur Phys. Geogr. Landschaftsökologie*, 19, 410.

Espy, J. (1836). Essays on meteorology. No. IV. *J. Frankl. Inst.*, 22, 239–246.

Foster, G.L., Royer, D.L. & Lunt, D.J. (2017). Future climate forcing potentially without precedent in the last 420 million years. *Nat. Commun.*, 8, 14845.

Irvine, P.J., Gregoire, L.J., Lunt, D.J. & Valdes, P.J. (2013). An efficient method to generate a perturbed parameter ensemble of a fully coupled AOGCM without flux-adjustment. *Geosci. Model Dev.*, 6, 1447–1462.

Kalmus, P., Ao, C.O., Wang, K.-N., Manzi, M.P. & Teixeira, J. (2022). A high-resolution planetary boundary layer height seasonal climatology from GNSS radio occultations. *Remote Sens. Environ.*, 276, 113037.

Karger, D.N., Conrad, O., Böhner, J., Kawohl, T., Kreft, H., Soria-Auza, R.W., *et al.* (2017). Climatologies at high resolution for the earth’s land surface areas. *Sci. Data*, 4, 1–20.

Kiehl, J.T. & Shields, C.A. (2013). Sensitivity of the Palaeocene–Eocene Thermal Maximum climate to cloud properties. *Philos. Trans. R. Soc. Math. Phys. Eng. Sci.*, 371, 20130093.

Landis, J.R. & Koch, G.G. (1977). The Measurement of Observer Agreement for Categorical Data. *Biometrics*, 33, 159.

Lawrence, M.G. (2005). The Relationship between Relative Humidity and the Dewpoint Temperature in Moist Air: A Simple Conversion and Applications. *Bull. Am. Meteorol. Soc.*, 86, 225–234.

Rae, J.W.B., Zhang, Y.G., Liu, X., Foster, G.L., Stoll, H.M. & Whiteford, R.D.M. (2021). Atmospheric CO_2_ over the Past 66 Million Years from Marine Archives. *Annu. Rev. Earth Planet. Sci.*, 49, 609–641.

Ren, X., Valdes, P., Lunt, D.J., Haywood, A.M., Hendy, E., Tindall, J.C., *et al.* (2025). Impact of Model Tuning on Simulated Mid‐Pliocene Warming. *Paleoceanogr. Paleoclimatology*, 40, e2024PA004976.

Sagoo, N., Valdes, P., Flecker, R. & Gregoire, L.J. (2013). The Early Eocene equable climate problem: can perturbations of climate model parameters identify possible solutions? *Philos. Trans. R. Soc. Math. Phys. Eng. Sci.*, 371, 20130123.

Valdes, P.J., Scotese, C.R. & Lunt, D.J. (2021). Deep ocean temperatures through time. *Clim. Past*, 17, 1483–1506.
