# Supplementary material for: High-resolution paleobiome reconstructions highlight ecological and historical dynamics underpinning global terrestrial vertebrate diversity
Source: PLoS Biol. 2026 Oct 23;24(10):e3004077. doi: 10.1371/journal.pbio.3004077 (PMC13719045; doi:10.1371/journal.pbio.3004077)
Supplement: Supplementary file 2 [file pbio.3004077.s005.docx]

REVIEWS:


Reviewer #1: In my previous review, I had two major concerns, and both have been very satisfactorily addressed. First, I pointed out the discrepancies between the models reconstructing the history of biomes and asked if these reconstructions are reliable and if they can be used for the intended inferences. The authors have now taken this very seriously, and provide a nice overview of the models, their similarities, differences and limitations. It makes the whole study much more robust and convincing, and it itself is very valuable - and quite novel and surprising.

Thank you for taking the time to review our manuscript.

Second, I was not entirely happy with the theoretical foundation of the findings in the previous version. Now the authors frame it using equilibrium biodiversity dynamics, and it is very clear. In this respect, I would still ask for a slightly better treatment of the literature - perhaps not by citing more references, but a bit better working with them. However, I am certainly biased in this respect, as I devoted last 15 years to the formalization and development of the equilibrium theory of biodiversity dynamics. I would just expect that some references could be put in the text at places where the authors describe the dynamics which is in precise accord with these formalizations, admitting that the findings agree with the predictions based on these formalizations. Also, I have spotted that (from my point of view) the key reference in this respect occurs in reference lists twice, as ref. 58 and ref. 72 - did the authors want to cite as the other reference the newer paper of the same set of authors (<https://aus01.safelinks.protection.outlook.com/?url=https%3A%2F%2Fdoi.org%2F10.1016%2Fj.tree.2026.05.011&data=05%7C02%7Calexander.skeels%40anu.edu.au%7C427b28e481d14359b21608def93b4ecc%7Ce37d725cab5c46249ae5f0533e486437%7C0%7C0%7C639222230337464181%7CUnknown%7CTWFpbGZsb3d8eyJFbXB0eU1hcGkiOnRydWUsIlYiOiIwLjAuMDAwMCIsIlAiOiJXaW4zMiIsIkFOIjoiTWFpbCIsIldUIjoyfQ%3D%3D%7C0%7C%7C%7C&sdata=eeKJJfRHVLND%2BlbfRIWnP9HuvqpJhEytsUbc6Vd307k%3D&reserved=0>), which is a response to a comment to ref. 58? I appreciate the theoretical treatment, I would just be more happy if the context of previous theoretical results would be better integrated.

We have added the suggested reference and more clearly signposted how our results support the ETBD model in the discussion section (L334-335).

Perhaps more importantly, I feel it would be good to better utilize (in the Discussion section) the recent paper Cerezer et al. 2026 published also in PLoS Biology (now cited as ref. 67; note that the reference in the list contains by mistake the name of the editor). This paper quite closely mirrors the findings presented by the authors, including specific results for different taxa (e.g. different factors affecting Squamates in comparison to other groups). Indeed, both these studies appear complementary - both are dealing with historical vs. equilibrium explanations of global vertebrate diversity patterns, although Cerezer et al. use phylogenetic information to infer history, while Skeels et al. use historical reconstruction of biomes - but both converge to exactly the same conclusions, which seems nice.

We have now highlighted the comparison with that study in more detail. It was not published when we wrote the first draft of this manuscript (L343-347).

I have also a few concrete comments to particular places in the text as follows; all of them are relatively minor and aim just to an improvement of the text which is already quite good and potentially important.

Lines 56-60: Here some reference concerning the equilibrium theory would seem very appropriate, as this idea has been recently thoroughly developed and formalized, as mentioned above.

We have added additional references throughout this section to the ETBD and other equilibrium and non-equilibrium hypotheses.

Line 67: Missing "of": "of biodiversity".

Fixed.

Line 84: "tropical biomes are spatially constricted by arid conditions" - yes, but some arid biomes are also tropical, right? At least some savannas are arid. I know Koppen classification distinguish tropical and arid biomes, but this sentence could be misleading.

Following the Koppen definition, tropical here refers to the climate not the latitude, but we have now changed to “mesic tropical biomes” to avoid confusion.

Lines 100-101: Here it came to my mind that some other references could be also appropriate, as this process is described in ref. 58, and the concept of carrying capacity is not trivial, as described in Storch & Okie 2019, GEB 28: 1519. Carrying capacity is also mentioned on line 314. Again, not a crucial issue.

We have now cited the suggested reference.

Line 145: This subtitle does not make sense, as there is no other subtitle in the chapter. I would recommend splitting the chapter to several subchapters with respective subtitles.

Agreed, we have now added subtitles to each section.

Line 231: It is unclear, do you mean "had only marginally better fit" (instead of "was…poorer fit") or "provided only marginally better fit"?

We meant that the two models had a comparable fit, as the delta AIC was < 2. We have changed the text to reflect this (L242).

Fig. 3: Do I read it correctly that the best model (except for Squamates) included only productivity and temperature, and not area? Isn't it worth discussing? Also, the figure should be redrawn; now too much space is devoted to panel A (which can be squeezed in the left-right direction), while the crucial results in panels B and C are too small, and especially the font of the labels is extremely tiny.

For squamates, the best fit is area + temperature, not productivity. We have redesigned the Figure 3 following the reviewer’s suggestion.

Lines 267-268: Although I do appreciate the new treatment of biomes and their historical reconstructions, it came to my mind whether the historical (e.g. Eocene) "temperate" forests are really analogical to contemporary temperate forests. As far as I know, these forests, according to fossil record, contained fauna which is now considered quite tropical. It could be discussed as a potential caveat, but it may not be necessary.

We expect the degree to which these biomes reflect contemporary analogues would differ on a taxon-to-taxon basis. Early Eocene temperate forests are most commonly warm temperate/subtropical rainforests and may be structurally and compositionally similar to tropical rainforests. For example, in Patagonia and Tasmania in the Eocene, rainforests are variably described as subtropical and tropical in the literature. This however, largely relates to inconsistent definitions of “tropical” (very well-articulated in this paper; Feeley and Stroud 2018; https://doi.org/10.21425/F5FBG38649). By using a singular, quantitative and objective biome definition in this study, we hope to skirt some of the issues introduced by multiple “tropical definitions”. Further, using the “endemic richness” response, we restrict the analysis to only species that are endemic to singular biomes.

Line 272: Missing "of": "of rainforest biome area".

Fixed

Lines 318-325: This description of equilibrium diversity dynamics is not a general description of any diversity-dependent dynamics, but it is a bit more specific, describing a particular depiction formalized by the Equilibrium Theory of Biodiversity Dynamics, ETBD (developed specifically in references 5, 56, 58). It is OK, and I of course appreciate it, being the author of it, but for a reader who would not be fully familiar with it it could be better to clarify it, since other concepts do not necessarily assume that diversity-dependence acts via population size-dependence of extinction and/or speciation, which is the assumption of ETBD.

We have clarified that we are referring to the ETBD specifically here (L347).

Lines 335-337: The observation that diversification (actually speciation, as extinction cannot be reliably measured) rates are higher in high latitudes can be also due to higher turnover in equilibrium/ carrying capacity (see ref 58 for details), i.e. not only due to out-of-equilibrium state of high latitudes. Both these views are reasonable.

This is an interesting point and warrants further testing. Yet at equilibrium, high and low rates of turnover could each give the same net diversification rate (approximately 0), so we don’t believe that contradicts our statement.

Lines 347-353: This is indeed exactly equivalent to the argumentation in ref. 58.

We have cited this now in this sentence specifically.

Lines 370-374: Cerezer et al. 2026, mentioned above, provide alternative explanation for a poor effect of productivity on Squamate diversity, namely that NPP is not a good surrogate for resource availability for purely secondary consumers like Squamates. I am not saying it is necessary the best explanation of the different correlates of Squamate diversity (in comparison to mammals, amphibians and birds), but it may be worth considering.

This is a good point, and we are aware of a compelling hypothesis for reptile diversity in arid environments which links richness to termite abundance, which can be decoupled from productivity (Morton and James, 1988). We now reference this idea on L395-397.

Lines 545-549: The description of the models and model selection should be expanded. It is not clear whether all possible combinations of variables were tested (156 models do not seem to include all the possible combinations), and how (based on which criteria) the 13 models were selected. I am aware that most of this information is in the supplementary material, but the basic procedures should be also in the main text.

We have now updated the methods to make the model selection step clearer (L577-584). We intentionally did not fit an exhaustive set of all predictor combinations because some variables are nested and colinear. Instead, we selected 13 alternative models (Fig. 3B) and fitted these for 4 vertebrate classes and 3 richness metrics (13 x 4 x 3 = 156).

David Storch

**-----------------------------------------------------------------------------------------------------------------------------------**

**Reviewer #2:** I appreciate the authors' efforts in producing their revised manuscript.  They redid most of their analyses and rewrote the introduction and discussion in line with the reviewers' constructive criticisms.  However, I still find myself unsatisfied with the manuscript and would like the authors to consider further analyses and interpretation.  Although I wouldn't want my objections to sink the paper, I do think at the very least that the authors should include the following points in their final submission as caveats.

We thank the reviewer for their feedback it has been genuinely helpful in clarifying our results. We are glad the reviewer appreciated the previous revision efforts and I believe our latest revision will ease remaining concerns.

First, I don't understand how we are supposed to evaluate the "validation" of their models that the authors now include in Figure S6.  How does one decide that the agreement between the models and expert proxy-based reconstruction is "substantial" or "moderate" as the authors assert in the text (lines 167-186)?  And since the expert proxy-based maps are only drawn for the realm (tropical -- not the Koppen biomes) how were these disagreements assessed? (see my criticism below on choice of Koppen biomes).   The most important areas of disagreement will be between known paleobotanical localities that show tropical moist forest taxa that the paleoclimate models are not showing as "tropical rain forest or monsoon forest" -- In my opinion, the whole reason that the authors are showing better agreement with present day models vs time-Area measures is because the paleoclimate models they are using are wildly overestimating the arid areas and underestimating tropical rain forest areas.  Looking at the tropical Americas and the Amazon, it is hard to reconcile everything I've learned from Carina Hoorn's and Kathy Willis and Alan Graham and Carlos Jaramillo's papers with these new paleoclimatic maps. In Figure 1, there is no rainforest in the Amazon 30 million years ago!  Inconceivable!  Similarly, experts in Mediterranean climates estimate that the biome is around 3 million years old (maximum 10 million years) -- and the paleomaps here show substantial Mediterranean ecosystems going back well into the Oligocene!

Some of these points we believe stem from a misunderstanding of the analyses and biome definition we use here, which differ from Jetz and Fine (2012), and also from some paleobotanical studies. We have revised the manuscript to make clearer these issues.

1. **Reclassification scores follow established convention.** “Substantial” and “moderate” are the conventional labels for Cohen's κ following Landis & Koch (1977): κ < 0.20 slight, 0.21–0.40 fair, 0.41–0.60 moderate, 0.61–0.80 substantial, 0.81–1.00 almost perfect. These thresholds were given only in the legend of Figure S7; we have now moved them into the main text at first use (L173-178) and cited Landis & Koch (new reference 41).

2. **Proxy-based maps are Koppen biomes not Realms.** We may not have been clear here, and we apologize for the confusion. The Köppen scheme is hierarchical, and its first tier distinguishes five climate classes: tropical (A), arid (B), temperate (C), cold/continental (D) and polar (E). This is not what we term a "realm"; realms in this study are the six biogeographic realms (Afrotropical, Australasian, Indomalayan, Nearctic, Neotropical, Palearctic) used to partition the biodiversity data. As such, we can directly compare our climate-model based first-tier Koppen maps, and the proxy-based first-tier Koppen maps. We have rephrased this in the results to make it clear that we are comparing like-with-like (L170-173):

“*We validated the climate-model derived reconstructions of paleobiomes by comparing the primary Köppen category (A=tropical, B=arid, C=temperate, D=cold/continental, E=polar) across the climate-models with primary Köppen categories from expert-drawn, proxy-based reconstructions [21]*”

3. **Paleoclimate models do not overestimate arid area compared to proxy-based estimates.** The reviewer suggests that present-day predictors outperform time-integrated predictors because our paleoclimate models underestimate tropical and overestimate arid area. If this were the case, then reconstructions that do not share this putative bias should restore the historical signal. We therefore repeated the analysis using time-integrated area derived from the expert-drawn, proxy-based reconstructions of Scotese et al. (2021) — that is, from the proxy-based maps themselves rather than from any climate model. Present-day area remained a better fit than proxy-derived time-integrated area for every vertebrate class and every richness metric. Climate-model-derived time-integrated area, in fact, fitted even better than the proxy-derived equivalent. In other words, this result does not depend on our climate models. We now make this point clearer when discussing these results (L315-320):

“*Models of species richness showed that variables representing present-day area and energy were better predictors than historical measures across vertebrate classes, climate models, species pools, and time intervals considered. This was also true when using climate-model derived estimates of paleobiomes and when using proxy-based estimates of paleobiomes from the lithological and fossil record, such that it is not a peculiarity of the climate models themselves.*”

Specifically, the reviewer stresses concern over the reconstruction of aridity in South America in the Eocene, as seen in Figure 1. To ease the reviewers concern, we would like to highlight that while aridity in northern South America is contentious and does disagree with the proxy record, this is only present in some of the climate-models (see figure below). The PlaSim model, for example, does not show this phenomenon. We have now replaced the maps in Figure 1 with the PlaSim model, as this model was best matched proxy data maps.

**Appendix Figure 1.** Proxy records from Boucot et al 2013 overlaid on the Early Eocene paleobiome reconstructions from five different climate models. Following the Boucot, coals are indicative of wet sites, evapoiates are indicative of arid sites, bauxites are indicative of tropical sites, and crocodilians are indicative of tropical or warm temperate (subtropical) sites.

Aridity in central South America, however, is less contentious. The presence of evaporites are proxies of an arid environment here, and in the figure above, we can see that the biome reconstructions agree with the proxy record. We also note that there is an absence of fossils in this part of South America (see figure below with Paleobiology Database infromation) and this may be because arid environments don’t preserve botanical fossils as well as mesic sites. Aridity is also present in Beerling & Woodward’s (2001) model used in Fine and Ree (2006), but not in the other reconstructions used in that study, and similarly a time-integrated area effect was also not recovered when using the Beerling and Woodward model, mirroring our own results. We believe the weight of evidence from both proxy and model-based reconstructions suggest the presence of aridity in central South America, and if true we believe this result may be more representative than those based on models of continuous tropical rainforest. Nonetheless, despite the debate around the presence of arid biomes in the Neotropics (which will not be resolved here), the Neotropical arid and tropical biomes reflect only a handful of points in our dataset and we currently devote a whole paragraph to the debate of this topic. We believe this sufficiently caveats the uncertainty around these data points but in no way do we believe that this specific contention is driving the overall pattern.

**Appendix Figure 2.** The Palaeobotanical record for South America in the Eocene from the PaleoBiology Database (https://paleobiodb.org; accessed 19/08/2026). There are almost no sites from western or central South America to validate paleobiome reconstructions. This figure includes data from Jaramillo and Wilf.

We do acknowledge that a further source of disagreement may be arising because our biome concept is different to that of Jetz & Fine (2012) and Fine and Ree (2006) and we have now made explicit signposting of these differences and what it means for comparing the studies (L139-141):

“*We emphasize that our while our biome delimitation differs from that of [12], the qualitative agreement (or disagreement) between the two is itself informative.”*

Second, and probably even more important -- it is hard to compare Jetz and Fine 2012 to the current analysis because the Skeels et al. paper includes many more biome-areas than we did.  This is problematic for a couple different reasons. First of all, in Jetz and Fine 2012 we made the case that we were trying to define the "evolutionary arenas" that influenced the speciation and extinction of all resident taxa and drove the regional patterns of diversity.  To justify these 32 "bioregions" we generated species lists of the vertebrate groups for each biome within continents (the bioregions -- see Figure 1 in Jetz and Fine 2012).  Importantly, we compared the betadiversity (Jaccard similarity) among bioregions to make sure that each biome area within continents represented an independent evolutionary unit.  Overlap in vertebrates among bioregions was 0% for all taxa except for birds (which was 4%).

The Skeels et al. paper includes 57 bioregions (listed in Figure 2).  Although the authors tell us that they made sure that each species in their analysis only was counted in one region -- they do not report what the % overlap is among their 57 bioregions.  I am guessing that the overlap would be substantial -- especially between tropical rainforest and tropical monsoonal forest, for example but probably within most of the Koppen biomes within climate zones.  Moreover, it is hard to even reconcile the bio-regions we included in Jetz and Fine 2012 with these Koppen biomes.   In Jetz and Fine, we lumped Tropical rainforest and seasonal moist forest to be one bioregion per continent (tropical moist forest) but separate from tropical dry forest and savanna.  This agreed with the species overlap data.  Here we have tropical rainforest separated from tropical monsoonal forest as well as tropical savanna.  Where is tropical dry forest?  Also, where are grasslands?

Would it be possible for the authors to lump the Koppen biomes into categories that more clearly match the 32 bioregions in Jetz and Fine?  It would be very interesting to see what the differences would be between the new paleoclimate maps and our hand-drawn maps -- but the current manuscript doesn't really let us compare because the number of bioregions is so different AND the paleoclimate reconstruction are different.   I also have a hard time being able to justify a time-area analysis on the 57 Koppen biomes due to the lack of continuity of each of those Koppen biomes over the past 50 million years and the lack of independence as "evolutionary arenas"  (i.e., I doubt that tropical rain forest and tropical monsoon forest were independently generating new taxa within continents). What does "endemic" even mean in the context of 57 Koppen biomes?  At the very least the authors should make it very clear that the comparison to Jetz and Fine 2012 by the current analysis is incomplete due to the different number and definition of a bioregion and the authors should report the % overlap in vertebrate taxa among all of their Koppen biomes.

We respond to the previous paragraphs together as they relate to a single question: how comparable are Jetz and Fine (2012) and our study?

The reviewer is correct in that the results cannot be compared unit-for-unit between the two studies because we use a specific definition based on threshold of climate in contrast with the ones used by Fine and colleagues in previous studies, that are based on the vegetation types and typically estimate paleobiomes from the presence of specific indicator taxa or functional traits in the fossil record.

We see the approaches as complementary, and they are in dialogue with each other, not in opposition. Our aim is not to replicate Jetz and Fine exactly but to extend that work using paleoclimate models that were not available in 2012. Therefore, we do not think a one-to-one replication is necessary for the comparison to be informative. We do agree that a stronger caveat is required so that readers can interpret our results with this in mind and we have added that in the aims section of the Introduction (L139-141) and again in the discussion (L320-323).

Some specific answers to questions raise:

1 **Analyses at a coarser, more directly comparable resolution.** Our original analysis was at the second-tier Koppen level with 57 realm-biome units. We cannot repeat the analysis on the exact same biomes as Jetz and Fine (2012) as the two biome concepts are not directly comparable. However, we did repeat the full analysis at the first-tier Köppen level (A–E), giving 26 realm–biome units across models, which is closer to the 32 bioregions of Jetz & Fine. This grouping also clumps biomes such as the tropical rainforest and monsoonal tropics under the same umbrella, as suggested by the reviewer.

As mentioned above, this analysis at the first-tier Koppen level, both with the climate-model derived reconstruction and the proxy-derived reconstruction, gave similar results to our fine-scale biome analysis. So while the differences in results may be attributed to different biome definitions which we flag in the main text (L139-141), we show it is not attributable to the concerned “over-splitting” of biomes in the second-tier Koppen classification. We added the following explanation, L251-257:

*“We performed this validation on realm-biome units of the primary Köppen category which was available both for proxy-based and climate model-based reconstructions (Table S6). We found that present-day area was a consistently better fit than proxy-based timeArea for all vertebrate classes and richness metrics, and climate model-based estimates of timeArea were a better fit than proxy-based estimates. This result also highlighted that the relationship was robust to the resolution of Köppen category.”*

2 **Species overlap among biomes.** We agree with the reviewer that reporting details of the vertebrate data is essential. We now report pairwise overlap among biomes explicitly in a new supplementary table (Table S8), methods (L538-542), and results L218-221. Following the same approach as Jetz and Fine (2012), we show that our realm-biome units also have very low levels of overlap, with values nearly identical values for ecotherms, and similar levels for endotherms albeit slightly lower for birds and slightly higher for mammals:

| Vertebrate class | Median Jaccard Overlap Our study | Median Jaccard Overlap Jetz and Fine, 2012 |
| --- | --- | --- |
| amphibians | 0.07 | 0 |
| reptiles | 0.11 | 0 |
| birds | 2.10 | 4 |
| mammals | 3.48 | 0 |

As such, our biome concept does reflect evolutionary arenas in the same way as Jetz and Fine’s and is not affected by the problem the reviewer proposes.

3 **Tropical dry forest and grasslands**: Not all biomes under the WWF scheme have exact analogues in the Koppen scheme, yet there are close comparisons. Tropical Dry Forests most directly correspond to the Tropical Seasonal biome (Aw), which has seasonal variation in rainfall. Grasslands map to several different Köppen levels depending on the overall temperature and precipitation regimes. Dry grasslands such as the Eurasian Steppe, Patagonian Steppe, Shortgrass North American prairie map to the Semi-Arid Steppe biome (Bsk). Tropical grasslands and savannas map to Tropical Seasonal (Aw).
4 **The meaning of Endemic in our study.** Endemic richness is the number of species whose entire range falls within a single realm–biome unit. This is the same definition used by Jetz & Fine (2012). At the second-tier Köppen level this comprises 3,170 amphibians (46.0% of the class), 3,736 squamates (44.5%), 1,040 mammals (18.9%) and 881 birds (9.4%). Endemism therefore remains substantial at this resolution, particularly in ectotherms. As noted above, we recover the same result at the primary Koppen (A–E) level where endemism is even higher - between 21% and 61% of each vertebrate class. We have included this information in Table S8.
5 **Continuity of biomes in time**. The reviewer argues that the Köppen biomes lack continuity through time and don’t represent evolutionary arenas.  We draw the attention of the reviewer to Figure S13-17 which illustrates continuity of biomes through time, and as explained above, overlap values of our biome classification scheme has near identical overlap values to those reported in Jetz and Fine (2012).

Finally, I find the whole equilibrium diversity and carrying capacity discussion to be overdone. You can have diversity tracking contemporary biome sizes due to short lag times how biome area influences speciation and extinction rates.  Please see pages 376-377 in Fine (2015) for a different perspective.

We frame the discussion in terms of equilibrium dynamics, as this was the hypothesis we set out originally to test and also to engage in discourse on evidence for the role of equilibrium dynamics with several recent studies, including Cerezar et al. (2026) in PLOS Biology. We do agree, however, that there are alternative explanations and we raise this now in the discussion L409-413:

*“More broadly, the trajectory effects we recover need not imply ecological carrying capacities and it is also possible that a short lag-time response of diversity to changes in biome area could produce the same signal (Fine 2015). In such instances we might additionally expect biome area and trajectory to be related to diversification rates, and this may be tested further using area-based diversification models.”*

Line 67 should read "distribution of biodiversity"

Fixed.

Line 86.  Authors should say here that these models disagree with known paleobotanical localities

The Scotese and Boucot proxy maps and point localities include palaeobotanical evidence, including mangroves and palms, so it would not be correct to claim these reconstructions disagree with paleobotanical studies.

line 113 should say "trees" instead of "plants"

Fixed

**-----------------------------------------------------------------------------------------------------------------------------------**

**Reviewer #3:** Dear Editor,

Skeels et al. have adequately resolved any of my previous concerns and I think their work now forms a valuable contribution to the broader field. My previous concerns primarily related to the framing of the results and the clarity of writing. Specifically, the discussion of ecological versus historical processes has been nuanced and the extended focus on biological interpretations of their results have improved the paper substantially. I have listed only a couple of minor points below that can easily be addressed.

We thank the reviewer for their time and have addressed each of the remaining minor comments.

L63-L64: "Ecological and historical explanations for diversity are not mutually exclusive, but sit along a continuum". Although I agree with the sentiment, I wasn't entirely sure about the phrasing here. Specifically, what sits along the continuum? Is it the explanations/hypotheses or is it the resulting diversity (e.g. X% caused by history and Y% by ecology?).

We apologize for the confusion. We meant that the speed of the response of biodiversity to change in climate or area sits along a continuum from rapid response (things track equilibrium) to slow response (historical effects matter more). We fixed this sentence to clarify this point L64-66: “Ecological and historical explanations for diversity are not mutually exclusive, but endpoints along a continuum, along which areas or clades are positioned by the speed at which their diversity responds to changes in area or energy relative to how fast these changes occur [11].”

L124: "at paint"

Fixed

Figure 1: Bit of a pet peeve, but why is HadCM3L-B followed by A and not the other way around? Additionally, is (B) 0 Ma the present or is it model output? If the former perhaps specify in the legend.

We have fixed this now.

L252: plural or singular model?

Plural. Fixed.

L350: citation?

We have added reference to work by Schluter, Rabosky on this topic.

L456: have a?

Fixed.
