# Supplementary material for: High-resolution paleobiome reconstructions highlight ecological and historical dynamics underpinning global terrestrial vertebrate diversity
Source: PLoS Biol. 2026 Oct 23;24(10):e3004077. doi: 10.1371/journal.pbio.3004077 (PMC13719045; doi:10.1371/journal.pbio.3004077)
Supplement: Supplementary file 3 [file pbio.3004077.s006.docx]

**Reviewer Comments**

Reviewer #1: I am happy with the current version of the manuscript, the authors have addressed all my (minor) remaining concerns, and as far as I can tell, also the concerns of the other two referees. In fact, I really appreciate the effort the authors put into addressing all these concerns; it is now very satisfactory.

**Response:** We are very glad to have satisfied the reviewer’s concerns and appreciate their time in reviewing our revision.

Reviewer #2: I'd like to thank the authors for their detailed and careful revisions to their manuscript and their point-by-point responses to my earlier comments.   I'd like to just make a few comments about their five responses where they describe their new analyses on first-tier Koppen biomes and report the overlap among these 26 units.

First, thanks for doing this and demonstrating that the proxies and models all are showing effectively the same pattern -- stronger signal from present-day area and energy in squamate diversity. I think that these first-tier analyses are the ones that should be used in the paper as they actually do represent "evolutionary arenas"  that is -- the biome units that represent regional diversity and derive from realm-specific processes of speciation and extinction (and dispersal).   The second-tier Koppen biomes do not represent evolutionary arenas -- and in point 4 the authors give us the figures of endemism which range from medium levels for amphibians and reptiles and low and very low for mammals and birds. As such, I don't think that these smaller units can be tracked through time as continuous units given that they include a species pool that includes more than one second-tier Koppen biome.

However, while I think that this is a super important point, I don't want to delay publication of this manuscript by insisting on new revisions.

Paul Fine

**Response:** We are glad the revised manuscript satisfied the reviewer’s comments and thank them genuinely for their time and help in improving our manuscript.

For the remaining point of Reviewer #2, given that the conclusions are the same whether using the first-tier or second-tier Koppen definitions, both are reported in the main text regardless, and that there is much to be gained from partitioning biomes with the finer-resolution, second-tier classification (e.g., distinguishing Tropical Savannas from Tropical Rainforests), we would prefer to keep the second-tier as the primary analysis featured in the main figures. We note that the Jaccard overlap values are similar between tiers and are very similar to the values provided by Jetz and Fine (2012) and therefore are independent evolutionary arenas, albeit at a smaller scale than the first-tier Koppen biomes.

~~~~~~~~~~~~~~~~~~~~~~~~~~~~~~~~~~~~~~~~~~~~~~~~~~~~~~~~~~~~~~~~~~~~~~~~~~~~~~~~

**Editor Comments**

a) We need numerical values underlying data on Fig1A, Fig1B, Fig2A, Fig3A,B and Figs S1-S17. I note that you have a related Zenodo repository (<https://aus01.safelinks.protection.outlook.com/?url=https%3A%2F%2Fdoi.org%2F10.5281%2Fzenodo.17595796&data=05%7C02%7Calexander.skeels%40anu.edu.au%7Cfa936a29dd7a4a3261bd08df20914e6f%7Ce37d725cab5c46249ae5f0533e486437%7C0%7C0%7C639265480639057602%7CUnknown%7CTWFpbGZsb3d8eyJFbXB0eU1hcGkiOnRydWUsIlYiOiIwLjAuMDAwMCIsIlAiOiJXaW4zMiIsIkFOIjoiTWFpbCIsIldUIjoyfQ%3D%3D%7C0%7C%7C%7C&sdata=LNyF0F2XUf2mrDkrf1NzKfStGhmwesK0dfyuZ4xIOP4%3D&reserved=0>); however, access is currently restricted, so I am unable to check your compliance with our data and code availability policies. Please make it publicly accessible so that I check your compliance with our policies.

**Response:** All numerical values unpinning the Figures are provided in the Zenodo, now made public at 10.5281/zenodo.17595795.

b) Please clearly cite the location of the data in all relevant main and supplementary figure legends. For example, you can add: “The data and code needed to generate this figure can be found at doi.org/10.xxx/zenodo.xxxxx.” We understand that this may appear repetitive, but it helps make the figures and their legends more self-contained.

**Response:** We have added the following statement to all relevant main and supplementary figures: “The data and code needed to generate this figure can be found at doi.org/10.5281/zenodo.17595795”

c) Please include the URLs of your funders in the Financial Disclosure statement.

**Response:** We have modified the Financial Disclosure Statement as follows: “This work was supported by the Australian Research Council (Discovery Early Career Research Award DE250100004 to AS; https://www.arc.gov.au/). The funders had no role in study design, data collection and analysis, decision to publish, or preparation of the manuscript.”

d) Please make any custom code available, either as a supplementary file or as part of your Zenodo deposition.

**Response:** We have uploaded custom code to reproduce the analyses and figures into the Zenodo supplement and updated the README to reflect this.
