# Supplementary material for: High-resolution paleobiome reconstructions highlight ecological and historical dynamics underpinning global terrestrial vertebrate diversity
Source: PLoS Biol. 2026 Oct 23;24(10):e3004077. doi: 10.1371/journal.pbio.3004077 (PMC13719045; doi:10.1371/journal.pbio.3004077)
Supplement: Supplementary file 4 [file pbio.3004077.s004.odt]

Reviewer #1: This is a well written and nicely presented manuscript that shows that species richness of terrestrial biomes is poorly explained by biome history, while current combination of area and productivity (and temperature) predicts species richness reasonably well. This is a convincing argument for equilibrium diversity dynamics with fast tracking of pronounced area changes (i.e. with relatively short lag of biodiversity dynamics behind environmental changes). I consider these results important and valuable, so that the study should be ultimately published, although I have several concerns that should be first addressed.

Response: We thank the reviewers for their time and have addressed each point below.

First, and most importantly, the whole study is based on a suite of models of past climate that have been used for the reconstruction of biome positions and areas during the Cenozoic. The authors themselves admit that the models, and thus the reconstructed biome history, largely differ from each other. It is thus natural to ask how reliable they are at all. I am aware that we do not have anything better, and I appreciate the clarity in which the authors present these conflicting results, but some evaluation of model performance, including model uncertainty, seems to be needed. As the authors admit, the models seem to conflict with classical views on the distribution of some (mostly tropical) biomes in distant past (especially the Eocene) which were based on proxy data, namely the presence of tropical elements (palms, crocodiles, specific tropical genera etc.) in high latitudes. This should be at least discussed a bit more. Even more importantly, if individual models provide so different outputs, how can we interpret the finding that the biome properties integrated through their history (namely area and productivity) do not explain richness variation? Could it be just due to insufficient ability of the models to capture the true history, i.e. due to an effective randomness of model outputs? Again, I understand we do not have better information about history, but some more discussion and/or model evaluation (e.g. by exploring how correlated are model outputs and what it means) is needed.

Response: We agree this is an important concern and discrepancies between proxy data and climate models, and among climate models, is a large and ongoing area of research. We have now included:
A quantitative assessment of congruence among climate models for the biome area trajectory and its effect on derived predictor variables of time-integrated area.
An in-depth validation of the model outputs against the proxy record using multiple approaches.

Climate model congruence

We re-classified biome trajectories using a stricter criterion (AIC > 2 over the null and slope p < 0.05). Under this criterion, 39 of 57 biome–realm combinations with a significant directional trend agreed across all five climate models (68.4%; main text 190-207; Fig 2; Figs S8–S12) — many of the ‘disagreements’ in the original submission were biomes with weak or non-directional trends. Pairwise correlations of biome-area trajectories (Table S2) confirm that HadCM3L-A and HadCM3L-B are nearly identical (r = 0.86), the remaining non-HadCM3L-C models agree moderately (mean r = 0.54), and HadCM3L-C’s lower agreement (mean r = 0.06–0.21) is driven by its different palaeotopography.

Predictor variable congruence

Despite some trajectory disagreement among models, time-integrated area and time-and-area-integrated productivity were highly correlated across all five climate models (mean pairwise r = 0.93; Table S3; main text L204–207), so trajectory differences had little effect on the integrated predictors used in the richness models.

Fossil and lithological indicator congruence

We added two new validation tests for our biome reconstructions against the climate proxy record from fossil and lithological indicators from existing global datasets. At a gird-cell level, based on expert-drawn maps of high-level Koppen biomes (Figure S6; Scotese et al. 2021) we compared the predicted biome classification from the proxy data to our reconstructed prediction of biome from the climate models and assessed congruence with the κ metric (Figure S7A). Our climate models showed substantial agreement with expert-drawn proxy reconstructions based on the κ metric of agreement in the past 25 Ma (κ = 0.58–0.70), declining to fair agreement in the Eocene–Palaeocene (κ = 0.25–0.38). We also compared our climate model reconstructions directly to the point localities of the proxy data from Boucot et al 2013 (Figure S7B; Table S1). We found that 71.6% of 1,381 lithological proxies were consistent with model predictions, compared with 81.4% for the expert-drawn maps. In other words, the climate models recover 78.9–92.1% of the proxy-map ceiling, and actually outperformed the expert maps for tropical lithologies. We have updated the methods and results to include this validation (Methods L473-490; Results L170-189)

Taken together, we now argue that the differences between the climates and the difference between the climate models and the proxy records are overstated in the original manuscript by focusing on areas with the most uncertainty (e.g., the Neotropics) and our new validation steps quantify this.

Boucot AJ, Xu C, Scotese CR, Morley RJ. Phanerozoic paleoclimate: an atlas of lithologic indicators of climate. Tulsa, Okla: SEPM (Society for Sedimentary Geology); 2013.
Scotese CR, Song H, Mills BJWW, van der Meer DG. Phanerozoic paleotemperatures: The earth’s changing climate during the last 540 million years. Earth-Science Reviews. 2021;215: 103503.

Second, besides testing the effects of time-integrated biome properties, the authors tested the effect of biome expansion or contraction on species richness. However, it seems to be done in a too simplified way, just using Kruskal-Wallis for comparison of species richness between these two groups. But biome richness is for sure affected by other factors, and these should be accounted for in a single model - i.e. not testing just the difference between the groups (expanding-shrinking-unchanging), but whether the fact that a biome expands or shrinks explains more richness variation than a model that includes only current area, productivity and temperature. Note that such test comprises something different than the other tests comprising the time-integrated properties: while the time-integration tests are focused on the role of time of species accumulation for species richness (together with niche conservatism etc.), i.e. essentially non-equilibrium processes, the effect of shrinkage/contraction is compatible with an equilibrium view and indicates whether there is a time lag in the equilibrium dynamics. It can be still there, regardless of the other results, if properly tested together with the other factors in a single model.

Response: We replaced the Kruskal–Wallis test with biome trajectory as a fixed-effect covariate in the GLMM model selection alongside area, prodArea, timeArea, timeProdArea, and temperature (Fig 3B–C; Table S5). Trajectory improved fit over the best present-day model in 3 of 12 cases (total richness for birds and squamates, endemic richness for mammals) and was a comparably-good covariate (∆AIC < 2) in a further 4 cases (amphibian total/resident, squamate resident/endemic). The biological interpretation of these trajectory effects is now in the discussion at L364-L392.

Third, although the overall theoretical framing is sound, it still should be improved at some places. For instance, the authors argue that large areas affect richness via promoting more opportunities for allopatric speciation (lines 80-81 and below), but this is not at all the only effect area can promote diversity - large areas also allow larger populations which are less prone to extinction or which speciate with higher probability for other reasons (even sympatrically) - see the cited references comprising the theories of equilibrium dynamics. Also, the equilibrium itself is not in stark contrast to historical legacy (as suggested by the sentence on lines 115-117) if there are some time lags (see the cited reference 64) which would be revealed by the role of recent biome expansion/shrinkage, as pointed out above.

Response: We have re-framed the introduction and discussion considerably around an ecological–historical continuum rather than an equilibrium versus non-equilibrium dichotomy. We explicitly include discussion of population-size and extinction pathways and a treatment of time lags (L318-363).

Fourth, the authors correctly contrast their findings with Jetz & Fine (2012), i.e. the paper which argued for the role of time-integrated biome (or ecoregion) properties. However, curiously, the fit of the models that included the time-integration was in Jetz & Fine (2012) better than models including just current area, productivity and temperature only in the case of endemic species, whereas it was almost equally good (as the alternative without time-integration) for ectotherm residents and even worse for endotherm residents (see their Table 1). Therefore, the current findings are not in such striking contrast with Jetz & Fine (2012), even though the interpretation differs. It is not a big issue, as the authors partly admit it on lines 258-259, but it could be a bit more stressed.

Response: We re-framed the comparison with Jetz & Fine in the discussion (L307-317): we agree that that study found prodArea+temperature to be the best model for resident endotherms which matched our finding for that group. We now stress that our analysis differs mainly for endemic species and resident ectotherms. A more nuanced discssuion for the role of history is now included throughout the discussion and we also focus more strongly on the main differences between the major vertebrate clades (e.g., Fig 3).

Concrete points related to particular lines:

Lines 55-58: The sentence is a bit vague and not entirely correct - what are "equilibrium and non-equilibrium factors"? Of course, the dynamics can have either equilibrium or non-equilibrium properties, but these kinds of dynamics are to a large extent exclusive (strictly speaking, equilibrium dynamics does not contain the information on history).

Response: This sentence has been removed in the re-framing of the introduction around an ecological–historical continuum.

Lines 61-62: Antarctic glaciation might not be caused just by the decrease of CO2 concentrations, as suggested by the text, but rather (or at least also) by tectonics, namely the split of Australia from the Antarctic, leading to a formation of circumpolar cold currents and consequent Antarctic glaciation.

Response: Added: the circumpolar current driven by South America/Australia–Antarctica separation is now cited alongside CO₂ decline as a driver of Antarctic glaciation.

Line 92: Which rates are expected to be slowing? Rates of extinction are predicted to be increasing when approaching equilibrium, at least relative to speciation rates.

Response: This sentence no longer appears in the introduction but we make sure to clarify that it is diversification rates that are slowing driven by increasing extinction rates and/or decreasing speciation rates.

Lines 208-213: This part is not entirely clear - how it relates to biome history?

Response: This passage was removed in the discussion restructure; the new discussion opens with biome interpretation followed by richness implications.

Lines 220-222 (and below): Indeed, there is this conflict between the estimates of the expansion or shrinkage of neotropical rainforests - so what does it mean? If the models are not reliable, how can be they used for the analyses presented here? This whole part of the paragraph (and partly also the paragraph which follows) does not provide a clear clue for a proper interpretation of the reconstructions, and thus of the results. It might be better (see also the general comment above) to devote a separate part of the text to the caveats comprising the reconstructions.

Response: We reduced the discussion of model disagreement and instead emphasize general consistency, with regional uncertainty (notably the Neotropics) framed against the literature (see response to reviewer 2 below).  The new validation analysis (Figs S6–S7) shows that overall the climate models perform well against the proxy record.

Line 255: delete "a" - "were consistently better predictors".

Response: Fixed.

Line 293: As mentioned above in the general comments, the legacy effect should be tested in a better way.

Response: Addressed via the new GLMM design that includes biome trajectory as a covariate (see response above)

Line 402: The Neogene is dated from 23 MYA, not 15 MYA!

Response: Fixed. We didn’t intend for this to suggest the start of the Neogene was 15 Ma. We chose 15 Ma to correspond with increasing global aridification and have clarified this in the methods section (L537-540).

Lines 414-415: Kruskal-Wallis, not "Kruskal Wallace". Also, the whole test should be much more sophisticated, as mentioned above. Even if it then turns out that there is an effect of expansion/shrinkage, it would not violate the equilibrium dynamics.

Response: The Kruskal–Wallis test has been removed in favour of including trajectory as a GLMM covariate.


Reviewer #2: I enjoyed reading the contribution by Skeels et al. The manuscript  revisits our 2012 PLOS Bio paper (Jetz and Fine), essentially asking the same questions we did but with new paleoclimate models: are current vertebrate diversity totals (endemic to a bioregion) better predicted by present-day area and energy or by past time-integrated area and energy? The new analysis reports that in contrast to our 2012 findings, present day area and energy explains vertebrate diversity better than metrics that incorporate time-area-energy.

Response: Thank you taking the time to review our manuscript, your own work was an inspiration for this study and so we are glad that you enjoyed reading it.

I don't doubt that there have been many advances in paleoclimatic modeling.  However, I question whether these current models can really considered improved if they contradict the paleobotanical record.  There have been many new findings in paleobotany that have informed our understanding of past biomes since our 2012 paper, summarized in Carlos Jaramillo's review (with Cardenas) in 2013 and also see Jaramillo 2023 cited by the authors.  In particular, Jaramillo and his team find evidence for large tropical rainforests in South America and very little evidence for arid biomes as predicted by the paleoclimatic models used by the authors. 

Response: We added a quantitative validation of all five models against (i) expert-drawn proxy-based reconstructions and (ii) 1,381 individual lithological proxy localities (Methods L473-490; Results L183-189; Figs S6–S7; Table S1). Models recovered 78.9–92.1% of the agreement achieved by the expert-drawn proxy maps themselves, and even outperformed the expert maps for tropical lithological indicators (laterites, bauxites, mangroves; Table S1). We also note that the regional uncertainty is consistent with the literature: Cárdenas & Jaramillo’s (2013) own Figure 4 (copied below) shows 12 published Eocene South American reconstructions ranging from continent-wide rainforest to extensive aridity. But we also add that a growing consensus predicts widespread rainforest. We retain the discussion of model uncertainty in the discussion section, but we now also give greater attention to highlighting model congruence and agreement with the proxy record.


What would be a real advance is a paleoclimatic model that could include a "fossil calibration" that could include micro and macrofossil locations in time and constrain the paleoclimatic model and make it agree with the fossil evidence.   Instead, we get a re-analysis with a new model but not necessarily any confidence that it is better than our hand-drawn maps using the few best available sources in 2012.  I think the authors should redo their analysis with their advanced paleoclimatic models that also includes the best known paleobotanical data. 

Response: Building a fossil-calibrated GCM is beyond this paper’s scope, but we have done the next-best thing: a cell-by-cell comparison against expert-drawn proxy-based Köppen reconstructions (Scotese et al. 2021; Fig S6) and a locality-by-locality comparison against the Boucot et al. (2013) lithological proxy database (Methods L473-490; Results L183-189; Fig S7; Table S1). These confirm that the climate models hold up well against the proxy record.

Another factor that the authors could consider is climatic stability.  Tropical areas likely experienced much less dynamism of time-area-energy than high latitude areas.  In our 2008 paper (Fine, Ree, and Burnham 2008, in Tropical Forest Community Ecology, Carson and Schnitzer eds, see Figure 3.5 on page 40) we plotted minimum area of biome based on estimates from the last glacial maximum.  I think the authors could consider a similar approach with their paleoclimatic modeling during the Pleistocene to see whether current diversity is correlated with minimum area size (and that extinction during this time would be a big factor)

Response: This is an interesting point. We added a minimum-biome-area test to match the analysis in Fine, Ree & Burnham 2008 (now cited at ref 32). For each realm-biome-climate-model combination we extracted the minimum biome area across all Cenozoic time steps and compared its fit to present-day area. Present-day area outperformed minimum area in 11 of 12 vertebrate class × richness metric comparisons; the one exception was an equally-good fit (∆AIC < 2) for amphibian endemic richness under HadCM3L-B. Results at L247-252; full results in Table S7.

Finally, I think that the authors make a false dichotomy with their interpretation of their results as it regards equilibrium and non-equilibrium explanations of diversity patterns. If they read Jetz and Fine 2012 or Fine 2015, they will see that time-area-energy and the tropical conservatism hypothesis is hypothesized to be important because of the effect of area and energy integrated over time and this effect on population sizes and speciation and extinction probabilities, not simply speciation probabilities as described in the text (see line 85). In other words, integrated time-area-energy nor tropical conservatism hypothesis are "non-equilibrium" hypotheses. There is a lot more nuance.  For a full discussion on this issue, see Fine (2015) pp. 373-377.

Response: This is a fair point, and one also mirrored by other reviewers. We have now re-framed both the introduction and discussion to treat ecological and historical processes as a continuum modulated by the speed of biodiversity response and lag. We explicitly avoid using the equilibrium/non-equilibrium dichotomy but we do discuss our results in the context of equilibrium biodiversity theory (L307-363).


Reviewer #3: 

Skeels et al. provide an interesting study in which they follow up on the paper published by Jetz & Fine (2012) published in Plos Biology. Building upon their framework, they use improved paleobiome reconstructions to revisit the question if historical biome area has a strong effect on contemporary species richness patterns, or if this is largely determined by present-day area and energy availability. In contrast to Jetz & Fine, they find that past biome area does not explain additional variance in diversity. The paper is clearly written, and the use of state-of-the-art climate reconstructions is timely and a valuable endeavour. I do not have major methodological concerns.

Response: We thank the reviewer for their time in reviewing our manuscript, we are glad they found it a valuable contribution.

Saying that, I primarily think the paper can be improved in terms of (i) framing and justification of hypotheses, (ii) improved upfront discussion of the methodology to improve flow, (iii) additional discussion of analyses focusing on separate vertebrate groups, and (iv) improved discussion of the interpretation of the results and contrasting finding (and cutting down the discussion on paleobiomes).

(i) I'm not convinced yet that the authors can truly disentangle the equilibrium and non-equilibrium hypotheses that they propose. For instance, (see my comment on L84-85 also) does extinction only increase under ecological limits when habitat contracts? One could assume that if species rapidly move without constraint they can all accumulate in the tropics upon contraction of the tropical biome, but even without ecological limits it is unrealistic to think that all species could make it in time, especially under rapid climate change. Additionally, I'm unsure how major barriers are accounted for. If say, Antarctica was tropical, would all those species be able to make it to the Amazon? That diversity may decrease upon biome area decrease under both hypotheses is important, because it makes it unclear how the two scenarios are distinguished based on the available data - without a temporal perspective in rate or diversity.


Overall, I think that the distinction between equilibrium and non-equilibrium processes can be more nuanced than the dichotomy portrayed by the authors, and I think the paper would benefit from a broader discussion of studies investigating the impact of history on diversity (as mentioned below).

Response: This is a fair point and part of our decision to model the change in biome area in each realm separately as we do not assume that contraction of a tropical biome in Antarctica would have any effect on the diversity of a tropical biome in the Amazon. We also dropped the equilibrium/non-equilibrium dichotomy in favour of a focus on the continuum of ecological and historical processes in the signature of biodiversity patterns. We also now discuss expected lags in the response of diversity driven by the speed of environmental change rather than diversification-rate signatures specifically (L345-363).

Ideally, a spatial simulation (e.g. with gen3sis) portraying the two hypotheses may be most convincing and add additional novelty. However, I realise that it would require a lot of work.

Response: This is something the authors are actively working on, but this represents a huge amount of work and is unfortunately well beyond the scope of this study. We highlight this as an avenue of future research in the conclusion.

(ii) in various cases, as the comments below detail, terminology is used or results are described with no lead up. This makes it difficult to understand what has been done and why.

Response: Each analysis is now foreshadowed in the introduction (study aims at L130-143) and each Methods subsection opens with a purpose sentence.

(iii) While mentioned in a figure and single sentence, differences (or rather the absence of) are not discussed.

Response: All analyses are now run per vertebrate class (birds, mammals, amphibians, squamates), with class-specific results (Fig 3) and class-specific biological interpretation in the discussion. Interestingly, we find that, while birds, mammals, and amphibians showed similar trends in the results, squamate reptiles showed slightly different effect of productivity and historical direction of biome growth (trajectory).

(iv) Most important, the former is a symptom of the lack of discussion of biological implications of the results and discussion in relation to other studies that studied the effect of history on species diversity patterns. A lot of the discussion (all except two paragraphs) deals primarily with the biomes. While interesting to a degree, I'm not sure that this should be the focus in a biology paper. In contrast, the authors could explore what their results mean in relation to patterns of diversification rates (while acknowledged it is not mentioned that much work already exists) and other historical influences in contemporary diversity patterns, e.g. out-of-the-tropics (Jablonski et al. 2006), higher diversification rates towards higher latitude (Rabosky et al. 2018; Weir & Schluter 2007), time-for-speciation (Stephens & Wiens 2003), and/or signatures of history in contemporary species ranges (Pigot & Tobias 2013; Reijenga et al. 2025). In general, it would thus be interesting to read how, in the authors view, (tropical) diversity originated and what the nuanced role of history is in contemporary patterns.

Response: We originally had four paragraphs dedicated to the nuances of the biome reconstructions which distracted from the main biological results. We have now reduced the biome section to three smaller paragraphs. Although we cannot extend the discussion to mention all of the topics suggested here we have extended the biological part of the discussion to focus on the difference among vertebrate clades, high latitude diversification rates, and time-for-speciation effects, as well as providing a deeper synthesis of the origins of biodiversity gradients and the role for history within a broadly ecological equilibrium dynamic (L307-392).

33-36: Quite a lot of information for the abstract. Consider condensing.

Response: We have now simplified this sentence.

36: Unclear to me what biome trajectories are. I assume that it means the total area, but consider expanding on it slightly.

Response: Defined now in the abstract and main text (133-134) as the direction of change in biome area over time, classified as expansion, contraction, or no significant trend.

84-85: I don't think I agree with this statement. If we envision a world in which species are constrained in their climatic niche and have limited dispersal ability, a contraction in available habitat may result in increased extinction because the species is not able to move in time regardless of the presence of ecological limits. This is frequently assumed in many studies employing SDMs or niche models and thus used for projections of the impact of climate change in the future.

Response: Agreed, we have softened this point. We have removed this part from the introduction altogether, with our new reframing. We have also made the population-size / extinction pathway explicit in the discussion (L325-332).

This additionally opens up the question that the rate of contraction/expansion may be important for changes in diversity.

Response: Yes great point. The rate-of-change point is now central to the treatment of “lags” in the response of biodiversity to climate change. We raise this in the introduction (L104-108) and discussion (L336-363), where we discuss how the speed of biome change relative to diversification times produces detectable historical signatures.

94-95: Would predictions not differ if there were recent expansions of biome area? i.e. suddenly there is more habitat which may only gradually cause higher richness build up through speciation? 

Response: Yes, we explicitly foreshadow this in the introduction and tested via the trajectory covariate.

105-106: Is there an example of how they differ? Is it general accuracy and spatial resolution or have notable events changed or temporal trends changed?

Response: Expanded at introduction L124-129: climate models add (i) spatial continuity over the globe at regular time intervals where proxy data are sparse, and (ii) the resolution to distinguish secondary Köppen sub-types (e.g., Cf vs Cs vs Cw) rather than only primary categories (A–E).

107-109: From the introduction it is unclear to me what the diversity date is that the authors are trying to understand. What was this based on (e.g. range maps), how was it compiled, what groups does it contain, etc. Helping the reader now with a slightly expanded description of the methods will help the interpretation of the results.

Response: The final intro paragraph (L130-143) now names the data: terrestrial vertebrate classes (birds, mammals, amphibians, squamates) with data source availability mentioned in the Methods.

"In general, I'm a little confused that all vertebrates seem to be lumped together in one analysis. Jetz and Fine also showed how it differed between vertebrate groups and between ecto- and endotherms. Given that they differ widely in their ecology and dispersal ability, historical factors might have different effects." Was my initial thought until I finally spotted the analysis in Figure 2B (comment below) - I'll leave this here as I still agree with this sentiment because it was difficult to find the results and they are not discussed still in much detail.
155: The first time that it is mentioned that multiple vertebrate classes are studied, but not mentioned again? What is the interpretation of these results?

Response: Vertebrate classes are now introduced at L116–128, analysed separately throughout, and discussed class-by-class with two new dedicated paragraphs.

170: Why the past 35 Ma and not the entire Cenozoic?

Response:  We compared the fit of different time windows and found the 35 Ma window was best for total richness and the 15 Ma window for resident/endemic. The choice is explained in the Methods (L537-540).

172-174: What test are the correlations based on?

Response: Pairwise Pearson correlations; the section the reviewer was asking about has been replaced with a formal correlation analysis reported in Tables S2–S3.

249-252: Although an important message, do the authors require four paragraphs to discuss how biomes have changed through time?

Response: Reduced from four to three paragraphs.

256-258: The sentence is a bit difficult to parse. Consider rephrasing to make it clearer what Jetz and Fine found versus your work.

Response: We have rewritten this entire paragraph to better explain the differences and similarities between our study and Jetz and Fine (2012). See response to Reviewer 1.

Additionally, the authors here acknowledge the difference between ecto- and endotherms, but was any such analysis performed in the current study to establish any contradictions or similarities?

Response: Every analysis is now run separately for each vertebrate class (birds, mammals, amphibians, squamates), so endotherm vs ectotherm contrasts emerge directly. 

265-269: This is again a paragraph that discusses the biomes and not the implications of the results.

Response:  This has now been addressed in the restructuring of the discussion and trimming of the biome discussion section.

355: "areal"

Response: Fixed 

357: Which realms and how were they divided? Many methods exist that can be used to delineate them and different data sources. Is this interchangeably used with biome?

Response: Clarified: biogeographic realms are taken from the standard six-realm scheme and now shown in Figure 2.

387-388: How many of how many total species?

Response: We have added the per-class species totals in the methods: 9330 birds, 5515 mammals, 6898 amphibians, 8389 squamates.

374-382: with the information provided it is unclear to me what the phylogenies were used for.

Response: The phylogenies were not used in this study. This has been removed from the main text.

400-402: I finally get where that 35 Ma comes from (L170 comments). Saying that, what is the justification for this? As far as I know, the Neogene started ~23 Ma and not 15 Ma, and the Oligocene ~34 Ma. The temporal resolution may play a role here but that wouldn't explain the Neogene.

Response: The minimum resolution is 5 Ma in our datasets so we selected time steps that broadly correspond with major climatic events. Please see response to Reviewer 2 above.

Are the geological time scales properly matched up between the different climate models?

Response: Yes, Table 1 gives each model’s native temporal resolution (5 Ma for HadCM3L-A/B and PlaSim, 10 Ma for CESM, geological-stage for HadCM3L-C). For trajectory comparison we interpolate to a common 5 Ma grid. For time-integrated predictors we use each model’s native time steps within the 65/35/15 Ma window. The validation against proxy maps is performed at shared 5 Myr time steps from 5–65 Ma. The methods now directly clarify these points.

Jablonski, D., Roy, K. & Valentine, J.W. (2006). Out of the tropics: Evolutionary dynamics of the latitudinal diversity gradient. Science, 314, 102-106.
Pigot, A.L. & Tobias, J.A. (2013). Species interactions constrain geographic range expansion over evolutionary time. Ecology Letters, 16, 330-338.
Rabosky, D.L., Chang, J., Title, P.O., Cowman, P.F., Sallan, L., Friedman, M., et al. (2018). An inverse latitudinal gradient in speciation rate for marine fishes. Nature, 559, 392-395.
Reijenga, B.R., Etienne, R.S., Murrell, D.J. & Pigot, A.L. (2025). Speciation history shapes patterns of assemblage species richness in birds. bioRxiv
Stephens, P.R. & Wiens, J.J. (2003). Explaining species richness from continents to communities: The time-for-speciation effect in emydid turtles. American Naturalist, 161, 112-128.
Weir, J.T. & Schluter, D. (2007). The latitudinal gradient in recent speciation and extinction rates of birds and mammals. Science, 315, 1574-1576.
