# Supplementary material for: High-resolution paleobiome reconstructions highlight ecological and historical dynamics underpinning global terrestrial vertebrate diversity
Source: PLoS Biol. 2026 Oct 23;24(10):e3004077. doi: 10.1371/journal.pbio.3004077 (PMC13719045; doi:10.1371/journal.pbio.3004077)
Supplement: Peer review history

# Editor report: Decision Letter 0

**DOI:** 10.1371/journal.pbio.3004077.r001

**Authors:** Ankiit Ahluwalia

14 Jan 2026

Dear Dr Skeels,

Thank you for submitting your manuscript entitled "High-resolution paleobiome reconstructions highlight ecological dynamics underpinning global terrestrial vertebrate diversity" for consideration as a Research Article by PLOS Biology.

Your manuscript has now been evaluated by the PLOS Biology editorial staff (as well as by an academic editor with relevant expertise) and I am writing to let you know that we would like to send your submission out for external peer review.

However, before we can send your manuscript to reviewers, we need you to complete your submission by providing the metadata that is required for full assessment. To this end, please login to Editorial Manager where you will find the paper in the 'Submissions Needing Revisions' folder on your homepage. Please click 'Revise Submission' from the Action Links and complete all additional questions in the submission questionnaire.

Once your full submission is complete, your paper will undergo a series of checks in preparation for peer review. After your manuscript has passed the checks it will be sent out for review. To provide the metadata for your submission, please Login to Editorial Manager (https://www.editorialmanager.com/pbiology) within two working days, i.e. by Jan 16 2026 11:59PM.

If your manuscript has been previously peer-reviewed at another journal, PLOS Biology is willing to work with those reviews in order to avoid re-starting the process. Submission of the previous reviews is entirely optional and our ability to use them effectively will depend on the willingness of the previous journal to confirm the content of the reports and share the reviewer identities. Please note that we reserve the right to invite additional reviewers if we consider that additional/independent reviewers are needed, although we aim to avoid this as far as possible. In our experience, working with previous reviews does save time.

If you would like us to consider previous reviewer reports, please edit your cover letter to let us know and include the name of the journal where the work was previously considered and the manuscript ID it was given. In addition, please upload a response to the reviews as a 'Prior Peer Review' file type, which should include the reports in full and a point-by-point reply detailing how you have or plan to address the reviewers' concerns.

During the process of completing your manuscript submission, you will be invited to opt-in to posting your pre-review manuscript as a bioRxiv preprint. Visit http://journals.plos.org/plosbiology/s/preprints for full details. If you consent to posting your current manuscript as a preprint, please upload a single Preprint PDF.

Feel free to email us at plosbiology@plos.org if you have any queries relating to your submission.

Kind regards,

Ankiit

Ankiit Ahluwalia,

PLOS Biology

aahluwalia@plos.org

# Peer review: Decision Letter 1

**DOI:** 10.1371/journal.pbio.3004077.r002

**Authors:** Ankiit Ahluwalia

23 Mar 2026

Dear Dr Skeels,

Thank you for your patience while your manuscript "High-resolution paleobiome reconstructions highlight ecological dynamics underpinning global terrestrial vertebrate diversity" was peer-reviewed at PLOS Biology. It has now been evaluated by the PLOS Biology editors, an Academic Editor with relevant expertise, and by several independent reviewers.

In light of the reviews, which you will find at the end of this email, we would like to invite you to revise the work to thoroughly address the reviewers' reports.

As you will see below, the reviewers find the study potentially interesting, however they also raise a number of concerns and they offer suggestions to strengthen the study further. We think the reviewer comments will need to be thoroughly addressed, including reassessing models, interpretation of results and reframing suggestions that need to be addressed, before we can consider your study for publication.

After discussing these comments with the Academic Editor and the rest of the team, we have decided to invite a Major revision that should address all the points raised by the reviewers.

Given the extent of revision needed, we cannot make a decision about publication until we have seen the revised manuscript and your response to the reviewers' comments. Your revised manuscript is likely to be sent for further evaluation by all or a subset of the reviewers.

In addition to these revisions, you will need to complete some formatting changes, which you will receive in a follow up email. A member of our team will be in touch with a set of requests shortly.

We expect to receive your revised manuscript within 3 months. Please email us (plosbiology@plos.org) if you have any questions or concerns, or would like to request an extension.

At this stage, your manuscript remains formally under active consideration at our journal; please notify us by email if you do not intend to submit a revision so that we may withdraw it.

**IMPORTANT - SUBMITTING YOUR REVISION**

Your revisions should address the specific points made by each reviewer. Please submit the following files along with your revised manuscript:

1. A 'Response to Reviewers' file - this should detail your responses to the editorial requests, present a point-by-point response to all of the reviewers' comments, and indicate the changes made to the manuscript.

*NOTE: In your point-by-point response to the reviewers, please provide the full context of each review. Do not selectively quote paragraphs or sentences to reply to. The entire set of reviewer comments should be present in full and each specific point should be responded to individually, point by point.

You should also cite any additional relevant literature that has been published since the original submission and mention any additional citations in your response.

2. In addition to a clean copy of the manuscript, please also upload a 'track-changes' version of your manuscript that specifies the edits made. This should be uploaded as a "Revised Article with Changes Highlighted" file type.

*Re-submission Checklist*

When you are ready to resubmit your revised manuscript, please refer to this re-submission checklist: https://plos.io/Biology_Checklist

To submit a revised version of your manuscript, please go to https://www.editorialmanager.com/pbiology/ and log in as an Author. Click the link labelled 'Submissions Needing Revision' where you will find your submission record.

Please make sure to read the following important policies and guidelines while preparing your revision:

*Published Peer Review*

Please note while forming your response, if your article is accepted, you may have the opportunity to make the peer review history publicly available. The record will include editor decision letters (with reviews) and your responses to reviewer comments. If eligible, we will contact you to opt in or out. Please see here for more details:

https://blogs.plos.org/plos/2019/05/plos-journals-now-open-for-published-peer-review/

*PLOS Data Policy*

Please note that as a condition of publication PLOS' data policy (http://journals.plos.org/plosbiology/s/data-availability) requires that you make available all data used to draw the conclusions arrived at in your manuscript. If you have not already done so, you must include any data used in your manuscript either in appropriate repositories, within the body of the manuscript, or as supporting information (N.B. this includes any numerical values that were used to generate graphs, histograms etc.). For an example see here: http://www.plosbiology.org/article/info%3Adoi%2F10.1371%2Fjournal.pbio.1001908#s5

*Blot and Gel Data Policy*

We require the original, uncropped and minimally adjusted images supporting all blot and gel results reported in an article's figures or Supporting Information files. We will require these files before a manuscript can be accepted so please prepare them now, if you have not already uploaded them. Please carefully read our guidelines for how to prepare and upload this data: https://journals.plos.org/plosbiology/s/figures#loc-blot-and-gel-reporting-requirements

*Protocols deposition*

To enhance the reproducibility of your results, we recommend that if applicable you deposit your laboratory protocols in protocols.io, where a protocol can be assigned its own identifier (DOI) such that it can be cited independently in the future. Additionally, PLOS ONE offers an option for publishing peer-reviewed Lab Protocol articles, which describe protocols hosted on protocols.io. Read more information on sharing protocols at https://plos.org/protocols?utm_medium=editorial-email&utm_source=authorletters&utm_campaign=protocols

Thank you again for your submission to our journal. We hope that our editorial process has been constructive thus far, and we welcome your feedback at any time. Please don't hesitate to contact us if you have any questions or comments.

Sincerely,

Ankiit

Ankiit Ahluwalia,

PLOS Biology

aahluwalia@plos.org

------------------------------------

REVIEWS:

Reviewer #1: This is a well written and nicely presented manuscript that shows that species richness of terrestrial biomes is poorly explained by biome history, while current combination of area and productivity (and temperature) predicts species richness reasonably well. This is a convincing argument for equilibrium diversity dynamics with fast tracking of pronounced area changes (i.e. with relatively short lag of biodiversity dynamics behind environmental changes). I consider these results important and valuable, so that the study should be ultimately published, although I have several concerns that should be first addressed.

First, and most importantly, the whole study is based on a suite of models of past climate that have been used for the reconstruction of biome positions and areas during the Cenozoic. The authors themselves admit that the models, and thus the reconstructed biome history, largely differ from each other. It is thus natural to ask how reliable they are at all. I am aware that we do not have anything better, and I appreciate the clarity in which the authors present these conflicting results, but some evaluation of model performance, including model uncertainty, seems to be needed. As the authors admit, the models seem to conflict with classical views on the distribution of some (mostly tropical) biomes in distant past (especially the Eocene) which were based on proxy data, namely the presence of tropical elements (palms, crocodiles, specific tropical genera etc.) in high latitudes. This should be at least discussed a bit more. Even more importantly, if individual models provide so different outputs, how can we interpret the finding that the biome properties integrated through their history (namely area and productivity) do not explain richness variation? Could it be just due to insufficient ability of the models to capture the true history, i.e. due to an effective randomness of model outputs? Again, I understand we do not have better information about history, but some more discussion and/or model evaluation (e.g. by exploring how correlated are model outputs and what it means) is needed.

Second, besides testing the effects of time-integrated biome properties, the authors tested the effect of biome expansion or contraction on species richness. However, it seems to be done in a too simplified way, just using Kruskal-Wallis for comparison of species richness between these two groups. But biome richness is for sure affected by other factors, and these should be accounted for in a single model - i.e. not testing just the difference between the groups (expanding-shrinking-unchanging), but whether the fact that a biome expands or shrinks explains more richness variation than a model that includes only current area, productivity and temperature. Note that such test comprises something different than the other tests comprising the time-integrated properties: while the time-integration tests are focused on the role of time of species accumulation for species richness (together with niche conservatism etc.), i.e. essentially non-equilibrium processes, the effect of shrinkage/contraction is compatible with an equilibrium view and indicates whether there is a time lag in the equilibrium dynamics. It can be still there, regardless of the other results, if properly tested together with the other factors in a single model.

Third, although the overall theoretical framing is sound, it still should be improved at some places. For instance, the authors argue that large areas affect richness via promoting more opportunities for allopatric speciation (lines 80-81 and below), but this is not at all the only effect area can promote diversity - large areas also allow larger populations which are less prone to extinction or which speciate with higher probability for other reasons (even sympatrically) - see the cited references comprising the theories of equilibrium dynamics. Also, the equilibrium itself is not in stark contrast to historical legacy (as suggested by the sentence on lines 115-117) if there are some time lags (see the cited reference 64) which would be revealed by the role of recent biome expansion/shrinkage, as pointed out above.

Fourth, the authors correctly contrast their findings with Jetz & Fine (2012), i.e. the paper which argued for the role of time-integrated biome (or ecoregion) properties. However, curiously, the fit of the models that included the time-integration was in Jetz & Fine (2012) better than models including just current area, productivity and temperature only in the case of endemic species, whereas it was almost equally good (as the alternative without time-integration) for ectotherm residents and even worse for endotherm residents (see their Table 1). Therefore, the current findings are not in such striking contrast with Jetz & Fine (2012), even though the interpretation differs. It is not a big issue, as the authors partly admit it on lines 258-259, but it could be a bit more stressed.

Concrete points related to particular lines:

Lines 55-58: The sentence is a bit vague and not entirely correct - what are "equilibrium and non-equilibrium factors"? Of course, the dynamics can have either equilibrium or non-equilibrium properties, but these kinds of dynamics are to a large extent exclusive (strictly speaking, equilibrium dynamics does not contain the information on history).

Lines 61-62: Antarctic glaciation might not be caused just by the decrease of CO2 concentrations, as suggested by the text, but rather (or at least also) by tectonics, namely the split of Australia from the Antarctic, leading to a formation of circumpolar cold currents and consequent Antarctic glaciation.

Line 92: Which rates are expected to be slowing? Rates of extinction are predicted to be increasing when approaching equilibrium, at least relative to speciation rates.

Lines 208-213: This part is not entirely clear - how it relates to biome history?

Lines 220-222 (and below): Indeed, there is this conflict between the estimates of the expansion or shrinkage of neotropical rainforests - so what does it mean? If the models are not reliable, how can be they used for the analyses presented here? This whole part of the paragraph (and partly also the paragraph which follows) does not provide a clear clue for a proper interpretation of the reconstructions, and thus of the results. It might be better (see also the general comment above) to devote a separate part of the text to the caveats comprising the reconstructions.

Line 255: delete "a" - "were consistently better predictors".

Line 293: As mentioned above in the general comments, the legacy effect should be tested in a better way.

Line 402: The Neogene is dated from 23 MYA, not 15 MYA!

Lines 414-415: Kruskal-Wallis, not "Kruskal Wallace". Also, the whole test should be much more sophisticated, as mentioned above. Even if it then turns out that there is an effect of expansion/shrinkage, it would not violate the equilibrium dynamics.

Reviewer #2: I enjoyed reading the contribution by Skeels et al. The manuscript revisits our 2012 PLOS Bio paper (Jetz and Fine), essentially asking the same questions we did but with new paleoclimate models: are current vertebrate diversity totals (endemic to a bioregion) better predicted by present-day area and energy or by past time-integrated area and energy? The new analysis reports that in contrast to our 2012 findings, present day area and energy explains vertebrate diversity better than metrics that incorporate time-area-energy.

I don't doubt that there have been many advances in paleoclimatic modeling. However, I question whether these current models can really considered improved if they contradict the paleobotanical record. There have been many new findings in paleobotany that have informed our understanding of past biomes since our 2012 paper, summarized in Carlos Jaramillo's review (with Cardenas) in 2013 and also see Jaramillo 2023 cited by the authors. In particular, Jaramillo and his team find evidence for large tropical rainforests in South America and very little evidence for arid biomes as predicted by the paleoclimatic models used by the authors.

What would be a real advance is a paleoclimatic model that could include a "fossil calibration" that could include micro and macrofossil locations in time and constrain the paleoclimatic model and make it agree with the fossil evidence. Instead, we get a re-analysis with a new model but not necessarily any confidence that it is better than our hand-drawn maps using the few best available sources in 2012. I think the authors should redo their analysis with their advanced paleoclimatic models that also includes the best known paleobotanical data.

Another factor that the authors could consider is climatic stability. Tropical areas likely experienced much less dynamism of time-area-energy than high latitude areas. In our 2008 paper (Fine, Ree, and Burnham 2008, in Tropical Forest Community Ecology, Carson and Schnitzer eds, see Figure 3.5 on page 40) we plotted minimum area of biome based on estimates from the last glacial maximum. I think the authors could consider a similar approach with their paleoclimatic modeling during the Pleistocene to see whether current diversity is correlated with minimum area size (and that extinction during this time would be a big factor)

Finally, I think that the authors make a false dichotomy with their interpretation of their results as it regards equilibrium and non-equilibrium explanations of diversity patterns. If they read Jetz and Fine 2012 or Fine 2015, they will see that time-area-energy and the tropical conservatism hypothesis is hypothesized to be important because of the effect of area and energy integrated over time and this effect on population sizes and speciation and extinction probabilities, not simply speciation probabilities as described in the text (see line 85). In other words, integrated time-area-energy nor tropical conservatism hypothesis are "non-equilibrium" hypotheses. There is a lot more nuance. For a full discussion on this issue, see Fine (2015) pp. 373-377.

Reviewer #3:

Skeels et al. provide an interesting study in which they follow up on the paper published by Jetz & Fine (2012) published in Plos Biology. Building upon their framework, they use improved paleobiome reconstructions to revisit the question if historical biome area has a strong effect on contemporary species richness patterns, or if this is largely determined by present-day area and energy availability. In contrast to Jetz & Fine, they find that past biome area does not explain additional variance in diversity. The paper is clearly written, and the use of state-of-the-art climate reconstructions is timely and a valuable endeavour. I do not have major methodological concerns.

Saying that, I primarily think the paper can be improved in terms of (i) framing and justification of hypotheses, (ii) improved upfront discussion of the methodology to improve flow, (iii) additional discussion of analyses focusing on separate vertebrate groups, and (iv) improved discussion of the interpretation of the results and contrasting finding (and cutting down the discussion on paleobiomes).

(i) I'm not convinced yet that the authors can truly disentangle the equilibrium and non-equilibrium hypotheses that they propose. For instance, (see my comment on L84-85 also) does extinction only increase under ecological limits when habitat contracts? One could assume that if species rapidly move without constraint they can all accumulate in the tropics upon contraction of the tropical biome, but even without ecological limits it is unrealistic to think that all species could make it in time, especially under rapid climate change. Additionally, I'm unsure how major barriers are accounted for. If say, Antarctica was tropical, would all those species be able to make it to the Amazon? That diversity may decrease upon biome area decrease under both hypotheses is important, because it makes it unclear how the two scenarios are distinguished based on the available data - without a temporal perspective in rate or diversity.

Overall, I think that the distinction between equilibrium and non-equilibrium processes can be more nuanced than the dichotomy portrayed by the authors, and I think the paper would benefit from a broader discussion of studies investigating the impact of history on diversity (as mentioned below).

Ideally, a spatial simulation (e.g. with gen3sis) portraying the two hypotheses may be most convincing and add additional novelty. However, I realise that it would require a lot of work.

(ii) in various cases, as the comments below detail, terminology is used or results are described with no lead up. This makes it difficult to understand what has been done and why.

(iii) While mentioned in a figure and single sentence, differences (or rather the absence of) are not discussed.

(iv) Most important, the former is a symptom of the lack of discussion of biological implications of the results and discussion in relation to other studies that studied the effect of history on species diversity patterns. A lot of the discussion (all except two paragraphs) deals primarily with the biomes. While interesting to a degree, I'm not sure that this should be the focus in a biology paper. In contrast, the authors could explore what their results mean in relation to patterns of diversification rates (while acknowledged it is not mentioned that much work already exists) and other historical influences in contemporary diversity patterns, e.g. out-of-the-tropics (Jablonski et al. 2006), higher diversification rates towards higher latitude (Rabosky et al. 2018; Weir & Schluter 2007), time-for-speciation (Stephens & Wiens 2003), and/or signatures of history in contemporary species ranges (Pigot & Tobias 2013; Reijenga et al. 2025). In general, it would thus be interesting to read how, in the authors view, (tropical) diversity originated and what the nuanced role of history is in contemporary patterns.

33-36: Quite a lot of information for the abstract. Consider condensing.

36: Unclear to me what biome trajectories are. I assume that it means the total area, but consider expanding on it slightly.

84-85: I don't think I agree with this statement. If we envision a world in which species are constrained in their climatic niche and have limited dispersal ability, a contraction in available habitat may result in increased extinction because the species is not able to move in time regardless of the presence of ecological limits. This is frequently assumed in many studies employing SDMs or niche models and thus used for projections of the impact of climate change in the future.

This additionally opens up the question that the rate of contraction/expansion may be important for changes in diversity.

94-95: Would predictions not differ if there were recent expansions of biome area? i.e. suddenly there is more habitat which may only gradually cause higher richness build up through speciation?

105-106: Is there an example of how they differ? Is it general accuracy and spatial resolution or have notable events changed or temporal trends changed?

107-109: From the introduction it is unclear to me what the diversity date is that the authors are trying to understand. What was this based on (e.g. range maps), how was it compiled, what groups does it contain, etc. Helping the reader now with a slightly expanded description of the methods will help the interpretation of the results.

"In general, I'm a little confused that all vertebrates seem to be lumped together in one analysis. Jetz and Fine also showed how it differed between vertebrate groups and between ecto- and endotherms. Given that they differ widely in their ecology and dispersal ability, historical factors might have different effects." Was my initial thought until I finally spotted the analysis in Figure 2B (comment below) - I'll leave this here as I still agree with this sentiment because it was difficult to find the results and they are not discussed still in much detail.

155: The first time that it is mentioned that multiple vertebrate classes are studied, but not mentioned again? What is the interpretation of these results?

170: Why the past 35 Ma and not the entire Cenozoic?

172-174: What test are the correlations based on?

249-252: Although an important message, do the authors require four paragraphs to discuss how biomes have changed through time?

256-258: The sentence is a bit difficult to parse. Consider rephrasing to make it clearer what Jetz and Fine found versus your work.

Additionally, the authors here acknowledge the difference between ecto- and endotherms, but was any such analysis performed in the current study to establish any contradictions or similarities?

265-269: This is again a paragraph that discusses the biomes and not the implications of the results.

355: "areal"

357: Which realms and how were they divided? Many methods exist that can be used to delineate them and different data sources. Is this interchangeably used with biome?

387-388: How many of how many total species?

374-382: with the information provided it is unclear to me what the phylogenies were used for.

400-402: I finally get where that 35 Ma comes from (L170 comments). Saying that, what is the justification for this? As far as I know, the Neogene started ~23 Ma and not 15 Ma, and the Oligocene ~34 Ma. The temporal resolution may play a role here but that wouldn't explain the Neogene.

Are the geological time scales properly matched up between the different climate models?

Jablonski, D., Roy, K. & Valentine, J.W. (2006). Out of the tropics: Evolutionary dynamics of the latitudinal diversity gradient. Science, 314, 102-106.

Pigot, A.L. & Tobias, J.A. (2013). Species interactions constrain geographic range expansion over evolutionary time. Ecology Letters, 16, 330-338.

Rabosky, D.L., Chang, J., Title, P.O., Cowman, P.F., Sallan, L., Friedman, M., et al. (2018). An inverse latitudinal gradient in speciation rate for marine fishes. Nature, 559, 392-395.

Reijenga, B.R., Etienne, R.S., Murrell, D.J. & Pigot, A.L. (2025). Speciation history shapes patterns of assemblage species richness in birds. bioRxiv

Stephens, P.R. & Wiens, J.J. (2003). Explaining species richness from continents to communities: The time-for-speciation effect in emydid turtles. American Naturalist, 161, 112-128.

Weir, J.T. & Schluter, D. (2007). The latitudinal gradient in recent speciation and extinction rates of birds and mammals. Science, 315, 1574-1576.

# Author comment: Author response to Decision Letter 2

**DOI:** 10.1371/journal.pbio.3004077.r003

9 Jun 2026

Attachment Submitted filename: Skeels_etal_2026_PLOS_Response_to_Reviewers.odt

# Peer review: Decision Letter 2

**DOI:** 10.1371/journal.pbio.3004077.r004

**Authors:** Ankiit Ahluwalia

13 Aug 2026

Dear Dr Skeels,

Thank you for your patience while we considered your revised manuscript "High-resolution paleobiome reconstructions highlight ecological and historical dynamics underpinning global terrestrial vertebrate diversity" for consideration as a Research Article at PLOS Biology. Your revised study has now been evaluated by the PLOS Biology editors, the Academic Editor and the original reviewers.

In light of the reviews, which you will find at the end of this email, we are pleased to offer you the opportunity to address the comments from the reviewers in a revision that we anticipate should not take you very long. We will then assess your revised manuscript and your response to the reviewers' comments with our Academic Editor aiming to avoid further rounds of peer-review, although we might need to consult with the reviewers, depending on the nature of the revisions.

As you can see, Reviewer1 was overall enthusiastic and noted that their two major concerns had been addressed very satisfactorily. These concerns related to discrepancies among the models used to reconstruct biome history and to the theoretical foundation of the findings in the previous version (e.g., framing the results within the context of equilibrium biodiversity dynamics). The reviewer suggested strengthening the use of references and provided several relatively minor comments aimed at further improving the manuscript. Reviewer2 appreciated your efforts but remained unsatisfied with the manuscript. The reviewer encouraged you to conduct additional analyses and provide further interpretation. These concerns were primarily related to the validation of the models and comparisons with Jetz and Fine (2012), particularly regarding bioregions. The reviewer suggested carrying out additional analyses to better align the results with the bioregions described by Jetz and Fine. R3 acknowledged that their previous concerns had been adequately addressed.

In addition to these revisions, you may need to complete some formatting changes, which you will receive in a follow up email. A member of our team will be in touch with a set of requests shortly. If you do not receive a separate email within a few days, please assume that checks have been completed, and no additional changes are required.

We expect to receive your revised manuscript within 1 month. Please email us (plosbiology@plos.org) if you have any questions or concerns, or would like to request an extension.

At this stage, your manuscript remains formally under active consideration at our journal; please notify us by email if you do not intend to submit a revision so that we withdraw the manuscript.

**IMPORTANT - SUBMITTING YOUR REVISION**

Your revisions should address the specific points made by each reviewer. Please submit the following files along with your revised manuscript:

1. A 'Response to Reviewers' file - this should detail your responses to the editorial requests, present a point-by-point response to all of the reviewers' comments, and indicate the changes made to the manuscript.

*NOTE: In your point-by-point response to the reviewers, please provide the full context of each review. Do not selectively quote paragraphs or sentences to reply to. The entire set of reviewer comments should be present in full and each specific point should be responded to individually.

You should also cite any additional relevant literature that has been published since the original submission and mention any additional citations in your response.

2. In addition to a clean copy of the manuscript, please also upload a 'track-changes' version of your manuscript that specifies the edits made. This should be uploaded as a "Revised Article with Changes Highlighted " file type.

*Resubmission Checklist*

When you are ready to resubmit your revised manuscript, please refer to this resubmission checklist: https://plos.io/Biology_Checklist

To submit a revised version of your manuscript, please go to https://www.editorialmanager.com/pbiology/ and log in as an Author. Click the link labelled 'Submissions Needing Revision' where you will find your submission record.

Please make sure to read the following important policies and guidelines while preparing your revision:

*Published Peer Review*

Please note while forming your response, if your article is accepted, you may have the opportunity to make the peer review history publicly available. The record will include editor decision letters (with reviews) and your responses to reviewer comments. If eligible, we will contact you to opt in or out. Please see here for more details:

https://blogs.plos.org/plos/2019/05/plos-journals-now-open-for-published-peer-review/

*PLOS Data Policy*

Please note that as a condition of publication PLOS' data policy (http://journals.plos.org/plosbiology/s/data-availability) requires that you make available all data used to draw the conclusions arrived at in your manuscript. If you have not already done so, you must include any data used in your manuscript either in appropriate repositories, within the body of the manuscript, or as supporting information (N.B. this includes any numerical values that were used to generate graphs, histograms etc.). For an example see here: http://www.plosbiology.org/article/info%3Adoi%2F10.1371%2Fjournal.pbio.1001908#s5

*Blot and Gel Data Policy*

We require the original, uncropped and minimally adjusted images supporting all blot and gel results reported in an article's figures or Supporting Information files. We will require these files before a manuscript can be accepted so please prepare them now, if you have not already uploaded them. Please carefully read our guidelines for how to prepare and upload this data: https://journals.plos.org/plosbiology/s/figures#loc-blot-and-gel-reporting-requirements

*Protocols deposition*

To enhance the reproducibility of your results, we recommend that if applicable you deposit your laboratory protocols in protocols.io, where a protocol can be assigned its own identifier (DOI) such that it can be cited independently in the future. Additionally, PLOS ONE offers an option for publishing peer-reviewed Lab Protocol articles, which describe protocols hosted on protocols.io. Read more information on sharing protocols at https://plos.org/protocols?utm_medium=editorial-email&utm_source=authorletters&utm_campaign=protocols

Thank you again for your submission to our journal. We hope that our editorial process has been constructive thus far, and we welcome your feedback at any time. Please don't hesitate to contact us if you have any questions or comments.

Sincerely,

Ankiit

Ankiit Ahluwalia,

PLOS Biology

aahluwalia@plos.org

----------------------------------------------------------------

REVIEWS:

Reviewer #1: In my previous review, I had two major concerns, and both have been very satisfactorily addressed. First, I pointed out the discrepancies between the models reconstructing the history of biomes and asked if these reconstructions are reliable and if they can be used for the intended inferences. The authors have now taken this very seriously, and provide a nice overview of the models, their similarities, differences and limitations. It makes the whole study much more robust and convincing, and it itself is very valuable - and quite novel and surprising.

Second, I was not entirely happy with the theoretical foundation of the findings in the previous version. Now the authors frame it using equilibrium biodiversity dynamics, and it is very clear. In this respect, I would still ask for a slightly better treatment of the literature - perhaps not by citing more references, but a bit better working with them. However, I am certainly biased in this respect, as I devoted last 15 years to the formalization and development of the equilibrium theory of biodiversity dynamics. I would just expect that some references could be put in the text at places where the authors describe the dynamics which is in precise accord with these formalizations, admitting that the findings agree with the predictions based on these formalizations. Also, I have spotted that (from my point of view) the key reference in this respect occurs in reference lists twice, as ref. 58 and ref. 72 - did the authors want to cite as the other reference the newer paper of the same set of authors (https://doi.org/10.1016/j.tree.2026.05.011), which is a response to a comment to ref. 58? I appreciate the theoretical treatment, I would just be more happy if the context of previous theoretical results would be better integrated.

Perhaps more importantly, I feel it would be good to better utilize (in the Discussion section) the recent paper Cerezer et al. 2026 published also in PLoS Biology (now cited as ref. 67; note that the reference in the list contains by mistake the name of the editor). This paper quite closely mirrors the findings presented by the authors, including specific results for different taxa (e.g. different factors affecting Squamates in comparison to other groups). Indeed, both these studies appear complementary - both are dealing with historical vs. equilibrium explanations of global vertebrate diversity patterns, although Cerezer et al. use phylogenetic information to infer history, while Skeels et al. use historical reconstruction of biomes - but both converge to exactly the same conclusions, which seems nice.

I have also a few concrete comments to particular places in the text as follows; all of them are relatively minor and aim just to an improvement of the text which is already quite good and potentially important.

Lines 56-60: Here some reference concerning the equilibrium theory would seem very appropriate, as this idea has been recently thoroughly developed and formalized, as mentioned above.

Line 67: Missing "of": "of biodiversity".

Line 84: "tropical biomes are spatially constricted by arid conditions" - yes, but some arid biomes are also tropical, right? At least some savannas are arid. I know Koppen classification distinguish tropical and arid biomes, but this sentence could be misleading.

Lines 100-101: Here it came to my mind that some other references could be also appropriate, as this process is described in ref. 58, and the concept of carrying capacity is not trivial, as described in Storch & Okie 2019, GEB 28: 1519. Carrying capacity is also mentioned on line 314. Again, not a crucial issue.

Line 145: This subtitle does not make sense, as there is no other subtitle in the chapter. I would recommend splitting the chapter to several subchapters with respective subtitles.

Line 231: It is unclear, do you mean "had only marginally better fit" (instead of "was…poorer fit") or "provided only marginally better fit"?

Fig. 3: Do I read it correctly that the best model (except for Squamates) included only productivity and temperature, and not area? Isn't it worth discussing? Also, the figure should be redrawn; now too much space is devoted to panel A (which can be squeezed in the left-right direction), while the crucial results in panels B and C are too small, and especially the font of the labels is extremely tiny.

Lines 267-268: Although I do appreciate the new treatment of biomes and their historical reconstructions, it came to my mind whether the historical (e.g. Eocene) "temperate" forests are really analogical to contemporary temperate forests. As far as I know, these forests, according to fossil record, contained fauna which is now considered quite tropical. It could be discussed as a potential caveat, but it may not be necessary.

Line 272: Missing "of": "of rainforest biome area".

Lines 318-325: This description of equilibrium diversity dynamics is not a general description of any diversity-dependent dynamics, but it is a bit more specific, describing a particular depiction formalized by the Equilibrium Theory of Biodiversity Dynamics, ETBD (developed specifically in references 5, 56, 58). It is OK, and I of course appreciate it, being the author of it, but for a reader who would not be fully familiar with it it could be better to clarify it, since other concepts do not necessarily assume that diversity-dependence acts via population size-dependence of extinction and/or speciation, which is the assumption of ETBD.

Lines 335-337: The observation that diversification (actually speciation, as extinction cannot be reliably measured) rates are higher in high latitudes can be also due to higher turnover in equilibrium/ carrying capacity (see ref 58 for details), i.e. not only due to out-of-equilibrium state of high latitudes. Both these views are reasonable.

Lines 347-353: This is indeed exactly equivalent to the argumentation in ref. 58.

Lines 370-374: Cerezer et al. 2026, mentioned above, provide alternative explanation for a poor effect of productivity on Squamate diversity, namely that NPP is not a good surrogate for resource availability for purely secondary consumers like Squamates. I am not saying it is necessary the best explanation of the different correlates of Squamate diversity (in comparison to mammals, amphibians and birds), but it may be worth considering.

Lines 545-549: The description of the models and model selection should be expanded. It is not clear whether all possible combinations of variables were tested (156 models do not seem to include all the possible combinations), and how (based on which criteria) the 13 models were selected. I am aware that most of this information is in the supplementary material, but the basic procedures should be also in the main text.

David Storch

Reviewer #2: I appreciate the authors' efforts in producing their revised manuscript. They redid most of their analyses and rewrote the introduction and discussion in line with the reviewers' constructive criticisms. However, I still find myself unsatisfied with the manuscript and would like the authors to consider further analyses and interpretation. Although I wouldn't want my objections to sink the paper, I do think at the very least that the authors should include the following points in their final submission as caveats.

First, I don't understand how we are supposed to evaluate the "validation" of their models that the authors now include in Figure S6. How does one decide that the agreement between the models and expert proxy-based reconstruction is "substantial" or "moderate" as the authors assert in the text (lines 167-186)? And since the expert proxy-based maps are only drawn for the realm (tropical -- not the Koppen biomes) how were these disagreements assessed? (see my criticism below on choice of Koppen biomes). The most important areas of disagreement will be between known paleobotanical localities that show tropical moist forest taxa that the paleoclimate models are not showing as "tropical rain forest or monsoon forest" -- In my opinion, the whole reason that the authors are showing better agreement with present day models vs time-Area measures is because the paleoclimate models they are using are wildly overestimating the arid areas and underestimating tropical rain forest areas. Looking at the tropical Americas and the Amazon, it is hard to reconcile everything I've learned from Carina Hoorn's and Kathy Willis and Alan Graham and Carlos Jaramillo's papers with these new paleoclimatic maps. In Figure 1, there is no rainforest in the Amazon 30 million years ago! Inconceivable! Similarly, experts in Mediterranean climates estimate that the biome is around 3 million years old (maximum 10 million years) -- and the paleomaps here show substantial Mediterranean ecosystems going back well into the Oligocene!

Second, and probably even more important -- it is hard to compare Jetz and Fine 2012 to the current analysis because the Skeels et al. paper includes many more biome-areas than we did. This is problematic for a couple different reasons. First of all, in Jetz and Fine 2012 we made the case that we were trying to define the "evolutionary arenas" that influenced the speciation and extinction of all resident taxa and drove the regional patterns of diversity. To justify these 32 "bioregions" we generated species lists of the vertebrate groups for each biome within continents (the bioregions -- see Figure 1 in Jetz and Fine 2012). Importantly, we compared the betadiversity (Jaccard similarity) among bioregions to make sure that each biome area within continents represented an independent evolutionary unit. Overlap in vertebrates among bioregions was 0% for all taxa except for birds (which was 4%).

The Skeels et al. paper includes 57 bioregions (listed in Figure 2). Although the authors tell us that they made sure that each species in their analysis only was counted in one region -- they do not report what the % overlap is among their 57 bioregions. I am guessing that the overlap would be substantial -- especially between tropical rainforest and tropical monsoonal forest, for example but probably within most of the Koppen biomes within climate zones. Moreover, it is hard to even reconcile the bio-regions we included in Jetz and Fine 2012 with these Koppen biomes. In Jetz and Fine, we lumped Tropical rainforest and seasonal moist forest to be one bioregion per continent (tropical moist forest) but separate from tropical dry forest and savanna. This agreed with the species overlap data. Here we have tropical rainforest separated from tropical monsoonal forest as well as tropical savanna. Where is tropical dry forest? Also, where are grasslands?

Would it be possible for the authors to lump the Koppen biomes into categories that more clearly match the 32 bioregions in Jetz and Fine? It would be very interesting to see what the differences would be between the new paleoclimate maps and our hand-drawn maps -- but the current manuscript doesn't really let us compare because the number of bioregions is so different AND the paleoclimate reconstruction are different. I also have a hard time being able to justify a time-area analysis on the 57 Koppen biomes due to the lack of continuity of each of those Koppen biomes over the past 50 million years and the lack of independence as "evolutionary arenas" (i.e., I doubt that tropical rain forest and tropical monsoon forest were independently generating new taxa within continents). What does "endemic" even mean in the context of 57 Koppen biomes? At the very least the authors should make it very clear that the comparison to Jetz and Fine 2012 by the current analysis is incomplete due to the different number and definition of a bioregion and the authors should report the % overlap in vertebrate taxa among all of their Koppen biomes.

Finally, I find the whole equilibrium diversity and carrying capacity discussion to be overdone. You can have diversity tracking contemporary biome sizes due to short lag times how biome area influences speciation and extinction rates. Please see pages 376-377 in Fine (2015) for a different perspective.

Line 67 should read "distribution of biodiversity"

Line 86. Authors should say here that these models disagree with known paleobotanical localities

line 113 should say "trees" instead of "plants"

Reviewer #3: Dear Editor,

Skeels et al. have adequately resolved any of my previous concerns and I think their work now forms a valuable contribution to the broader field. My previous concerns primarily related to the framing of the results and the clarity of writing. Specifically, the discussion of ecological versus historical processes has been nuanced and the extended focus on biological interpretations of their results have improved the paper substantially. I have listed only a couple of minor points below that can easily be addressed.

L63-L64: "Ecological and historical explanations for diversity are not mutually exclusive, but sit along a continuum". Although I agree with the sentiment, I wasn't entirely sure about the phrasing here. Specifically, what sits along the continuum? Is it the explanations/hypotheses or is it the resulting diversity (e.g. X% caused by history and Y% by ecology?).

L124: "at paint"

Figure 1: Bit of a pet peeve, but why is HadCM3L-B followed by A and not the other way around? Additionally, is (B) 0 Ma the present or is it model output? If the former perhaps specify in the legend.

L252: plural or singular model?

L350: citation?

L456: have a?

# Author comment: Author response to Decision Letter 3

**DOI:** 10.1371/journal.pbio.3004077.r005

3 Sep 2026

Attachment Submitted filename: Skeels_etal_2026_PLOS_Reviewer_Response_R2.docx

# Peer review: Decision Letter 3

**DOI:** 10.1371/journal.pbio.3004077.r006

**Authors:** Ankiit Ahluwalia

2 Oct 2026

Dear Dr Skeels,

Thank you for your patience while we considered your revised manuscript "High-resolution paleobiome reconstructions highlight ecological and historical dynamics underpinning global terrestrial vertebrate diversity" for publication as a Research Article at PLOS Biology. This revised version of your manuscript has been evaluated by the PLOS Biology editors, the Academic Editor and the original reviewers.

Based on the reviews and on our Academic Editor's assessment of your revision, we are likely to accept this manuscript for publication, provided you satisfactorily address the remaining points raised by the reviewers (if any). Please also make sure to address the following data and other policy-related requests.

IMPORTANT - please attend to the following:

a) We need numerical values underlying data on Fig1A, Fig1B, Fig2A, Fig3A,B and Figs S1-S17. I note that you have a related Zenodo repository (https://doi.org/10.5281/zenodo.17595796); however, access is currently restricted, so I am unable to check your compliance with our data and code availability policies. Please make it publicly accessible so that I check your compliance with our policies.

b) Please clearly cite the location of the data in all relevant main and supplementary figure legends. For example, you can add: “The data and code needed to generate this figure can be found at doi.org/10.xxx/zenodo.xxxxx.” We understand that this may appear repetitive, but it helps make the figures and their legends more self-contained.

c) Please include the URLs of your funders in the Financial Disclosure statement.

d) Please make any custom code available, either as a supplementary file or as part of your Zenodo deposition.

As you address these items, please take this last chance to review your reference list to ensure that it is complete and correct. If you have cited papers that have been retracted, please include the rationale for doing so in the manuscript text, or remove these references and replace them with relevant current references. Any changes to the reference list should be mentioned in the cover letter that accompanies your revised manuscript.

In addition to these revisions, you may need to complete some formatting changes, which you will receive in a follow up email. A member of our team will be in touch with a set of requests shortly. If you do not receive a separate email within a few days, please assume that checks have been completed, and no additional changes are required.

We expect to receive your revised manuscript within two weeks.

To submit your revision, please go to https://www.editorialmanager.com/pbiology/ and log in as an Author. Click the link labelled 'Submissions Needing Revision' to find your submission record. Your revised submission must include the following:

- a cover letter that should detail your responses to any editorial requests, if applicable, and whether changes have been made to the reference list

- a Response to Reviewers file that provides a detailed response to the reviewers' comments (if applicable, if not applicable please do not delete your existing 'Response to Reviewers' file.)

- a track-changes file indicating any changes that you have made to the manuscript.

NOTE: If Supporting Information files are included with your article, note that these are not copyedited and will be published as they are submitted. Please ensure that these files are legible and of high quality (at least 300 dpi) in an easily accessible file format. For this reason, please be aware that any references listed in an SI file will not be indexed. For more information, see our Supporting Information guidelines:

https://journals.plos.org/plosbiology/s/supporting-information

*Published Peer Review History*

Please note that you may have the opportunity to make the peer review history publicly available. The record will include editor decision letters (with reviews) and your responses to reviewer comments. If eligible, we will contact you to opt in or out. Please see here for more details:

https://plos.org/published-peer-review-history/

*Press*

Should you, your institution's press office or the journal office choose to press release your paper, please ensure you have opted out of Early Article Posting on the submission form. We ask that you notify us as soon as possible if you or your institution is planning to press release the article.

*Protocols deposition*

To enhance the reproducibility of your results, we recommend that if applicable you deposit your laboratory protocols in protocols.io, where a protocol can be assigned its own identifier (DOI) such that it can be cited independently in the future. Additionally, PLOS ONE offers an option for publishing peer-reviewed Lab Protocol articles, which describe protocols hosted on protocols.io. Read more information on sharing protocols at https://plos.org/protocols?utm_medium=editorial-email&utm_source=authorletters&utm_campaign=protocols

Please do not hesitate to contact me should you have any questions.

Sincerely,

Ankiit

Ankiit Ahluwalia,

aahluwalia@plos.org

PLOS Biology

------------------------------------------------------------------------

****[DELETE AS APPROPRIATE]****

ETHICS STATEMENT:

-- Please include the full name of the IACUC/ethics committee that reviewed and approved the animal care and use protocol/permit/project license. Please also include an approval number.

-- Please include the specific national or international regulations/guidelines to which your animal care and use protocol adhered. Please note that institutional or accreditation organization guidelines (such as AAALAC) do not meet this requirement.

-- Please include information about the form of consent (written/oral) given for research involving human participants. All research involving human participants must have been approved by the authors' Institutional Review Board (IRB) or an equivalent committee, and must have been conducted according to the principles expressed in the Declaration of Helsinki.

------------------------------------------------------------------------

DATA POLICY:

You may be aware of the PLOS Data Policy, which requires that all data be made available without restriction: http://journals.plos.org/plosbiology/s/data-availability. For more information, please also see this editorial: http://dx.doi.org/10.1371/journal.pbio.1001797

Note that we do not require all raw data. Rather, we ask that all individual quantitative observations that underlie the data summarized in the figures and results of your paper be made available in one of the following forms:

1) Supplementary files (e.g., excel). Please ensure that all data files are uploaded as 'Supporting Information' and are invariably referred to (in the manuscript, figure legends, and the Description field when uploading your files) using the following format verbatim: S1 Data, S2 Data, etc. Multiple panels of a single or even several figures can be included as multiple sheets in one excel file that is saved using exactly the following convention: S1_Data.xlsx (using an underscore).

2) Deposition in a publicly available repository. Please also provide the accession code or a reviewer link so that we may view your data before publication.

Regardless of the method selected, please ensure that you provide the individual numerical values that underlie the summary data displayed in the following figure panels as they are essential for readers to assess your analysis and to reproduce it:

[Figs….]

NOTE: the numerical data provided should include all replicates AND the way in which the plotted mean and errors were derived (it should not present only the mean/average values).

Please also ensure that figure legends in your manuscript include information on where the underlying data can be found, and ensure your supplemental data file/s has a legend.

Please ensure that your Data Statement in the submission system accurately describes where your data can be found.

------------------------------------------------------------------------

APPROPRIATE DATA PRESENTATION?

(If you think the paper is not using best practice, add the following)

- Please ensure that you are using best practice for statistical reporting and data presentation. These are our guidelines https://journals.plos.org/plosbiology/s/best-practices-in-research-reporting#loc-statistical-reporting and a useful resource on data presentation https://journals.plos.org/plosbiology/article?id=10.1371/journal.pbio.1002128

- If you are reporting experiments where n ≤ 5, please plot each individual data point.

------------------------------------------------------------------------

CODE POLICY

Per journal policy, if you have generated any custom code during the course of this investigation, please make it available without restrictions. Please ensure that the code is sufficiently well documented and reusable, and that your Data Statement in the Editorial Manager submission system accurately describes where your code can be found. More information on our Code Policy, what and how to share can be found here: https://journals.plos.org/plosbiology/s/code-availability

Please note that we cannot accept sole deposition of code in GitHub, as this could be changed after publication. However, you can archive this version of your publicly available GitHub code to Zenodo. Once you do this, it will generate a DOI number, which you will need to provide in the Data Accessibility Statement (you are welcome to also provide the GitHub access information). See the process for doing this here: https://docs.github.com/en/repositories/archiving-a-github-repository/referencing-and-citing-content

------------------------------------------------------------------------

SPECIES INDICATED IN THE ABSTRACT?

- Please note that per journal policy, the model system/species studied should be clearly stated in the abstract of your manuscript.

**EDITOR, CONSIDER ALSO INCLUDING ORGANISM IN THE TITLE, IF SENSICAL/RELEVANT AND/OR TO AVOID BEING MISLEADING/OVERREACHING (not always preferred)**

------------------------------------------------------------------------

BLOT AND GEL REPORTING REQUIREMENTS:

We require the original, uncropped and minimally adjusted images supporting all blot and gel results reported in an article's figures or Supporting Information files. We will require these files before a manuscript can be accepted so please prepare and upload them now. Please carefully read our guidelines for how to prepare and upload this data: https://journals.plos.org/plosbiology/s/figures#loc-blot-and-gel-reporting-requirements

------------------------------------------------------------------------

DATA NOT SHOWN?

- Please note that per journal policy, we do not allow the mention of "data not shown", "personal communication", "manuscript in preparation" or other references to data that is not publicly available or contained within this manuscript. Please either remove mention of these data or provide figures presenting the results and the data underlying the figure(s).

------------------------------------------------------------------------

Reviewer remarks:

Reviewer #1: I am happy with the current version of the manuscript, the authors have addressed all my (minor) remaining concerns, and as far as I can tell, also the concerns of the other two referees. In fact, I really appreciate the effort the authors put into addressing all these concerns; it is now very satisfactory.

Reviewer #2: I'd like to thank the authors for their detailed and careful revisions to their manuscript and their point-by-point responses to my earlier comments. I'd like to just make a few comments about their five responses where they describe their new analyses on first-tier Koppen biomes and report the overlap among these 26 units.

First, thanks for doing this and demonstrating that the proxies and models all are showing effectively the same pattern -- stronger signal from present-day area and energy in squamate diversity. I think that these first-tier analyses are the ones that should be used in the paper as they actually do represent "evolutionary arenas" that is -- the biome units that represent regional diversity and derive from realm-specific processes of speciation and extinction (and dispersal). The second-tier Koppen biomes do not represent evolutionary arenas -- and in point 4 the authors give us the figures of endemism which range from medium levels for amphibians and reptiles and low and very low for mammals and birds. As such, I don't think that these smaller units can be tracked through time as continuous units given that they include a species pool that includes more than one second-tier Koppen biome.

However, while I think that this is a super important point, I don't want to delay publication of this manuscript by insisting on new revisions.

Paul Fine

# Author comment: Author response to Decision Letter 4

**DOI:** 10.1371/journal.pbio.3004077.r007

6 Oct 2026

Attachment Submitted filename: Skeels_etal_2026_PLOS_Reviewer_Response_R3.docx

# Editor report: Decision Letter 4

**DOI:** 10.1371/journal.pbio.3004077.r008

**Authors:** Ankiit Ahluwalia

13 Oct 2026

Dear Dr Skeels,

Thank you for the submission of your revised Research Article "High-resolution paleobiome reconstructions highlight ecological and historical dynamics underpinning global terrestrial vertebrate diversity" for publication in PLOS Biology. On behalf of my colleagues and the Academic Editor, Michel Loreau, I am pleased to say that we can in principle accept your manuscript for publication, provided you address any remaining formatting and reporting issues. These will be detailed in an email you should receive within 2-3 business days from our colleagues in the journal operations team; no action is required from you until then. Please note that we will not be able to formally accept your manuscript and schedule it for publication until you have completed any requested changes.

Please take a minute to log into Editorial Manager at http://www.editorialmanager.com/pbiology/, click the "Update My Information" link at the top of the page, and update your user information to ensure an efficient production process.

PRESS

We frequently collaborate with press offices. If your institution or institutions have a press office, please notify them about your upcoming paper at this point, to enable them to help maximise its impact. If the press office is planning to promote your findings, we would be grateful if they could coordinate with biologypress@plos.org. If you have previously opted in to the early version process, we ask that you notify us immediately of any press plans so that we may opt out on your behalf.

We also ask that you take this opportunity to read our Embargo Policy regarding the discussion, promotion and media coverage of work that is yet to be published by PLOS. As your manuscript is not yet published, it is bound by the conditions of our Embargo Policy. Please be aware that this policy is in place both to ensure that any press coverage of your article is fully substantiated and to provide a direct link between such coverage and the published work. For full details of our Embargo Policy, please visit http://www.plos.org/about/media-inquiries/embargo-policy/.

Thank you again for choosing PLOS Biology for publication and supporting Open Access publishing. We look forward to publishing your study.

Sincerely,

Ankiit Ahluwalia,

PLOS Biology

aahluwalia@plos.org
